# A Versatile Synthetic Affinity Probe Reveals Inhibitory Synapse Ultrastructure and Brain Connectivity**

**DOI:** 10.1002/anie.202202078

**Published:** 2022-05-06

**Authors:** Vladimir Khayenko, Clemens Schulte, Sara L. Reis, Orly Avraham, Cataldo Schietroma, Rafael Worschech, Noah F. Nordblom, Sonja Kachler, Carmen Villmann, Katrin G. Heinze, Andreas Schlosser, Ora Schueler‐Furman, Philip Tovote, Christian G. Specht, Hans M. Maric

**Affiliations:** ^1^ Rudolf Virchow Center Center for Integrative and Translational Bioimaging; University of Wuerzburg Josef-Schneider-Str. 2 97080 Wuerzburg Germany; ^2^ Institute of Clinical Neurobiology University Hospital Versbacher Str. 5 97078 Wuerzburg Germany; ^3^ Department of Microbiology and Molecular Genetics Institute for Medical Research Israel-Canada the Hebrew University Hadassah Medical School Jerusalem 91120 Israel; ^4^ Abbelight 191 Avenue Aristide Briand 94230 Cachan France; ^5^ Center of Mental Health University of Wuerzburg Margarete-Höppel-Platz 1 97080 Wuerzburg Germany; ^6^ Diseases and Hormones of the Nervous System (DHNS) Inserm U1195 Université Paris-Saclay 80 rue du Général Leclerc 94276 Le Kremlin-Bicêtre France

**Keywords:** Dimerization, Fluorescent Probes, Neuroscience, Peptides, Super-Resolution Microscopy

## Abstract

Visualization of inhibitory synapses requires protocol tailoring for different sample types and imaging techniques, and usually relies on genetic manipulation or the use of antibodies that underperform in tissue immunofluorescence. Starting from an endogenous ligand of gephyrin, a universal marker of the inhibitory synapse, we developed a short peptidic binder and dimerized it, significantly increasing affinity and selectivity. We further tailored fluorophores to the binder, yielding “Sylite”—a probe with outstanding signal‐to‐background ratio that outperforms antibodies in tissue staining with rapid and efficient penetration, mitigation of staining artifacts, and simplified handling. In super‐resolution microscopy Sylite precisely localizes the inhibitory synapse and enables nanoscale measurements. Sylite profiles inhibitory inputs and synapse sizes of excitatory and inhibitory neurons in the midbrain and combined with complimentary tracing techniques reveals the synaptic connectivity.

## Graphical abstract

A short dimerized peptidic probe for high contrast visualization of inhibitory synapses in neuron cultures and brain tissue is presented. The probe can be flexibly applied both for widefield and confocal 3D volumetric synapse visualization, for inhibitory circuits mapping in tissue and for super‐resolution imaging of synapses.

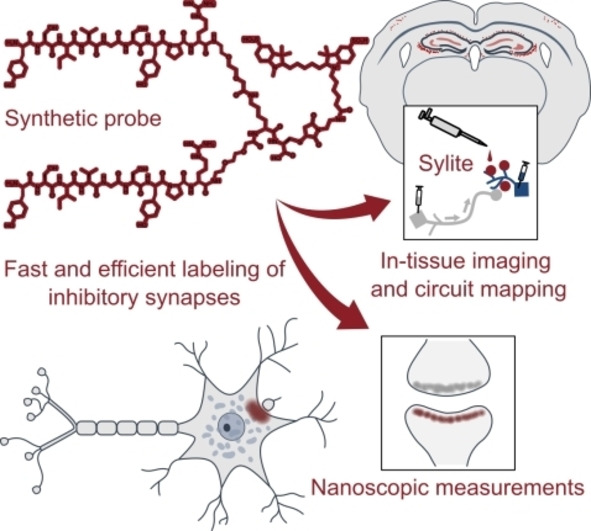

## Introduction

Reliable probes that label and visualize synapses are invaluable tools for clinical and fundamental neuroscience.^[1]^ Inhibitory synapses in the central nervous system (CNS) are either glycinergic or GABAergic and are commonly identified and visualized using several marker proteins such as vesicular GABA transporter[^2^, ^3^] and GABA_A_ receptor (GABA_A_R) γ2 subunit[^4^, ^5^] for GABAergic synapses, or the glycine receptor (GlyR) α1 subunit for glycinergic synapses.^[6]^ Yet, the common pan‐inhibitory synapse marker is the postsynaptic scaffold protein gephyrin that is present at glycinergic and GABAergic synapses alike.^[7]^ Gephyrin is an integral protein that stabilizes GlyRs and GABA_A_Rs at the postsynaptic density,[^8^, ^9^] and its concentration closely correlates with the number of inhibitory receptors and synaptic strength.[^2^, ^10^, ^11^, ^12^]

Gephyrin is often visualized using genetic tagging. eGFP, mCherry, mEos2 and other fluorescent proteins can be fused with gephyrin and expressed in cells via transfection or infection.[^6^, ^13^, ^14^] Fluorescent gephyrin chimeras have been successfully used for synapse studies both in fixed and live cell cultures,[^6^, ^13^, ^14^] as well as in conditional expression in transgenic animals,^[15]^ but an inherent drawback of induced secondary gephyrin expression are morphological and/or functional effects in cells.[^15^, ^16^, ^17^] To circumvent these drawbacks, two alternative methods of genetic tagging were applied. The first method involves knock‐in mice expressing mRFP‐gephyrin where expression levels, subcellular distribution of the endogenous protein and synaptic function are largely preserved.[^6^, ^12^] The second method consists in intracellular expression of eGFP fused anti‐gephyrin nanobody (recombinant antibody‐like light‐weight protein) that visualizes the inhibitory synapses in fixed and live cells.^[18]^ This approach can be especially effective when combined with a transcriptional control system that matches the expression level of the protein tag with that of its endogenous target, minimizing off‐target labeling and unwanted functional effects.^[19]^ In other words, genetic tagging methods need thorough evaluation of the impact of the genetic interference on the model and often require transcriptional control mechanisms. Furthermore, the availability of genetically tagged models is limited, especially of the knock‐in animals, and there are few options for multiplexing, due to the wide excitation and emission range of fluorescent proteins.

A powerful approach that bypasses some of the limitations of genetic tagging is immunostaining of endogenous proteins, a technique that is easily applicable to post vivo samples. Several antibodies have become the “gold standard” for gephyrin labeling and have been used for almost four decades.[^2^, ^20^, ^21^, ^22^] Yet the antibodies’ large size and their tendency to crosslink the target proteins can affect labeling performance, particularly in tissue and other intricate samples.[^23^, ^24^] In past years, several antibody alternatives have therefore been actively developed in order to replace or complement antibody staining, especially for immunohistochemistry and super‐resolution imaging.[^25^, ^26^] A recent study described the use of nanobodies as immunolabels for different neuronal proteins, including gephyrin, and showed with the example of Homer1, a marker for excitatory synapses, that nanobodies can successfully be used for tissue staining and, due to their small size, enhance the obtained spatial resolution in super‐resolution microscopy.^[18]^

A promising alternative to genetically encoded probes and antibodies are synthetic affinity probes such as nucleic acid derived aptamers, peptides and small molecules.^[27]^ Perhaps the best‐known examples of synthetic probes are the nuclear stains DAPI and Hoechst that have been around for half a century and are widely used for light microscopy.^[28]^ New synthetic probes for standard reference markers have been developed and commercialized, the most prominent being the SiR‐actin/tubulin for the cytoskeleton.^[29]^ These probes share similarities with nanobodies, like the small size, facilitating tissue penetration and improving spatial resolution, but unlike antibody‐like probes that are randomly generated against a protein fragment, synthetic probes can be rationally designed to bind specific isoforms or activity states of the target protein.^[30]^

A succinct summary of the inhibitory synapse visualization methods is shown in Table 1.

**Table 1:**
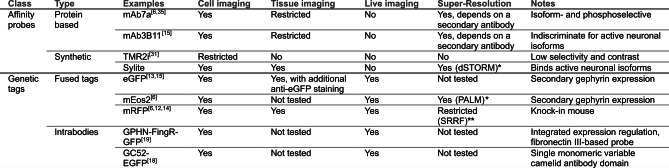
Summary of published labeling methods for gephyrin.

*dSTORM and Photoactivated localization microscopy (PALM) are single molecule localization techniques that require specific fluorophores that have stochastic blinking behavior under certain conditions. ≈20 nm average resolution.^[41]^ **Super‐resolution radial fluctuations (SRRF) is a fluorophore independent super‐resolution algorithm that analyses image sequences of one sample to generate a super‐resolution image with an average resolution of ≈110 to ≈200 nm.^[42]^

Here we outline the development of a new, synthetic, gephyrin‐binding probe and demonstrate its application in cell culture and tissue, where it acts as a versatile tool to visualize neuronal structures on various scales, from macro‐ to nanoscopic studies of the brain.

## Results and Discussion

### Evolution of Synthetic Gephyrin Probes

TMR2i is the only synthetic probe that has been successfully used to label gephyrin.^[31]^ This peptide probe is based on the intracellular loop of the GlyR β subunit, the strongest endogenous binder of the universal receptor binding pocket of gephyrin.[^32^, ^33^] Using peptide microarrays to explore the effects of GlyR β binding sequence multimerization, we deduced promising multivalent probe architectures (Figure S1, Tables S1,2). Then, we determined the amino acid exchanges that could strengthen the binding of the GlyR β loop core sequence to native gephyrin (Figure S1). Lastly, we identified the most successful probes by cell‐based‐imaging screening of an array of different fluorescent mono‐ and multivalent gephyrin binders (Table S3).

**Figure 1:**
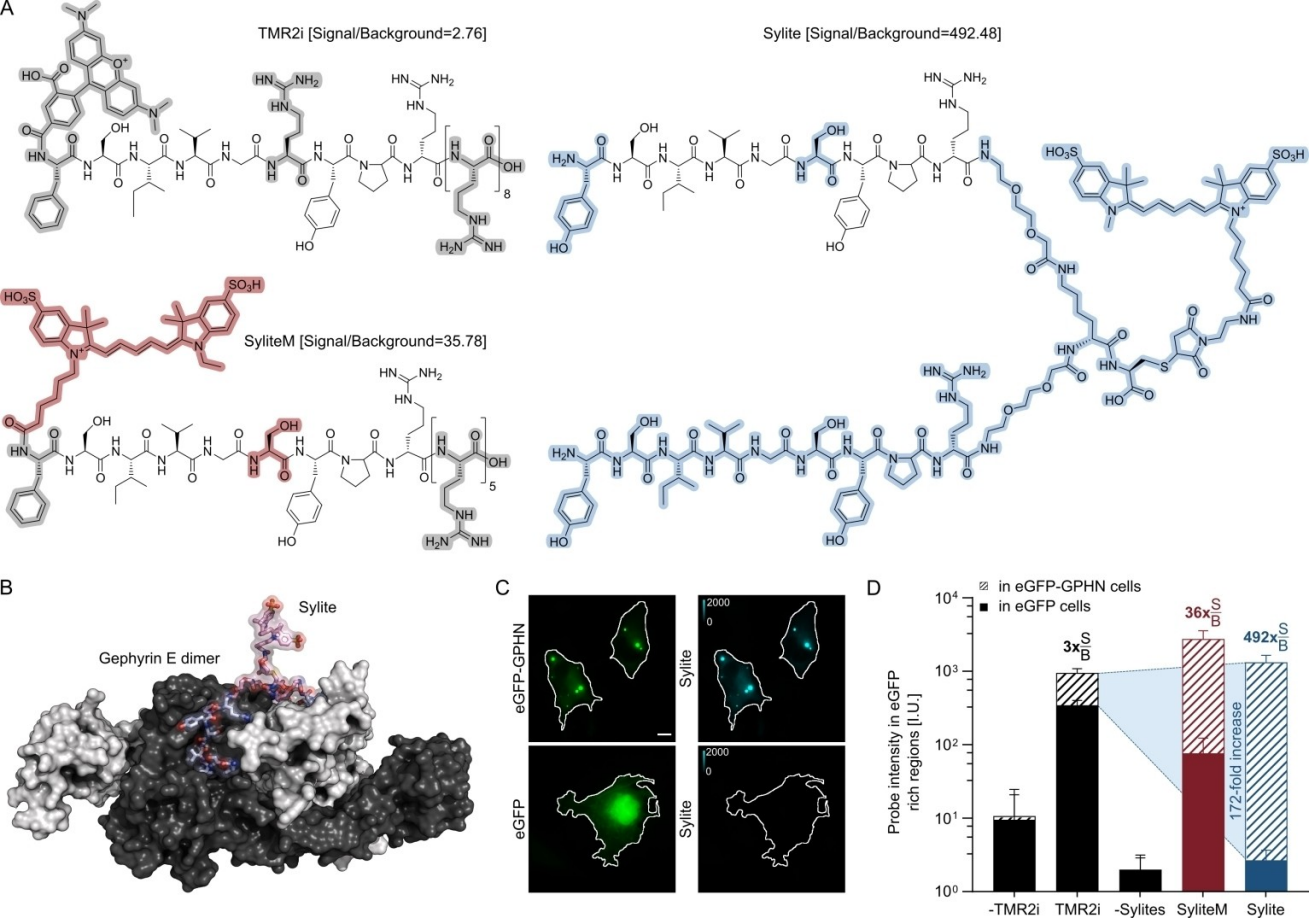
Evolution of peptide‐based gephyrin probes. A) Chemical structures of TMR2i, SyliteM and Sylite. The stepwise evolution of the TMR2i to Sylite involves fluorophore tailoring with binding sequence changes, resulting in an improvement of one order‐of‐magnitude in the SBR (SyliteM, in red are the changes, in gray are the obsolete components). Dimerization, additional sequence changes and the relocation of the fluorophore to the C terminus yields another order‐of‐magnitude improvement in SBR (Sylite, blue). B) Rosetta FlexPepDock structural model of Sylite bound to the gephyrin (GPHN) E domain dimer. In light blue is the binding sequence, in pink is the linker and the fluorophore; the two gephyrin E domains are shown in black and white. Sylite can simultaneously bind two gephyrin molecules. C) Fixed COS‐7 cells expressing either eGFP‐gephyrin or eGFP (green) stained with 50 nM of Sylite (cyan). Scale bar 10 μm. D) Labeling contrast of the synthetic peptide probes. The logarithmic Y axis represents the average signal intensity of the probe at eGFP‐rich regions of the COS‐7 cells. Sylite and SyliteM have 492 and 36 signal‐to‐background ratios (rounded), respectively. TMR2i has a target to off‐target labeling ratio of ≈3. The negative control (unlabeled cells) in the red channel is shown as “‐TMR2i”, that in the far‐red channel as “‐Sylites”. N≥8 samples per condition. Mean±SD.

Sylite, a dimeric probe with 9‐amino acid long binding sequences and a functional cysteine‐modified linker that enabled maleimide‐Cy5 conjugation showed the highest signal‐to‐background ratio (SBR) and colocalization with gephyrin (Figure S2, Figure S3A, B), whereas SyliteM was the best monovalent counterpart (Figure 1A). To apprehend Sylite's mode of binding and its superiority to other dimers, we used in‐silico modeling of the Sylite‐gephyrin interaction. The model implies simultaneous attachment to two gephyrin molecules and defines the minimal binding arm length allowing the divalent interaction, a prerequisite only met by Sylite (Figure 1B).

**Figure 2:**
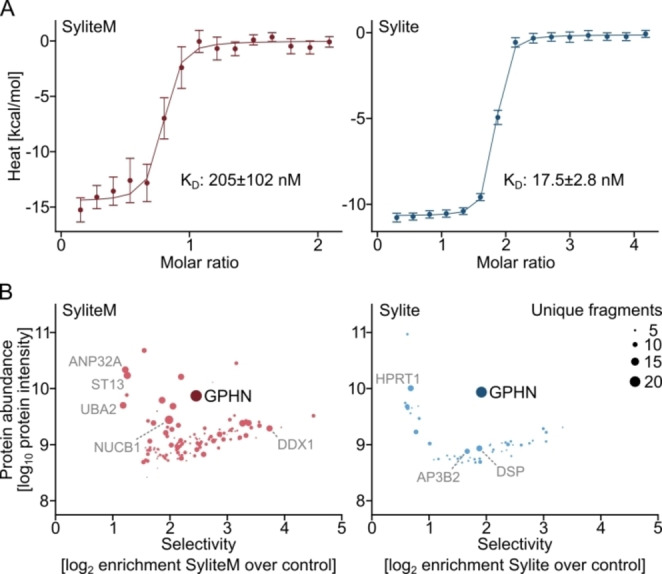
Probe dimerization enhances affinity and selectivity to gephyrin. A) ITC measured heat signature of Sylite and SyliteM titrated with gephyrin E domain. Both probes exhibit nanomolar affinity, with the dimeric Sylite having 10‐fold affinity increase over the monomer. N=3. Error bars are auto generated with NITPIC software and indicate SD. B) Quantitative mass spectrometric analysis of Sylite and SyliteM pull‐downs. Non‐fluorescent versions of Sylite and SyliteM were used to pull down proteins from mouse brain homogenate and the protein fractions were subsequently digested and analyzed with LC‐MS/MS. The size of the circle corresponds to the number of unique peptides identified for each protein. Left: SyliteM retains additional proteins that have high intensity and abundantly represented in the pool, even though gephyrin is the most prominent. In grey are shown several representative proteins having over 10 fragments confirming their identity. ANP32 A: acidic leucine‐rich nuclear phosphoprotein 32 family member A, regulatory protein; ST13: suppression of tumorigenicity 13, adaptor protein; UBA2: ubiquitin like modifier activating enzyme 2, posttranslational modification of proteins; NUCB1: nucleobindin 1, calcium‐binding protein; DDX1: DEAD‐box helicase 1, RNA helicase. Right: Gephyrin is the only protein with high abundance, selectivity and multiple fragments in the Sylite pull‐down. HPRT1: hypoxanthine‐guanine phosphoribosyltransferase, generates purine nucleotides; AP3B2: clathrin‐associated adaptor protein complex 3, involved in generation of synaptic vesicles; DSP: Desmoplakin, cell adhesion protein.

In a follow‐up assay we evaluated TMR2i, SyliteM and Sylite for gephyrin visualization. COS‐7 cells expressing either eGFP‐gephyrin or eGFP alone were fixed and stained with the fluorescent probes. The dimeric Sylite showed both complete correlation with gephyrin and a remarkable SBR of 492, a 172‐fold increase over TMR2i and 14‐fold increase over SyliteM (Figure 1C, D, Figure S3C). Next, using isothermal titration calorimetry (ITC) with the purified gephyrin E domain, we determined a *K*_D_ of 17.5 nM for Sylite, an 11‐fold affinity increase over the monomeric SyliteM (205 nM), indicating high probe affinity and confirming the expected 1 : 2 binding stoichiometry of the probe, in line with its dimeric design (Figure 2A). Furthermore, mass‐spectrometric analysis of the interactome of Sylite confirmed its high selectivity for gephyrin within the whole brain proteome. Gephyrin was the only protein with high abundance, high enrichment and represented by multiple unique peptide fragments binding to Sylite. (Figure 2B, Table S4). The monovalent SyliteM probe retained several additional proteins, demonstrating that probe dimerization not only enhanced affinity but also target selectivity.

### Sylite Targets Functionally Active Isoforms of Gephyrin

**Figure 3:**
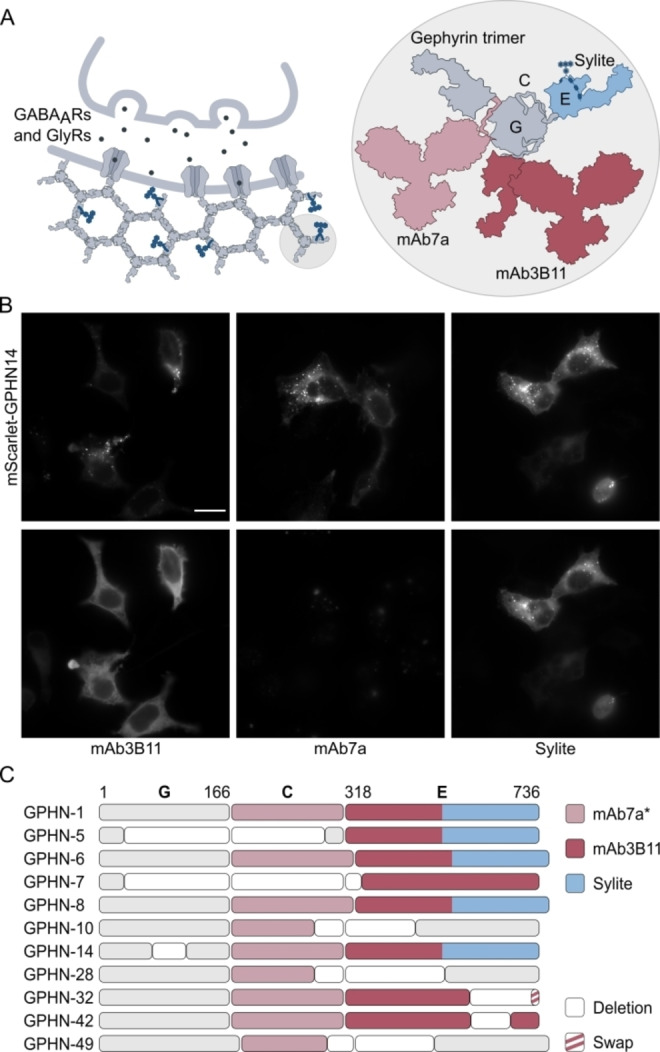
Sylite specifically labels gephyrin isoforms with receptor binding capability. A) Gephyrin anchors GlyRs and GABA_A_Rs at the post‐synapse. The protein consists of 3 regions: G domain, C unstructured linker region, E domain, where the receptor‐binding pocket is located and that is targeted by Sylite. Antibodies are raised against protein fragments that are not necessarily related to specific protein functions. B) Representative images of the gephyrin (GPHN) isoform labeling with Sylites and gephyrin antibodies. Top: Fixed HEK293 cells expressing mScarlet‐gephyrin isoform 14. Bottom: Co‐labeling of gephyrin with either mAb3B11, mAb7a, or Sylite. mAb3B11 stains gephyrin in the transfected cells but does not resolve the fine puncta visible in the top image. mAb7a does not label recombinant gephyrin in HEK293 cells. Sylite specifically labels mScarlet‐gephyrin and resolves the fine puncta where the recombinant protein accumulates. Scale bar 10 μm. C) Interaction of probes with different gephyrin isoforms. The isoform GPHN‐1 represents the primary structure of gephyrin.^[36]^ Blank boxes indicate deletions, elongated boxes additions, striped boxes substitutions. Sylite binds isoforms that contain a receptor binding pocket in the E domain. The antibodies target both receptor clustering competent and binding deficient isoforms. mAb7a (rose) binds a short linear *Ser270 phosphorylated epitope in the linker region (C), while mAb3B11 (raspberry) interacts with an epitope in the E domain. Sylite (blue) exclusively binds gephyrin isoforms with a receptor binding pocket in the E domain.

Gephyrin is a multifunctional protein with numerous isoforms and post‐translational modifications, some of which are specific for neurons, others have functions unrelated to neurotransmission, such as molybdenum cofactor biosynthesis in non‐neural tissues.^[34]^ Unlike antibodies that are raised against protein fragments that are not necessarily exclusive or related to specific protein activity, Sylite is a functional probe designed to bind receptor binding competent gephyrin isoforms, i.e., isoforms that exhibit functional roles in neurons (Figure 3A). Comparison of the binding profiles of eleven gephyrin isoforms (Table S5, Appendix 1) expressed in HEK293 cells revealed that both Sylite and SyliteM, but not the tested antibodies, exclusively label gephyrin isoforms that have GlyR and GABA_A_R binding capacity (Figure 3B, C, Figure S4). This suggests that Sylite is ideally suited to detect active synapses and quantify functionally relevant receptor binding sites. Interestingly, no gephyrin labeling was observed with the widely used mAb7a antibody in HEK293 cells, probably due to the phosphorylation state of gephyrin in the cells. Microarray profiling of mAb7a binding (Figure S5, Table S6) confirmed that in contrast to Sylite, mAb7a binding depends on the presence of a phosphorylated (pSer270) epitope in the linker region of gephyrin.^[35]^ Thus, mAb7a labels only a sub‐population of synaptic gephyrin isoforms and phosphorylation variants.

### Multicolor Toolbox for the Visualization of Inhibitory Synapses

**Figure 4:**
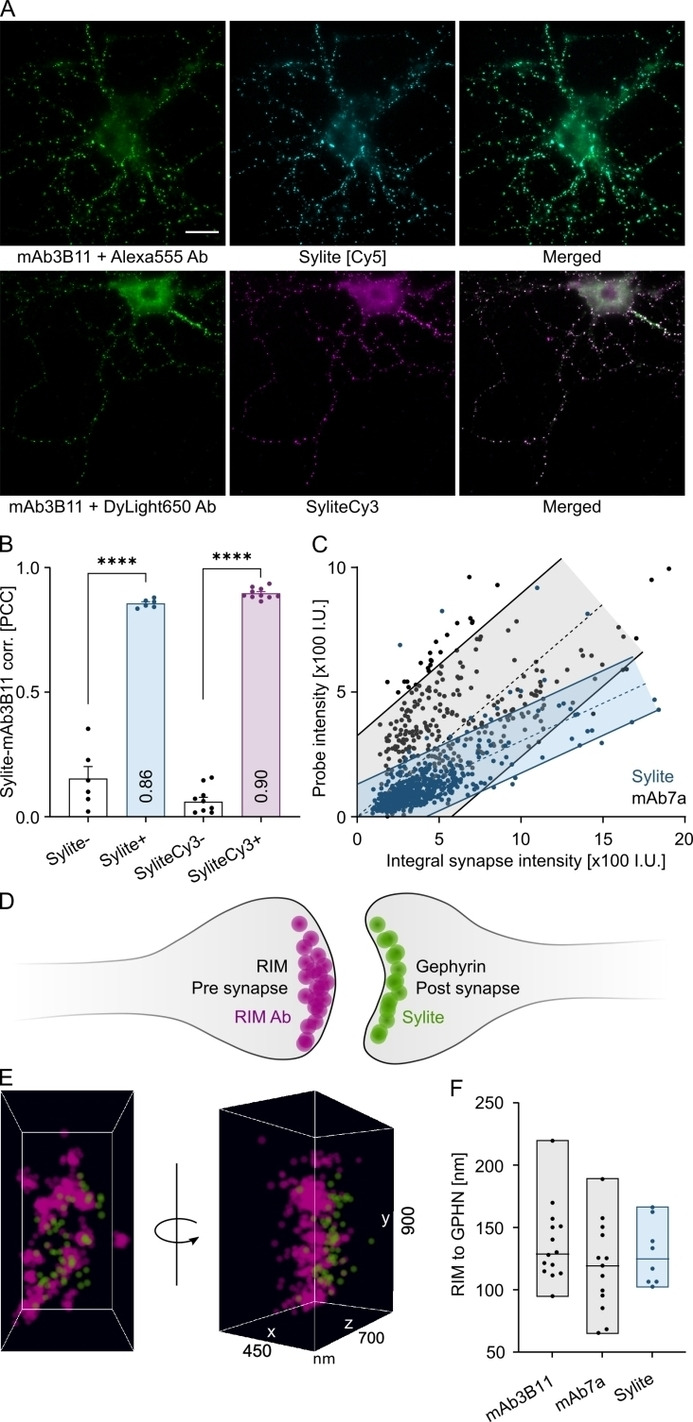
Multicolor probes for the visualization and super‐resolution studies of inhibitory synapses. A) Primary wild‐type hippocampal neurons fixed and stained with Sylite or SyliteCy3. Top: Co‐labeling with mAb3B11 and a secondary Alexa 555 conjugated antibody (green) and with Sylite (cyan). Bottom: Anti gephyrin mAb3B11 and secondary DyLight650 conjugated antibody (green) and SyliteCy3 co‐staining (magenta). Scale bar 10 μm. B) Pearson's correlation coefficients (PCC) of mAb3B11 and Sylite signals confirm high degrees of co‐localization. The negative control in the far‐red channel (unlabeled) is shown either by “SyliteCy3‐” for cells labeled only with SyliteCy3, or by “Sylite‐” for cells labeled only with mAb3B11 and Alexa555 secondary antibody. Significance determined with Welch's t‐test. *P*<0.0001. C) Intensity dependence of synapse labeling with mAb7a or Sylite compared to the internal reference signal mEos2‐gephyrin in infected neurons. Higher signal scattering is observed with mAb7a (grey), while Sylite (blue) has a constant and less variable linear labeling behavior. Shaded regions indicate the 90 % prediction interval. 10 pairs of images were used for each probe. D)–F) Super‐resolution imaging and nanometric measurements with Sylite. D) Neuronal synapse illustrating presynaptic RIM1/2 labeling using a CF680 secondary antibody (magenta) and postsynaptic gephyrin labeling with Sylite (green). E) Dual‐color dSTORM visualization of single molecule detections using spectral de‐mixing is shown as planar projection and *en face* view of a single synapse F) RIM to gephyrin center of mass distance measurements were conducted with RIM1/2‐CF680 and either gephyrin antibodies or Sylite. In all cases an average distance of ≈130 nm was determined. Bars indicate the full range of individual measurements, the in‐bar line indicates the median value.

In fluorescence microscopy simultaneous imaging of two or more spectral channels is typically necessary for an evaluation of a biological question. Having flexibility in fluorophore and spectral channel choice is advantageous as this can save time and resources, in particular the need to re‐adjust an experimental procedure or acquire new antibodies. We therefore produced a functional probe emitting in the red spectrum, SyliteCy3, expanding the spectral range of Sylites. SyliteCy3 performs comparably to Sylite: Colocalization analysis in mammalian cells expressing eGFP‐gephyrin showed a close linear relationship between the probe and gephyrin, while no correlation was observed in cells expressing eGFP only (Figure S6), confirming the selectivity of the probe for gephyrin. We next applied Sylites on primary neurons. Both Sylite and SyliteCy3 visualize the inhibitory synapses and the diffuse gephyrin in neuronal cell bodies, as confirmed by the co‐staining of primary hippocampal neurons with gephyrin 3B11 antibody (Figure 4A, B). Interestingly, the colocalization of Sylite with mAb7a was lower than with mAb3B11 (Figure S7A,B), leading us to investigate the nature of Sylite and mAb7a interaction with gephyrin in neurons. We stained cortical neurons expressing gephyrin‐mEos2 fluorescent protein chimera with Sylite and mAb7a. We then performed colocalization analysis and compared the mEos2 intensity of individual synapses with that of Sylite or mAb7a (detected with a secondary antibody). Although both probes colocalized well with recombinant gephyrin in neurons (Figure S7C,D), linear regression analysis of fluorescent intensities of synaptic puncta revealed a 2‐fold closer prediction interval for Sylite compared with mAb7a, indicating a linear and much closer correlation between Sylite and mEos2‐gephyrin signals (Figure 4C). The higher scattering observed with mAb7a on the other hand suggests that the antibody staining exhibits non‐linear scaling with synaptic gephyrin, in agreement with our previous finding that the mAb7a antibody specifically targets a phosphorylated variant of gephyrin. Taken together, our data demonstrate a linear, stoichiometric relationship between Sylite and gephyrin, making it suitable for quantitative microscopy.^[6]^

### Precise Super‐Resolution Pre‐ to Postsynaptic Distance Measurements with Sylite

Recent work showed that the presynaptic active zone (AZ) protein RIM forms trans‐synaptic nanocolumns with gephyrin.^[37]^ To determine the distance between the neuronal pre‐ and post‐synaptic elements, we labeled gephyrin and RIM in cortical neurons, then imaged the synapse using direct stochastic optical reconstruction microscopy (dSTORM), a super‐resolution microscopy technique based on single molecule detection (Figure 4D, E). RIM labeling was performed with primary anti‐RIM1/2 and CF680‐conjugated secondary antibodies and gephyrin was labeled either with Sylite or with commercial antibodies and AF647‐conjugated secondary antibodies. Dual‐color 3D‐dSTORM imaging using spectral de‐mixing showed that the Sylite detections closely match the distribution of RIM in the AZ, confirming the close association between sub‐synaptic domains containing RIM and gephyrin.[^2^, ^37^] The measured mean Euclidian distance between Sylite and RIM1/2‐CF680 detections was 129±24 nm (mean±SD), in agreement with the estimated molecular sizes separating the two proteins.^[37]^ The direct comparison with mAb7a and mAb3B11 gephyrin labeling confirmed that Sylite provides a precise read‐out of the location of the synaptic gephyrin scaffold and receptor binding sites at inhibitory synapses (Figure 4F).

### Sylite Displays Superior Tissue Penetration to Gephyrin Antibodies and Visualizes Hippocampal Synapses in 2D and 3D

**Figure 5:**
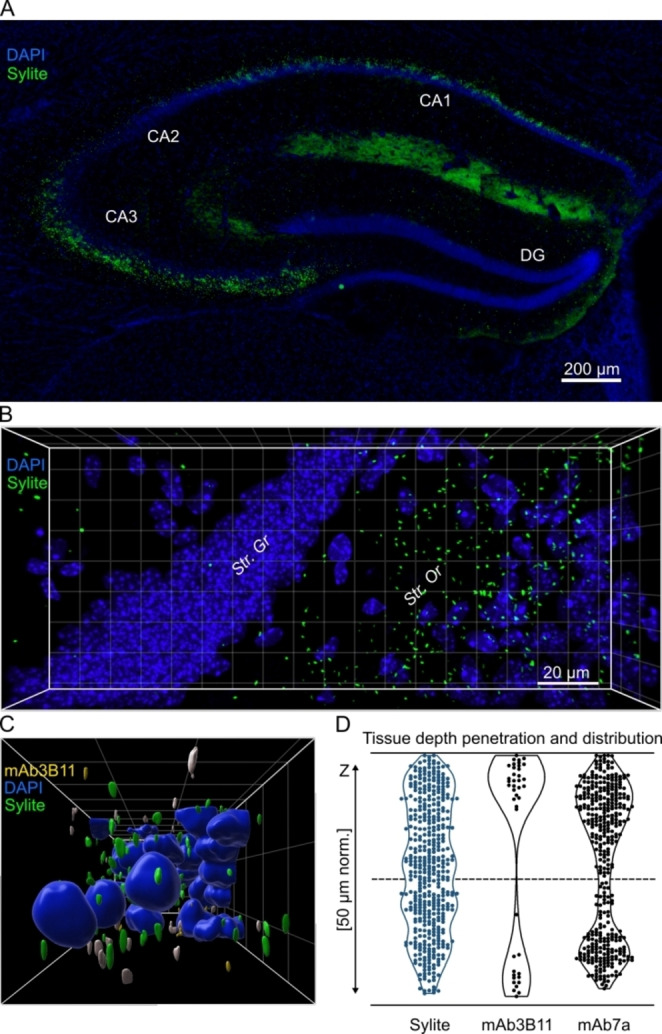
Sylite maps inhibitory synapses in brain tissue on macro‐ and microscale. A) Wide field 2D image of dorsal hippocampus section stained with DAPI nuclear staining (blue) and Sylite (green). A high density of distinct synaptic puncta is visible in CA1 and especially CA3 regions. B) 3D‐confocal microscopy of Sylite (24‐hours) staining in a ventral hippocampus section. Synapses appear in the *stratum oriens*. Str. Or—*stratum oriens*; Str. Gr—*stratum granulosum*. C) 3D volumetric representation of nuclei and inhibitory synapses. Side view of a rendered image stack from a section co‐labeled for gephyrin for 24 hours with mAb3B11 and with Sylite. Green—Sylite, yellow—mAb3B11, blue—DAPI nuclear staining. Sylite and mAb3B11 co‐labeled synapses are shown in white. Squares show a 10×10 μm grid. D) Distribution of Sylite and antibody labeling along the Z axis (depth) in 50 μm‐thick mouse hippocampal sections after 24‐hour staining. The top and bottom black lines indicate the section extremities, the dashed line the center. Violin plots represent the distribution of the detected clusters. The hourglass shape of antibody labeling indicates skewed antibody distribution, towards the surfaces of sections.

Tissue staining of inhibitory synapses is an elaborate and time‐consuming procedure that is generally limited to thin brain sections (≤16 μm) to obtain reliable labeling.^[7]^ Here, we demonstrate that Sylite effectively penetrates 50 μm‐thick tissue sections, achieving high‐contrast labeling within just one hour using a standard immunohistochemistry protocol. We visualized inhibitory synapses and their distribution using epifluorescence microscopy with 20x magnification, giving us a macro‐overview of the inhibitory synapse distribution in the hippocampus (Figure 5A). Next, we incubated the hippocampal sections for 1, 24 and 72 hours with Sylite, and either mAb3B11 or mAb7a, then imaged the sections with a confocal microscope, deconvoluted the image stacks, and reconstructed 3D images. Sylite‐visualized synapses were observed in the *stratum oriens* of the CA3 region of the ventral hippocampus, an area densely packed with inhibitory interneurons^[38]^ (Figure 5B, Movie S1).

Sylite detected synaptic clusters throughout the entire section, demonstrating a complete penetration of the probe already after 1 hour of incubation (Movie S2,3). Strikingly, even at 72 hours of incubation with Sylite we did not observe any significant background fluorescence (Movie S4,5). In contrast, after 24 hours, the antibody distribution appeared to have a “sandwich”‐like pattern, with the strongest labeling near the surfaces of the sections while the center remained largely unlabeled (Figure 5C, D, Movie S1,6). After 72 hours, antibodies appeared to lose binding specificity (Figure S8, Movie S4,5). This is seen by the drop in the voxel overlap between antibody and Sylite labeling from ≈0.4 for both mAb7a and mAb3B11 antibodies after 24 hours to ≈0.1 after 72 hours (Figure S8B). Lastly, 3D visualization of synapses obtained with Sylite showed smooth and well‐defined shapes of different sizes, in agreement with the known diversity of inhibitory synapses in the CNS.^[39]^ After 24 hours, the antibodies produced both smooth and amorphous clusters, and after 72 hours, this pattern changed to primarily amorphous clusters and loss of any observable localization in specific regions of the tissue section (Movie S7,8).

### Sylite Reveals Local Inhibitory Circuits in the Midbrain Periaqueductal Gray Region

**Figure 6:**
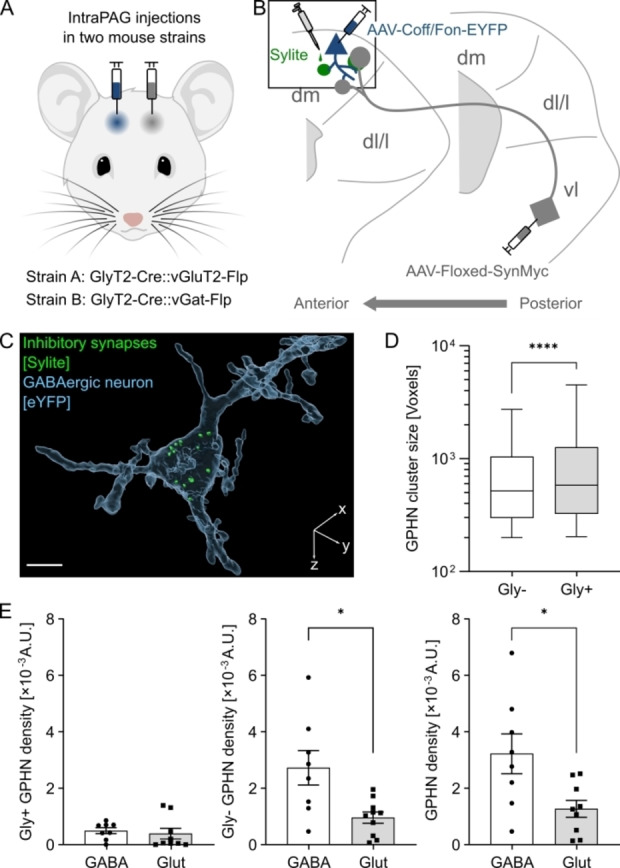
Mapping and characterization of inhibitory inputs in the periaqueductal gray. A) GlyT2‐Cre::vGluT2‐Flp or GlyT2‐Cre::vGat‐Flp recombinant mice were injected in posterior vlPAG with adeno‐associated virus (AAV) carrying a plasmid with Cre recombinase‐dependent Synaptophysin‐Myc chimera coding region (gray). An additional injection was done in the dmPAG with AAV carrying a plasmid with Cre‐off Flippase (Flp) recombinase‐on eYFP fluorescent protein coding region (blue). B) Anatomical tracing scheme of the injections and expression localizations in the periaqueductal gray. Glycinergic neurons project from vlPAG to dmPAG (gray). In dmPAG, depending on the mouse genotype, either GABAergic or glutamatergic neurons express soluble eYFP (blue). PAG brain sections from both mice strains were stained with Sylite (green). Box—the region of interest. C) 3D volumetric reconstruction of a single dmPAG GABAergic neuron cell body (light blue, transparent) from a brain section. Multiple gephyrin clusters (green) are found in the soma. Scale bar 10 μm. D) Glycinergic synapses are on average larger than non‐glycinergic synapses in dmPAG. Box (25th to 75th percentiles) and whiskers (5th to 95th percentiles) plot representing gephyrin (GPHN) cluster size distribution. Significance determined with unpaired t‐test with Welch's correction, *P*<0.0001. E) Higher inhibitory synapse density is observed in GABAergic neurons in dmPAG. Inhibitory synapse densities: total in‐neuron gephyrin volume (voxels) was divided by total neuron volume (voxels) in each tissue section of dmPAG and plotted, mean±SEM. The density of inhibitory synapses having glycinergic input (Gly+) does not differ between GABAergic and glutamatergic neurons (left), while higher non glycinergic (Gly‐) synapse density is observed in GABAergic neurons (middle). Total inhibitory synapse density in GABAergic neurons is higher than in glutamatergic neurons (right). Data sets were checked for normality with D'Agostino‐Pearson test. A single outlier was removed using Grubbs’ method with α=0.05. Significance determined with unpaired t‐test with Welch's correction, *P*<0.05, 8 (4 per animal) brain sections from GlyT2‐Cre::vGat‐Flp mice and 9 (3 per animal) sections from GlyT2‐Cre::vGluT2‐Fl mice were used.

The mammalian nervous system is composed of a complex network of specialized synaptic connections that coordinate the neuronal flow of information. The periaqueductal gray (PAG) is a midbrain region that plays an important role in orchestrating the defense reaction in response to a perceived threat. It has been postulated that intra‐PAG circuitry supports integration of multiple defense components, such as switching between active and passive behavioral coping patterns.^[40]^ However, the precise circuit mechanisms and their neuroanatomical substrates remain to be elucidated. To shed light on the putative role of local inhibitory glycinergic neurons in the ventrolateral PAG (vlPAG), we sought to clarify their intra‐PAG connectivity on the anatomical level using Sylite. We used cell‐type specific, virally mediated expression of immunolabels for synaptophysin, thereby allowing us to identify glycinergic pre‐synaptic terminals. We observed that these neurons locally project from the ventrolateral (vl) to the dorsomedial (dm) part of the PAG (Figure S9). We next aimed at identifying the target output cells of the glycinergic vlPAG neurons and characterize the inhibitory post‐synapse. To this purpose, we labeled either glutamatergic or GABAergic neurons by virally mediated expression of fluorescent proteins in the dmPAG and used Sylite to identify inhibitory post‐synaptic sites (Figure 6A–C, Figure S10). First, we assessed the size of the post‐synaptic densities visualized by Sylite. Gephyrin clusters targeted by vlPAG glycinergic were larger than those with non‐glycinergic inhibitory input (Figure 6D). This observation is in line with previous findings that reported larger individual gephyrin clusters in the spinal cord, a region rich in glycinergic neurons, compared with those in the cortex that are mostly GABAergic.^[6]^ Next, we investigated vlPAG glycinergic projections to dmPAG and identified the glycinergic presynapses close to both glutamatergic and GABAergic dmPAG gephyrin sites (Figure 6E, left), suggesting that vlPAG glycinergic neurons may exert inhibitory effects in the two functionally different dmPAG neuron classes. Interestingly, gephyrin density was higher in dmPAG GABAergic compared to glutamatergic neurons (Figure 6E, middle and right), suggesting that GABAergic dmPAG neurons receive overall strong inhibitory inputs. Taken together, our data demonstrate the usefulness of Sylite to identify target output cells of specific inhibitory neurons and further determine the precise location and size of their synapses.

## Conclusion

Through systematic improvements of the binding sequence, fluorescent dye tailoring and dimerization to enable simultaneous attachment to two gephyrin molecules, we produced a highly selective and highly affine probe for the inhibitory synapse. Unlike antibodies that are raised against protein fragments that generally do not relate to a specific protein function, Sylite is derived from the GlyR, an endogenous ligand of neuronal gephyrin, binding gephyrin isoforms that actively cluster receptors at the synapse. Sylite has a linker with cysteine allowing flexibility for conjugation of maleimide containing dyes, enabling straightforward derivatization of a potent binder to a fluorescent probe with imaging capabilities only limited to the fluorophore of choice. However, since fluorophores can have a significant impact on the binding properties of a short peptidic probe (Figure S2, S3), a pre‐application screening, like the one described for SyliteCy3 (Figure S6), is necessary.

Sylite is easily implemented in standard immunocytochemical and immunohistochemical assays, where it can be used as a substitute to or alongside antibodies, visualizing inhibitory synapses in a one‐step application similar to direct immunolabeling. Sylite, conjugated to the Cy5 fluorophore, is particularly useful for super‐resolution microscopy, enabling nanoscale studies of the inhibitory synapse, such as the pre‐to‐post synapse distance measurements conducted here. In tissue, presumably because of its small size, Sylite attains previously unachievable staining efficiency, enabling greatly simplified and accelerated tissue processing without imaging artifacts often observed with antibodies. Sylite can thus be applied for advanced and multiplexed anatomical tracing techniques that address cell‐type‐ and projection‐specific connectivity as part of circuit‐centered approaches to brain function. By and large, techniques that rely on viral delivery systems do not provide specific information on the post‐synaptic site, such as the identity of the cellular structures, their size and organization. Due to its high labeling efficiency in tissue, Sylite can therefore greatly contribute to an easier and more reliable identification of inhibitory postsynaptic densities and their size. In addition to identifying target cells and synaptic sizes of specific projection pathways as highlighted here, Sylite has potential to advance studies of the functional significance of precise, i.e., subcellular, synaptic localization.

Progressively complex neuroscientific research together with the technological advances in tissue imaging and super‐resolution microscopy create a strong demand for new probes for multiplex micro‐ and nanoscale studies of the brain.[^1^, ^43^] We expect that the rational probe development applied in this study, i.e. the evolution of an endogenous activity‐related ligand to a compact synthetic high‐affinity binder, probe multimerization for increased avidity, affinity and selectivity, as well as fluorophore screening and tailoring, will lead to new compact affinity probes for other key components of the nervous system, such as the excitatory postsynaptic scaffold protein PSD‐95 or the presynaptic scaffold protein Bassoon. In the future, Sylite derivatives may also be synergistically used with novel cell‐penetrating techniques[^44^, ^45^] that could facilitate live cell imaging and functional targeting, in vivo applications and pharmacological research. This, however, must be preceded by detailed biocompatibility and biotoxicity studies, including metabolic and chemical stability. According to our preliminary data, Sylite remains fully functional and chemically stable for at least 12 months, when stored as a frozen solution (Appendix 5).In conclusion, our findings establish Sylite as a powerful, versatile and reliable imaging tool for neuroscience. Advantages over the conventional affinity probes include selective labeling of functional inhibitory synapses with linear readout, rapid and straight‐forward tissue staining as well as compatibility with super‐resolution microscopy. Combined with advanced imaging techniques, synthetic probes, such as Sylite, open new research avenues in neuroscience, as they enable multiplex studies and achieve better localization precision, resolution and tissue staining.

## Conflict of interest

H.M.M. and V.K. hold a utility model # 20 2021 002 515 on Sylites. Sylites are commercialized by NanoTag Biotechnologies GmbH, Cat.#P4001. Ca.S. is employed at Abbelight.

1

## Supporting information

As a service to our authors and readers, this journal provides supporting information supplied by the authors. Such materials are peer reviewed and may be re‐organized for online delivery, but are not copy‐edited or typeset. Technical support issues arising from supporting information (other than missing files) should be addressed to the authors.

Supporting Information

Supporting Information

Supporting Information

Supporting Information

Supporting Information

Supporting Information

Supporting Information

Supporting Information

Supporting Information

## Data Availability

The data that support the findings of this study are available from the corresponding author upon reasonable request.
