# Supplementary material for: A Versatile Synthetic Affinity Probe Reveals Inhibitory Synapse Ultrastructure and Brain Connectivity
Source: Angew Chem Int Ed Engl. 2022 May 6;61(30):e202202078. doi: 10.1002/anie.202202078 (PMC9400903; doi:10.1002/anie.202202078)
Supplement: Supplementary file 1 — Supporting Information [file ANIE-61-0-s003.pdf]

Angewandte Chemie
Eine Zeitschrift der Gesellschaft Deutscher Chemiker

Supporting Information

A Versatile Synthetic Affinity Probe Reveals Inhibitory Synapse Ultrastructure and Brain Connectivity

V. Khayenko, C. Schulte, S. L. Reis, O. Avraham, C. Schietroma, R. Worschech, N. F. Nordblom, S. Kachler, C. Villmann, K. G. Heinze, A. Schlosser, O. Schueler-Furman, P. Tovote, C. G. Specht, H. M. Maric**

Table of contents

Materials and methods 4
Peptide synthesis and fluorophore conjugation 4
Purification and characterization of peptides and fluorescent probes 4
Protein expression and purification 5
Modeling of the Sylite/gephyrin supracomplex 5
Isothermal titration calorimetry (ITC) 5
Pulldowns with cellulose conjugated peptides 5
Mass spectrometric analysis of pulldowns 6
HEK293 and COS-7 cell cultures and transfection 7
Ethical approval statement 8
Culture and infection of primary neurons 8
Cell fixation and immunocytochemistry 9
Wide field fluorescence microscopy 9
2D image processing and analysis 10
Dual-color dSTORM super-resolution imaging 10
Brain section preparation and staining 11
Wide field and confocal imaging of brain sections 12
Animals 12
Injection of viruses and anatomical tracing 13
Synaptophysin-GFP image processing 14
dmPAG image processing 14
3D image processing 15

Supplementary Discussion 16
Probe development 16

Supplementary Figures 18
Figure S1. Optimization of probe sequence and multivalent architecture. 18
Figure S2. Microscopy-based probe evaluation. 19
Figure S3. Sylites visualize gephyrin with high contrast. 20
Figure S4. Sylites exclusively label gephyrin with receptor binding pocket. 21
Figure S5. Gephyrin mAb7a explicitly binds a phosphorylated epitope 22
Figure S6. SyliteCy3 colocalizes with eGFP-gephyrin in mammalian cells 23
Figure S7. Sylite and mAb7a staining of wild-type and recombinant neurons. 24
Figure S8. Sylites enable ultra-rapid synapse staining and visualization 25
Figure S9. Glycinergic neurons from vlPAG project locally to lPAG and dmPAG 26
Figure S10. Injection sites of the viruses in the periaqueductal gray 27

Supplementary Tables 28
Supplementary Table 1. Peptide microarray 1 sequences: Gephyrin binders 28
Supplementary Table 2. Linker building blocks 29
Supplementary Table 3. Fluorescent dyes 30
Supplementary Table 4. Sylite IP-MS analysis 31
Supplementary Table 5. Gephyrin isoform constructs 35
Supplementary Table 6. Peptide microarray 2 sequences: Gephyrin overlapping fragments 36

Supplementary References 38

Supplementary Notes 39
Supplemetary Method 1. Imaging-based screening 39
Supplemetary Method 2. Peptide microarray synthesis.. 39
Supplemetary Method 3. Microarray-based probe development 40
Appendix 1. Vector maps.. 41
Appendix 2. Macro and script for Image analysis 42
Appendix 3. Icy 2.0.3.0 protocol for single synapse segmentation and intensity recording .. 49
Appendix 4. Spectral profiles of Sylites.. 50
Appendix 5. Chromatographic and mass spectrometric probe validation............................. 51

Materials and methods

Unless otherwise noted, all resins and reagents were purchased from IRIS biotechnologies or Carl Roth and used without further purifications. All solvents used were HPLC grade. All water-sensitive reactions were performed in anhydrous solvents under positive pressure of argon. Statistical analysis was preformed using GraphPad Prism.

Peptide synthesis and fluorophore conjugation

Peptides were produced using standard solid phase peptide synthesis with Fmoc chemistry. Shortly, 2-chlorotrityl resin (1.6 mmol/g) was swollen in dry Dichloromethane (DCM) for 30 min., then, the desired amino acid (AA) (1eq) and the Boc-Gly-OH (1eq) with 4 eq. of dry N,N-Diisopropylethylamine (DIEA) were added to the resin slurry. After overnight reaction at RT with agitation, the resin was capped with MeOH and washed with DCM and Dimethylformamide (DMF). Deprotection and conjugation cycles followed, where 20% piperidine solution in DMF was used to remove the Fmoc protecting group. After washes the peptide chain was elongated by adding AA (4 eq.) with Ethyl cyanohydroxyiminoacetate (Oxyma, 4 eq.) and N,N'-Diisopropylcarbodiimide (DIC, 4 eq.). Capping was done with DIEA (50 eq.) and acetic anhydride (50 eq.) in N-Methyl-2-pyrrolidone for 30 min. Coupling efficiency was monitored by measuring the absorption of the dibenzofulvene–piperidine adduct after deprotection. The peptides were cleaved from the resin using a cocktail of 90% TFA, 5% H_2O, 5% Triisopropylsilane, for 4 hours at RT. The peptides were precipitated in ice-cold ether and then purified with HPLC and analyzed by LC-MS as described below.

The purified peptides or peptide dimers were conjugated with fluorophores either via NH_2-terminus using N-Hydroxysuccinimide (NHS) coupled dyes or via cysteine -SH side chain using maleimide coupled dyes. Shortly, for NHS coupling 1 eq. of peptide was dissolved in DMF with 3 eq. of DIEA and a fluorophore-NHS was added (1 eq for standard peptides, 2eq. for peptide dimers) and agitated overnight at 4°C. Maleimide conjugation was done with similar stoichiometry and conditions, with pH 7.4 PBS as a solvent and a minimal addition of DMSO to facilitate dissolution.

Purification and characterization of peptides and fluorescent probes

The fluorescent probes were purified from the crude reaction mix by reverse phase HPLC using water acetonitrile gradient with 0.1% formic acid (FA). LC-MS validation was performed with similar gradient and LC-MS grade solvents. Semi-preparative HPLC was performed on Shimadzu Prominence equipped with a diode-array detector (DAD) system using a C18 reverse-phase column (Phenomenex Onyx Monolithic HD-C18 100×4.6 mm or Onyx Monolithic C18 100×10 mm). Purity and structural identity were verified using a DAD equipped 1260 Infinity II HPLC with a C18 reverse-phase column (Onyx Monolithic C18

50×2 mm), coupled to a mass selective detector single quadruple system (Agilent Technologies) in ESI+ mode.

Protein expression and purification

Gephyrin P2 splice variant E domain (amino acids 318–736) was expressed in *E. Coli* and purified as described earlier[1]. Concisely, the protein was purified using via Intein-tag (Chitin beads, New England BioLabs), and after self-cleavage the protein was obtained by size-exclusion chromatography (SEC) column (HiLoad 16/600 Superdex 200pg, GE Healthcare) on an ÄKTA explorer system (GE Healthcare).

Modeling of the Sylite/gephyrin supracomplex

Generation of the Sylite bound to gephyrin E domain was carried out using the Rosetta FlexPepDock refinement protocol[2] using the crystal structure of gephyrin E domain bound to glycine receptor (GlyR) β subunit peptide (PDB ID: 4pd1) as a scaffold. Peptide residues where mutated using the Rosetta Fixed Backbone protocol to correspond to the binding sequence of Sylite. Following this process, the linker and dye were added to demonstrate the feasibility of the dimer formation.

Isothermal titration calorimetry (ITC)

Measurements were performed using an ITC200 (MicroCal) at 25 °C and 1,000 rotations per minute (rpm) stirring in PBS pH 7.4. Specifically, 40 µL of a 200 µM gephyrin E solution was titrated into the 200 µL sample cell containing 10 µM and 20 µM of Sylite and SyliteM, respectively. In each experiment, a volume of 2.5 µL of ligand was added at a time resulting in 15 injections and a final molar ratio between 1:2 (SyliteM) and 1:4 (Sylite). The dissociation constant (K_D) and stoichiometry (N) were obtained by data analysis using NITPIC, SEDPHAT and GUSSI[3]. Measurements were conducted three times for each probe and are given as mean values with their standard deviations.

Pulldowns with cellulose conjugated peptides

Cellulose membrane bound peptides were produced using µSPOT solid phase peptide synthesis[1]. After completion of the automated peptide synthesis, cellulose bound peptides side chains were deprotected with 90% TFA, 5% H_2O, 5% Triisopropylsilane for 3 hrs at RT, followed by washing with 5×2 mL H_2O. Afterwards, cellulose disks were left do dry overnight in a fume hood and stored at 4°C until use. For pulldowns, discs were first blocked in 2% (w/v) BSA in PBS for 1 hour at 25°C. Subsequently, one disc was incubated with 100 µL of mouse brain homogenate mixed with 100 µL of 10 mM TCEP in PBS for 45 min at 30°C. After washing with 3×300 µL PBS, 50 µL loading buffer (NuPAGETM LDS-sample buffer, ThermoFisher Scientific) were added and incubation at 70°C for 2×5 min with a brief vortex in between followed. Samples were stored at -80°C until preparation for mass spectrometric

proteomic analysis. As non-binding analogues of SyliteM and Sylite FSI**GV**SYPRRRRRRRRR, and (YSI**GV**SYPRpeg)$_2$KC, respectively, were used. These sequences contain a binding-abolishing AA swap, described earlier[4].

Mass spectrometric analysis of pulldowns

Alkylation of the eluate was achieved by reduction with 50 mM dithiothreitol for 10 min at 70°C and 650 rpm in a thermoshaker followed by addition of 2-iodoacetamide to a final concentration of 120 mM and incubation in the dark for 20 min. Afterwards, cold acetone was added in a 4.5:1 ratio and overnight incubation at -20°C followed. Then, the samples were centrifuged at 12,000 g for 20 min at 4°C. Pellets were washed with 4×1 mL of cold acetone with 5 min centrifugations at 12,000 g in between. Next, pellets were left to dry under ventilation for 10 min. The protein pellet was resuspended in 50 µL of 8 M urea in 100 mM ammonium bicarbonate (ABC) using a bioruptor (diagenode) with 3 cycles for 30 sec. Afterwards, 50 µL of 100 mM ABC were added, followed by addition of 0.25 µg endoproteinase LysC. After incubation for 2 hrs in a thermoshaker at 30°C and 900 rpm, 100 µL of 100 mM ABC and 0.25 µg trypsin were added. Following overnight incubation at 37°C, the samples were acidified using 20 µL of 10% trifluoracetic acid (TFA). Stage tips were prepared by insertion of three C18 disks into a pipette tip. Each Stage tip was pre-washed with 50 µL MeOH, followed by 50 µL of 60% ACN with 0.3% (v/v) FA, followed by equilibration with 2x50 µL of a 2% ACN solution with 0.3% (v/v) TFA. After sample loading, the tips were centrifuged for 10 min at 2,000 g and washed with 3×50 µL of 2% ACN with 0.3% (v/v) TFA. Elution was achieved using 2×50 µL of 60% ACN with 0.3% (v/v) FA, then the samples were lyophilized for storage until solubilization in 25 µL of 2% ACN with 0.1% (v/v) FA.

NanoLC-MS/MS analyses were performed on an Orbitrap Fusion (Thermo Scientific) equipped with a PicoView Ion Source (New Objective) and coupled to an EASY-nLC 1000 (Thermo Scientific). Peptides were loaded on capillary columns (PicoFrit, 30 cm×150 µm ID, New Objective) self-packed with ReproSil-Pur 120 C18-AQ, 1.9 µm (Dr. Daniel Maisch) and separated with a 60 min linear gradient from 3% to 30% acetonitrile and 0.1% FA at a flow rate of 500 nl/min.

Both MS and MS/MS scans were acquired in the Orbitrap analyzer with a resolution of 60,000 for MS scans and 7,500 for MS/MS scans. HCD fragmentation with 35% normalized collision energy was applied. A Top Speed data-dependent MS/MS method with a fixed cycle time of 3 sec was used. Dynamic exclusion was applied with a repeat count of 1 and an exclusion duration of 30 sec; singly charged precursors were excluded from selection. Minimum signal threshold for precursor selection was set to 50,000. Predictive AGC was

used with AGC a target value of 2×10^5 for MS scans and 5×10^4 for MS/MS scans. EASY-IC was used for internal calibration.

Raw MS data files were analyzed with MaxQuant version 1.6.2.2. Database search was performed with Andromeda, which is integrated in the utilized version of MaxQuant. The search was performed against the UniProt *mus musculus* reference proteome database (download date: 2020-08). Additionally, a database containing common contaminants was used. The search was performed with tryptic cleavage specificity with 3 allowed miscleavages. Protein identification was under control of the false-discovery rate (FDR; <1% FDR on protein and PSM level). In addition to MaxQuant default settings, the search was performed against following variable modifications: Protein N-terminal acetylation, Gln to pyro-Glu formation (N-term. Gln) and oxidation (Met). Carbamidomethyl (Cys) was set as fixed modification. Further data analysis was performed using R scripts developed in-house. LFQ intensities were used for protein quantitation. Proteins with less than two razor/unique peptides were removed. Missing LFQ intensities in the control samples were imputed with values close to the baseline. Data imputation was performed with values from a standard normal distribution with a mean of the 5% quantile of the combined log10-transformed LFQ intensities and a standard deviation of 0.1. For the identification of significantly enriched proteins, boxplot outliers were identified in intensity bins of at least 300 proteins. Log2 transformed protein ratios of sample versus control with values outside a 1.5x (significance 1) or 3x (significance 2) interquartile range (IQR), respectively, were considered as significantly enriched.

HEK293 and COS-7 cell cultures and transfection

HEK293 and COS-7 cells were cultured in DMEM (GIBCO), supplemented with GlutaMax and pyruvate (GIBCO), 10% fetal bovine serum (GIBCO) and 1% Penicillin/Streptomycin (Sigma) at 37°C and with 5% CO_2. Stable HEK293 cells expressing eGFP-gephyrin were grown with 0.4 mg/mL of the selective antibiotic G418.

The cells were plated on 0.15 mm thick 18 mm glass coverslips (HEK293 on coverslips that were coated with 35 µg/ml Poly-D-Lysine) in a 12-well plate and were transfected with 1 µg plasmid DNA per coverslip using PEI (Polyethylenimine). The transfection was performed at 60-80% confluence. Shortly before transfection the medium was changed to fresh DMEM. The DNA was added to 100 µl DMEM without additives and mixed, 4 µl fresh PEI (1 mg/ml) was added, mixed immediately and incubated for 20 min at RT. The transfection mix was pipetted drop-wise on cells while swirling, and incubated overnight. The medium was changed to fresh DMEM with 2% FBS after 12-24 hours, and on the following day the cells were fixed and used for staining.

The following constructs were used for transient transfection of HEK293 and COS-7 cells: eGFP-gephyrin P1[5] and eGFP - pEGFP-C2 were a gift from Prof. Matthias Kneussel (ZMNH, Germany); Venus-gephyrin[6] and pHluorin-tagged GlyR β-loop transmembrane protein[7] constructs (supplied by Dr. Christian G. Specht); gephyrin isoform constructs (Supplementary Table 1 and Appendix 1) provided by Prof. Eric Allemand (INSERM, France) and Dr. Fabrice Ango (INSERM, France).

Ethical approval statement

Approval for the experiments involving animals reported here was obtained from the relevant authorities. Experiments at the Institute of Clinical Neurobiology (Würzburg) were approved by the local veterinary authority (Veterinäramt der Stadt Würzburg, Germany) and government (Regierung von Unterfranken, Würzburg, Germany, FBVVL 568/200-324/13; TVA 55.2.2-2532-2-509/1067). Experiments in Paris are authorized by the Ministry of Agriculture and the Direction départementale des services vétérinaires de Paris (Ecole Normale Supérieure, animalerie des rongeurs, license B 75-05-20).

Culture and infection of primary neurons

Primary murine hippocampal neurons.

Primary murine hippocampal neurons were prepared from wildtype CD-1 mice (Jackson Laboratory) at embryonic day 17 (E17). Hippocampal neurons were grown in neurobasal medium (21103-049 Life Technologies, Massachusetts, USA) supplemented with 1% 200 mM L-Glutamine (25030-024 Life Technologies, Massachusetts, USA), 1% B27 (17504-044 Life Technologies, Massachusetts, USA). 50% of the medium was exchanged every 7 days in culture. 60,000 hippocampal neurons were seeded on 18 mm glass coverslips. Neurons were taken for experiments after three weeks in culture (day in vitro 21 = DIV21).

Primary murine cortical neurons.

All procedures involving animals were in compliance with the regulations of the French Ministry of Agriculture and the Direction départementale des services vétérinaires de Paris (Ecole Normale Supérieure, animalerie des rongeurs, license B 75-05-20). Primary murine cortical neurons were dissociated from wildtype C57BL/6J mice (Janvier, France) at embryonic day 17 (E17) and cultured on 18 mm glass coverslips in neurobasal medium containing B27, glutamax and penicillin/streptomycin (all from Gibco). Where required, neurons were infected at day in vitro 1 to 5 (DIV1-5) with lentivirus driving the expression of full-length gephyrin tagged at its N-terminus with mEos2[8]. Neurons were used for experiments after two to three weeks in culture (DIV15-21).

Cell fixation and immunocytochemistry

Neurons, COS-7 and HEK293 cells were fixed in 0.1 M sodium phosphate buffer pH7.4 containing 4% paraformaldehyde (EM grade, Polysciences) and 1% sucrose for 10-20 min at 37°C. After three rinses in phosphate buffered saline (PBS), the cells were permeabilized in PBS containing 0.1% Triton X-100 for 10 min at room temperature, rinsed again and blocked for 1 h in PBS with 3% bovine serum albumin (BSA). Primary and secondary antibodies were applied sequentially in blocking solution for 1 hour. The fluorescent probes were applied together with the primary antibody, unless otherwise mentioned.

Primary antibodies: mAb7a (147 011), mAb3B11 (147 111), Synaptic Systems.

Secondary antibodies were purchased from ThermoFisher: anti-mouse conjugated IgG with AlexaFluor (A) 647 (A-21235), A555 (A-21422), A488 (A-27023) or DyLight650 (84545).

Unless otherwise noted the SyliteM, Sylite and TMR2i were applied with 50 nM concentration, SyliteCy3 with 25nM, and both primary and secondary antibodies with 1:1000 concentration.

Wide field fluorescence microscopy

Unless otherwise stated the coverslips with samples were inserted in an imaging chamber (Ludin Chamber Type 1, Life Imaging Services) and imaged in PBS. The measurements were taken from distinct samples with a sample size ≥ 2, for each group. A series of images, used to generate the datapoints, were acquired from different regions of the sample, each region having a distinct group of cells.

Probe profiling in COS-7 cells

COS-7 expressing either eGFP or gephyrin-eGFP were labeled with either Sylite, SyliteM or TMR2i and imaged on an inverted Leica DMI6000B microscope with a 100x oil-immersion objective (NA 1.49) using a Leica DFC9000 GTC VSC-05760 sCMOS camera (16-bit, image pixel size: 130 nm). The following excitation and emission filters were chosen: excitation 470/40, emission 525/50 for gephyrin-eGFP and soluble eGFP; ex. 545/25, em. 605/70 for TMR2i (Tetramethylrhodamine); exc. 628/40, em. 692/40 for Sylites (Sulfo-Cyanine 5 – Cy5), 10 images were acquired at a frame rate (exposure time) of 100 ms and constant illumination intensity to ensure comparability. n≥8.

Gephyrin isoforms expressed in HEK293 cells

Images of transiently transfected HEK 293 cells were acquired with the above-described setup. The following excitation/emission filters were chosen: ex. 545/25, em. 605/70 for mScarlet, exc. 628/40, em. 692/40 for Sylites, and mAb7a/mAb3B11 with secondary DyLight650 Antibody. 10 images were acquired at a frame rate (exposure time) of 100 ms and constant illumination intensity to ensure comparability. n≥5.

SyliteCy3 profiling in HEK293 cells

HEK293 expressing either eGFP or gephyrin-eGFP were labeled with SyliteCy3 and imaged with the above-described setup. The following excitation/emission filters were chosen: excitation 470/40, emission 525/50 for gephyrin-eGFP and soluble eGFP; ex. 545/25, em. 605/70 for SyliteCy3. 10 images were acquired at a frame rate (exposure time) of 100 ms and constant illumination intensity to ensure comparability. n=10.

Neuron imaging

Primary murine hippocampal neurons were imaged using the above-described setup. n≥6.

Wide field imaging of primary murine cortical neurons was done on an inverted Nikon Eclipse Ti microscope with a 100x oil-immersion objective (NA 1.49) using an Andor iXon EMCCD camera (16-bit, image pixel size: 160 nm). The following excitation and emission filters were chosen: excitation 485/20, emission 525/30 for Alexa Fluor 488 and unconverted (green) mEos2; ex. 560/25, em. 607/36 for Cy3; exc. 650/13, em. 684/24 for Alexa Fluor 647 or Cy5 (Sylite). 10 images were acquired at a frame rate (exposure time) of 100 ms and at variable illumination intensity using a mercury lamp (Intensilight, Nikon) and neutral density filters to maximize the signal while avoiding saturation. All images in one channel were taken with constant settings to ensure comparability. n≥5.

2D image processing and analysis

Image processing and analysis were carried out using Fiji[9] (**F**iji **I**s **J**ust **I**mageJ) with JACoP[10] (**J**ust **A**nother **Co**localization **P**lugin) plugin for colocalization analysis. Macros and scripts (Appendix 2) were written by V.K.

mEos2-gephyrin single synapse segmentation and intensity recording was done with Icy[11] 2.0.3.0 using “Wavelet Spot Detector” function in a custom protocol written by V.K. (Appendix 3). mEos2-gephyrin synaptic puncta were segmented, average intensity of individual punctum was determined and compared to the average intensity of the corresponding punctum in the far-red spectrum for either mAb7a with a secondary A647 antibody or the staining of Sylites.

Dual-color dSTORM super-resolution imaging

Neurons were fixed at DIV20 and immuno-labelled with primary rabbit anti-RIM1/2 antibody (Synaptic Systems, No. 140203, 1:250 dilution) and mouse anti-gephyrin antibody (Synaptic Systems, mAb7a, No. 147011, 1:500; Synaptic Systems, mAb3B11, No. 147111, 1:1000) in blocking buffer for 2 hours. CF680-conjugated goat anti-rabbit secondary antibody (Biotium, No. 20818, one dye per IgG, 1:250) was co-applied with Alexa Fluor 647 (A647) – coupled donkey anti-mouse (1:500) or with Sylite at a final concentration of 500 nM for 2 h.

Coverslips were mounted in dSTORM buffer (Abbelight SMART-kit) on cavity slides (Heinz Herenz, No 1042001), sealed with twinsil (Picodent) and imaged. The measurements were taken from distinct samples with a sample size ≥ 3, for each group.

All three fluorophores (Cy5, A647, CF680) photo-switch under reducing and oxygen-free buffer conditions, making them suitable for dSTORM single molecule imaging[12], which enables the localization of the emitters with sub-diffraction localization precision. Thanks to their close spectral proximity, Cy5 or A647 were excited and acquired simultaneously with CF680 in the same dSTORM buffer (Abbelight SMART-Kit) using a 640 nm laser (Oxxius), and their respective signals discriminated after single molecule localization using a spectral demixing strategy[13]. To implement spectral demixing dSTORM of SyliteD – (Cy5 or gephyrin-A647) and RIM1/2-CF680 we used a dual-view Abbelight SAFe360, equipped with two Hamamatsu Fusion sCMOS cameras and mounted on an Olympus Ix83 inverted microscope with a 100X 1.5NA TIRF objective. The SAFe360 uses astigmatic PSF engineering to extract the axial position and achieves quasi-isotropic 3D localization precision, and a long-pass dichroic mirror to split fluorescence from single emitters on the two cameras.

Single molecule localization, drift correction, spectral demixing, data visualization and cluster analysis[14] (DBSCAN) were performed with Abbelight NEO software, using a neighborhood radius eps = 150 nm and minPts = 50 minimum neighbors for the antibody labelling. To compensate for the lower number of detections generated by Sylite we adjusted the DBSCAN parameters to eps = 200 nm and minPts = 10. To measure the distance between presynaptic RIM and the postsynaptic gephyrin cluster, the centers of mass of the segmented clusters were determined in each fluorescence channels The Euclidean distance representing the average distance between the two-point clouds was then calculated for each cluster.

Brain section preparation and staining

Wildtype C57BL/6J mice (Jackson Laboratory) were transcardially perfused via the left ventricle with ice-cold phosphate-buffer saline 1x (PBS1x) followed by ice-cold 4% paraformaldehyde (in PBS 1x). Brains were then removed, post-fixed in 4% PFA for 2 hours, cryoprotected in 30% sucrose/PBS for 48-72 hours and cut on a cryostat (Leica CM1950) in 50µm coronal slices. The immunohistochemistry was performed in free floating sections. Tissue sections were blocked with blocking solution (10% Donkey serum (Bio-rad) with 0.3% TritonX in PBS 1x) for 1 hour at RT, then fluorescent probes and primary antibodies were applied in blocking solution for 1 hour at RT, or 24/72 hours at 4°C. Then slices were washed 3 times with PBS and incubated with secondary antibody for 1 hour or 2 hours for

the 24/72 hours staining protocol at RT. When no antibodies were applied the slices were incubated for 1h at RT with the probes. Labelled sections were then incubated with DAPI (1:5000) for 5 min at RT and washed again with PBS. Lastly, the sections were mounted onto a gelatin-coated slides using mowiol as the mounting medium. Following primary antibodies were used: gephyrin mouse mAb7a 1:1000 and mouse mAb3B11 1:1000. The fluorophore-tagged secondary antibody used was Alexa 555 donkey anti-mouse (1:1000).

Wide field and confocal imaging of brain sections

Wide-field 20x microscopy of brain sections was done with a Zeiss Axio Imager 2 equipped with a Plan-Apochromat 20x/0.8 M27 objective. Images were taken with an Axiocam 506 and pixel size of 0,454 x 0,454 µm. For excitation of DAPI a wavelength of 353 nm with LED-Module 385nm (power 6.08%) and for the probes a wavelength of 650nm with LED-Module 630nm (power 22.50%) was used. Emission wavelength for DAPI was 465nm and for the probes 673nm. Image acquisition was set using Zeiss Tiles module.

Labeled samples were imaged on a Leica SP8 (Leica) confocal microscope equipped with an HC PL APO CS2 63.0×/1.40-NA oil UV objective. Images were taken using a 200-Hz resonant scanner, 12-bits, a voxel size of 58 × 58 × 170 nm^3, a pinhole of 108.7 µm (1 AU). For excitation violet 405 nm LASOS diode laser (power 1-2%), yellow-green 561 nm DPSS laser (power 1-4%), red 633 nm HeNe laser (power 1-2%) were used. Emission light was registered with Leica PMT detectors set to the following spectral ranges: 415-465 nm, gain 750-850V (DAPI channel); 575-620 nm, gain 800-950V (Sulfo-Cyanine 3 channel); 645-700 nm, gain 750-850V (Cy5 channel). Image acquisition was performed in sequential frame scan mode, with concurrent 405 nm and 633 nm excitation and acquisition in corresponding ranges, followed by 561 nm excitation with an acquisition in Sulfo-Cyanine 3 channel. Bleaching was compensated with a linear gain increase of 30-40 V for an hour. The measurements were taken from distinct samples with a sample size of 4 for each group.

Animals

Experimental subjects were 3- to 6-month-old offspring of C57BL/6 mice with mutated $Slc17a6^{tm1.1(flpo)Hze}$ (VgluT2-IRES2-FlpO) or $Slc6a13^{tm1.1Ncd}$ (Vgat2-2A-FlpO-D) genes (both lines from Jackson Laboratory) crossed with GlyT2-Cre mice initially provided by U. Zeilhofer (University Zurich). Before surgeries, mice were co-housed with littermates (2–5 per cage) in a temperature (22–24 °C) and humidity (40–60%) controlled environment, after surgery mice were individually housed in the same conditions. Mice were maintained with unrestricted access to food and water on a 12-h light/dark cycle, with tissue processed during the light phase. All mice were randomly assigned to experimental conditions, with approximately equal numbers of male and female mice.

Injection of viruses and anatomical tracing

Isoflurane (cp-pharma, induction 4%, maintenance 1-2%) in oxygen-enriched air was used to anaesthetize mice fixed in a stereotactic frame (Kopf Instruments 1900 series). Eyes were lubricated with an ophthalmic ointment, and body temperature was maintained at 32–37 °C with a heatpad Fur was shaved and the incision site was sterilized with Cutasept solution before beginning surgical procedures. Local injections of 200 µL ropivacainhydrochlorid (Naropin; 5mg/mL, AspenGlobal) was injected subcutaneously before opening of the scalp. Buprenophine (10 µL of 0.3 mg/mL, Bayer) was injected subcutaneously to ensure no pain. After completion of surgery, intraperitoneal injections of meloxicam were administered to alleviate pain (30µl of 5 mg/ml, Metacam; Boehringer). A craniotomy was made at the injection site with a round 0.5mm drill bit (David Kopf). A volume of 200-300nl virus solution was pressure-injected intracranially using calibrated glass pipets (5µl microcapillary tube; Sigma-Aldrich) pulled in Narishige PC-100 connected to a PDES-02X (npi electronics). Unilateral targeting of dmPAG and vlPAG was achieved with the following coordinates: dmPAG AP -3.20mm, ML +0.2mm, DV -2.00mm; vlPAG AP -4.80mm, ML +0.60mm, DV -3.00mm. The capillary was then manually slowly lowered until the desired injection depth was reached.

To discover glycinergic vlPAG intra-connectivity inputs we used a cre-dependent anterogradely transported AAV: AAV2/5-CAG- Floxed-SypGFP rev-WPRE.

To study glycinergic vlPAG projections to dmPAG, we combined a vlPAG injection of a cre-dependent, anterogradely transported AAV: AAV2/5/CAG-Floxed-Synaptophysin-10xMyc-.rev-WPRE with a dmPAG injection of a Flp-dependent AAV: AAV2/1/hsyn-Cre^{off}/Flp^{on}-EYFP.

Four weeks after injection, mice were sacrificed, transcardially perfused with 4% paraformaldehyde in PBS, brains were extracted and processed for histology as described above.

To evaluate the intra-connectivity of vlPAG GlyT2 neurons immunohistochemistry was performed in free floating sections as described above with the following primary antibody chicken anti-GFP 1:1000 (Abcam; cat nb: ab13970) and fluorophore-tagged secondary antibody Alexa 488 donkey anti-chicken 1:1000 (Jackson ImmunoResearch, 705-545-155)

To assess active local vlPAG inputs onto dmPAG glutamatergic vs GABAergic neurons, immunohistochemistry was also performed as described above with the following primary antibodies chicken anti-GFP 1:1000 (Abcam; cat nb: ab13970) and goat anti-Myc 1:500 (Abcam, ab9132). The fluorophore-tagged secondary antibody used was Alexa 488 donkey

anti-chicken 1:1000 (Jackson ImmunoResearch, 705-545-155) and Cy3 donkey anti-goat 1:1000 (Jackson ImmunoResearch, 703-165-147), respectively. Sylite was used to label post-synaptic active zones.

Synaptophysin-GFP image processing

PAG was sectioned in 50 μm thick slices, then each third slice was imaged sequentially to reconstruct PAG and trace the glycinergic pathways from vlPAG (8-9 slices per animal from 3 animals). Overview of the labelled PAG sections was obtained with Zeiss Axio Imager 2 microscope equipped with a Plan-Apochromat 20x/0.8 M27 objective. Images were taken with an Axiocam 506 and pixel size of 0,454 x 0,454 μm using LED-Module 475nm (power 11.12%) with an emission wavelength of 517nm. Image acquisition was set using Zeiss Tiles module. Zen connect was used to precisely perform z-stack in the region of interest. Z-stacks were then converted in 2D images using maximal orthogonal projection of Zen Blue Software. Quantification of the synaptic inputs from the ipsilateral was performed with machine learning based Zen Intelesis software. The software was trained by manual Synaptophysin-eGFP cluster segmentation. ROI's of the subregions of the PAG were performed using anatomical features of the region and the total number of synaptic inputs in that ROI was divided by the ROI area to obtain synaptic density. The following nomenclature for the subregions of the PAG nuclei was used: dorsal medial periaqueductal grey (dmPAG), dorsal lateral periaqueductal grey (dlPAG), lateral periaqueductal grey (lPAG), ventrolateral periaqueductal grey (vlPAG) (Supplementary Figure 7).

dmPAG image processing

Overview of the labeled samples was obtained with a Zeiss Axio Imager 2 microscope equipped with a Plan-Apochromat 5x/0.16 objective. Images were taken with an Axiocam 506 and pixel size of 2,724 x 2,724 μm. For excitation of DAPI a wavelength of 353 nm with LED-Module 385nm (power 3-6%), for the GFP a wavelength of 488nm with LED-Module 475nm (power 20-24%) and for the SynMyc a wavelength of 548nm with LED-Module 555nm (power 25-30%) was used. Emission wavelength for DAPI was 465nm, GFP was 517 and SynMyc 561. Image acquisition was set using Zeiss Tiles module.

Labeled samples were imaged on a Leica SP8 (Leica) confocal microscope equipped with an HC PL APO CS2 63.0×/1.40-NA oil UV objective. Images were taken using a 200-Hz resonant scanner, a voxel size of 59 × 59 × 170 nm, a pinhole of 108.7 μm (1 AU). For excitation blue 488 nm argon laser (power 1-3%), yellow-green 561 nm DPSS laser (power 1-3%), red 633 nm HeNe laser (power 1-3%) were used. Emission light was registered with Leica PMT detectors set to the following spectral ranges: 500-550 nm, gain 750-850V (green channel); 575-620 nm, gain 750-850V (red channel); 645-700 nm, gain 750-850V (far-red

channel). Image acquisition was performed in sequential frame scan mode, with concurrent 488 nm and 633 nm excitation and acquisition in corresponding ranges, followed by 561 nm excitation with an acquisition in the red channel. Bleaching was compensated with a linear gain increase of 30-40 V for an hour.

Subsequently, deconvolution using a computed PSF was applied (Huygens Professional package, Scientific Volume Imaging), and 3D volumetric representation, segmentation and modeling was done (Imaris, Oxford Instruments).

3D image processing

Confocal data was deconvoluted using a computed PSF (Huygens Professional package, Scientific Volume Imaging) with the following settings: Logarithmic vertical mapping function; manual background estimation, according to the intensity baseline; max. 40 iterations, background to noise ratio of 5, 0.05 quality threshold, optimized iteration mode, auto brick layout. 3D and volumetric representation, segmentation and modeling of the deconvoluted images were done in Imaris (Oxford Instruments).

The volumetric representation and segmentation were done with the following settings:

Wild-type hippocampal tissue

mAb3B11, mAb7a and Sylites (synapse segmentation): No smoothing, 10% intensity threshold, >800 Voxels volume threshold (~0.5 μm^3). DAPI (nuclei segmentation): 0.468 μm (8 pixels) smoothing, 5% intensity threshold, 10000 Voxels volume threshold.

Periaqueductal gray tissue

Gephyrin labeled with Sylite segmentation: No smoothing, 5% intensity threshold, >0.1 μm^3 volume threshold. Synaptophysin, labeled with goat anti-MYC antibody and donkey anti-goat Cy3 antibody segmentation: No smoothing, 5% intensity threshold, >0.1 μm^3 volume threshold. Neurons, labeled with chicken anti-eGFP antibody, and donkey anti-chicken Alexa 488 antibody segmentation: smoothing 0.117 μm (2 pixels), 2-10% intensity threshold, >2 μm^3 volume threshold.

Inhibitory synapse density in neurons was calculated by dividing total in-neuron gephyrin volume (voxels) by total neuron volume (voxels) in each tissue section of dmPAG. Gly+ synapse density: the total volume of gephyrin clusters (voxels) in proximity to synaptophysin (<1 μm distance) was divided by total neuron volume (voxels). Gly- synapse density: the total volume of gephyrin clusters (voxels), excluding the ones in proximity to synaptophysin, was divided by total neuron volume (voxels).

Supplementary Discussion

Probe development

Earlier reported dimeric gephyrin E domain binders[15,16] displayed an exceptional affinity in low nanomolar range but required two purification and synthesis cycles. To improve yields and facilitate the iterative selection we here use a double fmoc Lysin (**U**) building block to dimerize directly on resin. First, we designed and synthesized five tri-dioxaoctanoic acid dimerized peptides with different gephyrin binding sequence length and evaluated their binding to gephyrin E domain using isothermal titration calorimetry (Fig.S1A). The strongest binder had eight amino acid (AA) long binding sequence. Having determined the functionality of the Lysin-branched dimer we explored whether the linker type has an impact on the binding. To compare the different linker designs, we used the µSPOT[1] approach (a SPOT[17] and Celluspot[18] based peptide microarray synthesis method) for the comparison of eleven different dimeric linkers (Fig.S1B), all containing a core gephyrin binding sequence, and some containing previously described[4] affinity-enhancing mutations. Lastly, we tested how the length of the Gephyrin binding sequence influences the interaction, by truncating the eight amino acid sequence stepwise by one amino acid, to a minimum of three amino acids (Supplementary Table 1). The resulting 113 different dimeric binders and their monomeric counterparts were probed with gephyrin E domain in microarray format (Fig.S1C). We observed the most intense gephyrin signals for the binders having eight AAs (Fig.S1D,E), in line with our ITC findings. Notably, binders with a modified core binding motif had higher intensity than the wildtype motif. Next, we analyzed the impact of the different linkers. For the wildtype sequence we saw an increase in intensity for linkers 01, 05, 06, 07, 08, 09, 10, 11 (Supplementary Table 2) while the monomeric binder and other dimeric binders had significantly lower intensity than the highest intensity binder FSIVGSLP10U (Fig.S1E). These differences could not be resolved with the mutated, higher-affinity binders, possibly due to on-array saturation of the binders with protein, leading to near-equal intensity readouts. Taking this into account we selected the dioxaoctanoic acid linker for the following dimeric probes since it contributes several H-bridges, improves solubility and allows effective and economic synthesis.

Using microarray-based assays we have recently defined the sequence requirements for the binding of native gephyrin, by probing gephyrin binding peptide microarrays with mouse brain homogenate[1] (Fig.S1F). Guided by our findings on optimal dimer architecture we combined variants of this consensus binding motif and synthesized multiple different monovalent and dimeric peptides (Appendix 5) and conjugated them C- and N-terminally with sulfo-cyanine-5 (Cy5), Alexa Fluor 647 (A647) and rhodamine dyes (Supplementary Table 5). Cy5 and A647 are both suitable for dSTORM, with A647 being the brighter and

more stable fluorophore[19], while silicon rhodamine (SiR) is STED compatible and was shown to work in live cell assays[20]. In addition, hydrophobicity and overall charges was adjusted via N-terminal elongation of the core binding motif or addition of Arginines (Fig.S2A). Microscopy-based evaluation identified Sylite, a dimeric probe, and SyliteM, its monomeric counterpart as fluorescent probes with best correlation, brightness and probe overlap (Fig.S2) as well as high signal-to-background (Fig.S3). Notably, compared to the previously reported gephyrin probe, TMR2i[4], Sylites show 10- and 150-fold improved contrast (Fig.1c, SyliteM and Sylite, respectively) and are fully compatible with advanced super-resolution techniques like direct stochastic optical reconstruction microscopy (dSTORM, Fig.2D-F).

Supplementary Figures

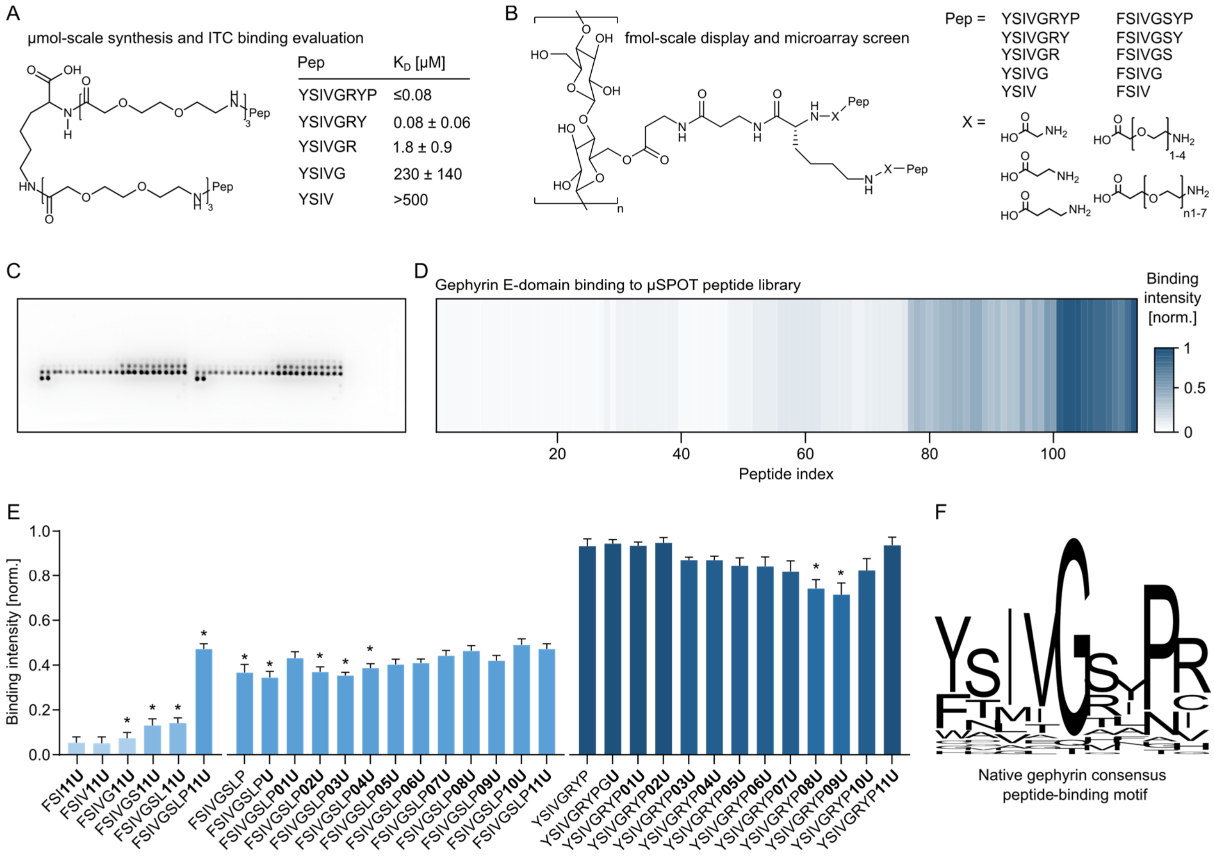

Figure S1. Optimization of probe sequence and multivalent architecture. A. Validation of dimer design strategy via ITC. Gephyrin E affinity of dimers having different-length binding sequences was determined. Minimal binder sequence length is ≥5 AAs. A ten-fold affinity increase is observed with the addition of, 7th and 8th amino acid to the binding sequence, the 8-mer binder having low-nanomolar affinity. **B.** µSPOT architecture of 113 monomeric and dimeric variants of gephyrin binding peptides. Peptides were based on ten binding sequences and eleven linker types (Supplementary Table 4). **C.** Representative example of the chemiluminescent gephyrin binding readout for the 113 monomeric and dimeric binders. Cellulose-conjugated peptides were printed on chips and incubated with 160pM of gephyrin E domain and subsequently detected with anti-gephyrin mAb3B11 and a secondary HRP conjugated antibody. Left and right are condition duplicates. **D**. Overview of the gephyrin binding intensities determined in microarray format. The intensities were internally normalized to the signal with highest intensity in the array and subsequently averaged and plotted for each peptide binder. **E.** The eight amino acid FSIVGSLP wild-type binding motif yields a higher luminescence signal than the shortened dimeric and monomeric analogues. Monomer and dimers (**01-11U**) based on the enhanced binder sequence YSIVGRYP have the highest intensity readout in the microarray. **08U** and **09U** dimers have lower readouts than the most intense binder YSIVGRYP**11U**. One-way ANNOVA followed up by Tukey test for multiple comparisons was performed. Mean±SEM, $P<0.05$. n=6. **F.** Native gephyrin consensus peptide-binding motif determined by screening and alignment of fine mapped gephyrin-binding sites[1].

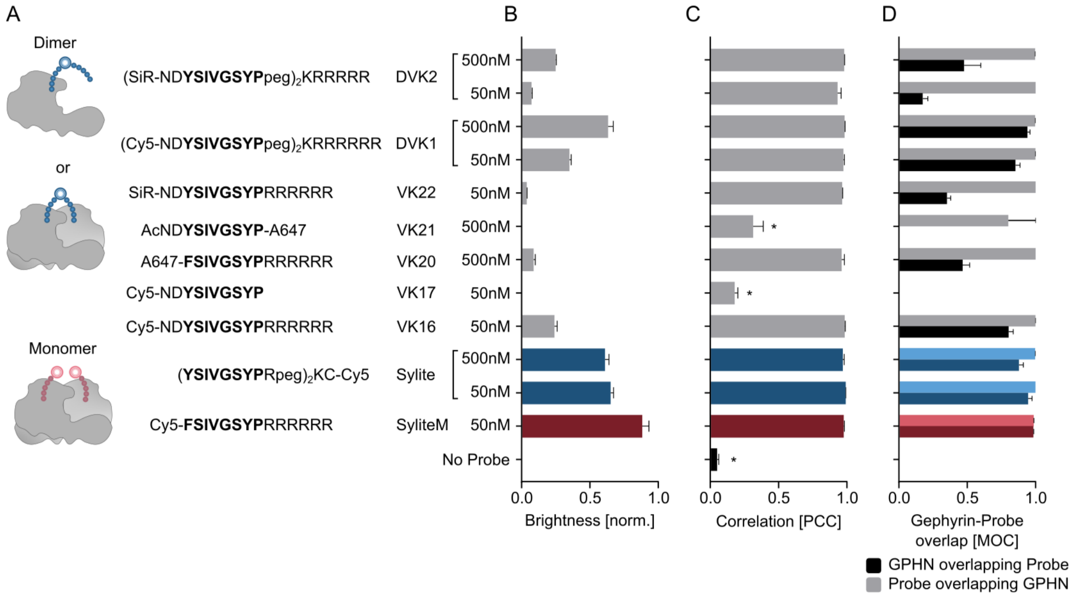

Figure S2. Microscopy-based probe evaluation. Systematic comparison of the imaging properties of the synthesized fluorescent gephyrin probes in COS7 cells expressing Venus-gephyrin. **A.** Binding modes of dimeric and monomeric probes together with sequences and fluorophores (Supplementary Table 3) of dimeric and monomeric probe variants (Appendix 5). The dimer can either bind one or two gephyrin molecules at once, while the monomer only one, always having stoichiometric 1:1 labeling. **B.** Comparison of the relative probe brightness. All probes are conjugated with far-red fluorophores, the average signal intensity coming from gephyrin clusters in far-red channel was divided by the corresponding average signal intensity of gephyrin clusters in green channel. Note that SyliteM is the brightest probe followed by Sylite. **C.** Pearson's Correlation Coefficients (PCC) of fluorescent probes and Venus-gephyrin. Next to the control only VK17 and VK21 show an incomplete correlation with gephyrin. **D**. Mander's overlap coefficients (MOC) - proportional coappearance of gephyrin and probe signals. When both values reach 1 it indicates an exclusive overlap of the two signals, meaning there is no over- or under- labeling of the target. Far-red signals coming from VK20, VK21, VK22 and DVK2 overlap almost completely with Venus-gephyrin, however Venus-gephyrin has only partial overlap with these probes, indicating under-labeling of the target. Significance was determined with ANOVA followed by Tukey's test for multiple comparisons. (*P<0.0001). Mean ± SEM.

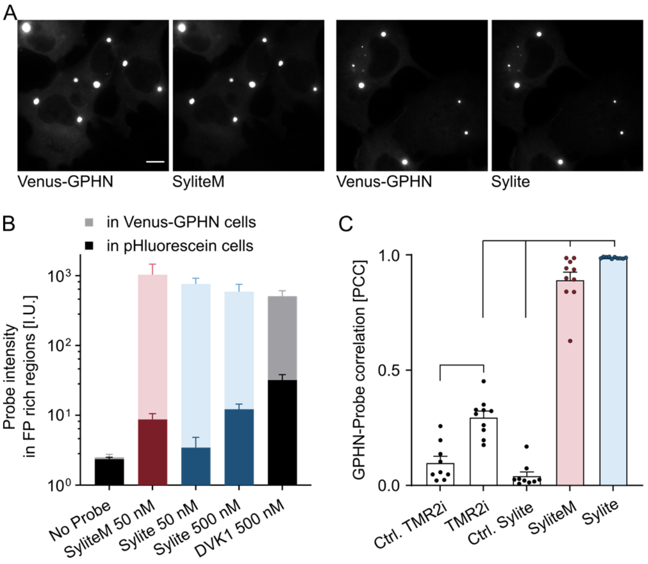

Figure S3. Sylites visualize gephyrin with high contrast. **A**. Left: fixed COS7 cells expressing Venus-gephyrin. Right: SyliteM and Sylite staining of the fixed cells. Scale bar 10 µm. **B**. Target and off target labeling of the probes having the highest relative brightness (Sylite, SyliteM and DVK1), overlap and correlation to gephyrin. COS7 cells expressing pHluorescein-tagged GlyR β-loop transmembrane protein were used as control. Regions with fluorescent protein (FP) were segmented and their corresponding intensity in far-red (probe) channel was measured. Log scale of average on target and off target labeling intensities for the three probes. Target-to-off target staining ratios of ~120, ~220, ~50 and ~15 were obtained for SyliteM (50 nM), Sylite (50 nM) and Sylite (500 nM), and DVK1 (500 nM), respectively. Mean ± SD. n≥5. **C.** Colocalization analysis of probes with gephyrin in fixed COS-7 cells. Sylite achieves a pearson's correlation coefficient (PCC) of ~1, indicating a complete correlation of the probe with gephyrin. Both Sylite and the monomer SyliteM have a significantly higher correlation to gephyrin than the earlier reported fluorescent probe TMR2i. Mean±SEM. Significance determined with ANOVA, using Dunnet's test for multiple comparisons, $P<0.05$.

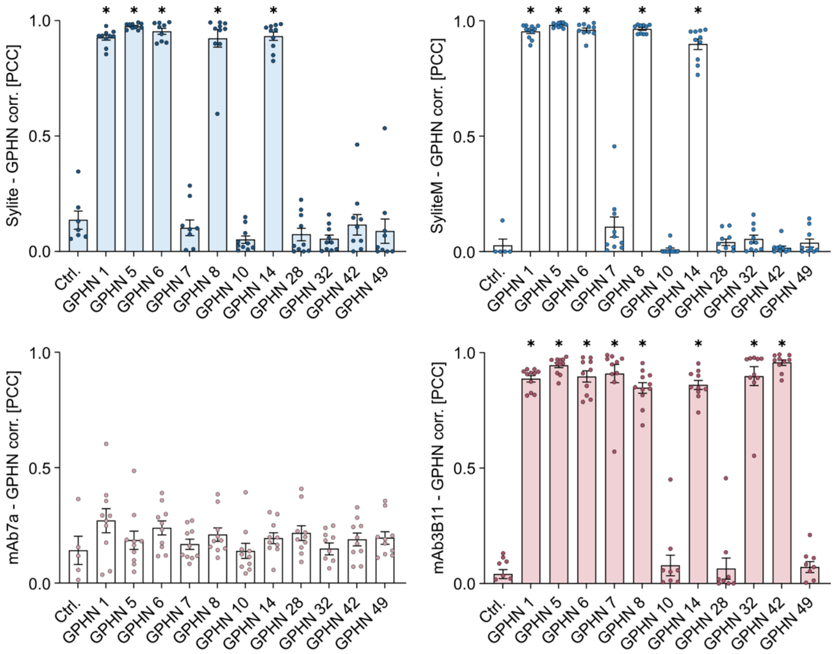

Figure S4. Sylites exclusively label gephyrin with receptor binding pocket. Colocalization analysis of probes with gephyrin (GPHN) isoforms in HEK293 cells. Control indicates cells expressing soluble mScarlet, that were stained with the antibodies or the fluorescent probes. Top: Sylite (left) and SyliteM (right) correlation to GPHN. Both the monomeric and the dimeric probes label 1,5,6,8 and 14 isoforms of gephyrin. Bottom: mAb7a (left) does not stain recombinant gephyrin in HEK293 cells; mAb3B11 stains all isoforms except 10,28,49. Mean±SEM. Significance (asterisks) determined with Brown-Forsythe and Welch ANOVA tests with Dunnet's T3 test for multiple comparisons to the control. $P<0.0001$.

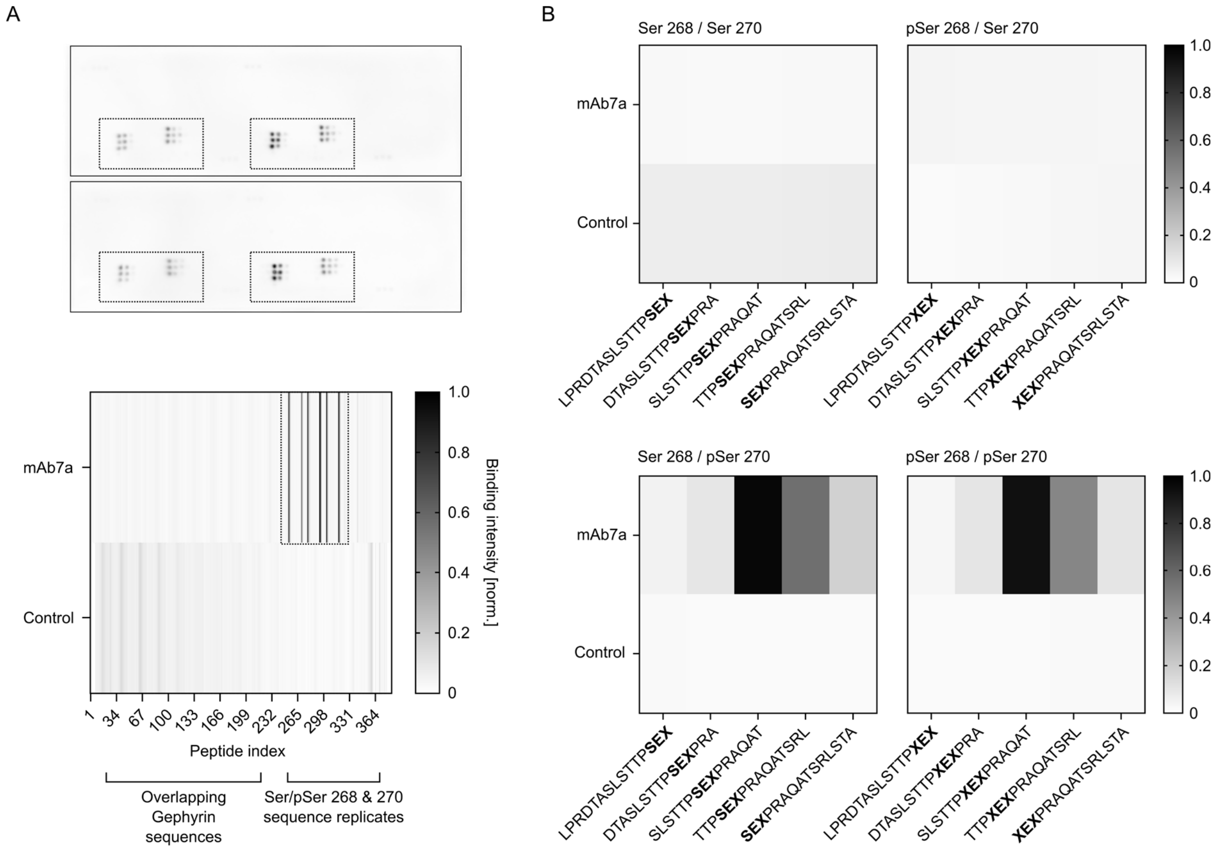

Figure S5. Gephyrin mAb7a explicitly binds a phosphorylated epitope. A. Representative examples (top) and averaged intensities as density blots (bottom) of mAb7a binding overlapping gephyrin fragments. The gephyrin (GPHN-1 isoform)[21] sequence was displayed in microarray format in the form of 15 AA peptides overlapping 12 AA with and without phosphorylations (Supplementary Table 5). mAb7a antibody binding was visualized with a secondary anti-mouse HRP conjugated antibody. Top panel: boxed - triplicates of phosphorylated peptide sequences. Bottom: a positional intensity readout, boxed is the region with phosphorylated sequence replicates. Intensities normalized to the highest intensity detected in the array. **B.** Averaged normalized intensity readout of the boxed region in A. X represents the phosphoserine. Chemiluminescent readout reports SLSTTPSEpSPRAQAT as the primary mAb7a epitope. Thus, indicating that phosphorylation of Serin 270 is necessary and sufficient for binding while phosphorylation of Ser 268 does not appear to affect binding.

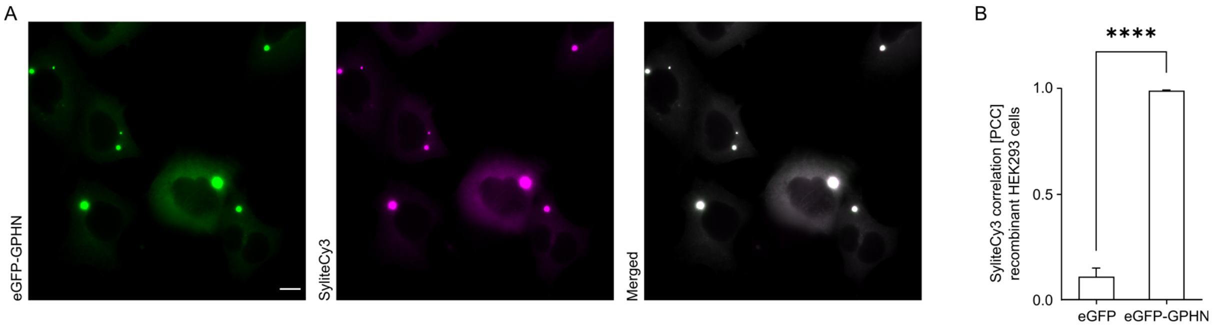

Figure S6. SyliteCy3 colocalizes with eGFP-gephyrin in mammalian cells. A. Fixed HEK293 cells expressing eGFP-gephyrin chimera (green) stained with SyliteCy3 (magenta), a full gephyrin-Sylite overlap (white) is observed. **B.** Colocalization analysis of HEK293 cells expressing eGFP-gephyrin or eGFP only stained by SyliteCy3. Pearson's correlation coefficient (PCC) of 0.985 (mean±SEM) indicates complete correlation of SyliteCy3 to eGFP-gephyrin signals. PCC of 0.107 indicates no correlation of SyliteCy3 to eGFP. n≥10 for each group. Significance determined with Mann-Whitney test, P<0.0001.

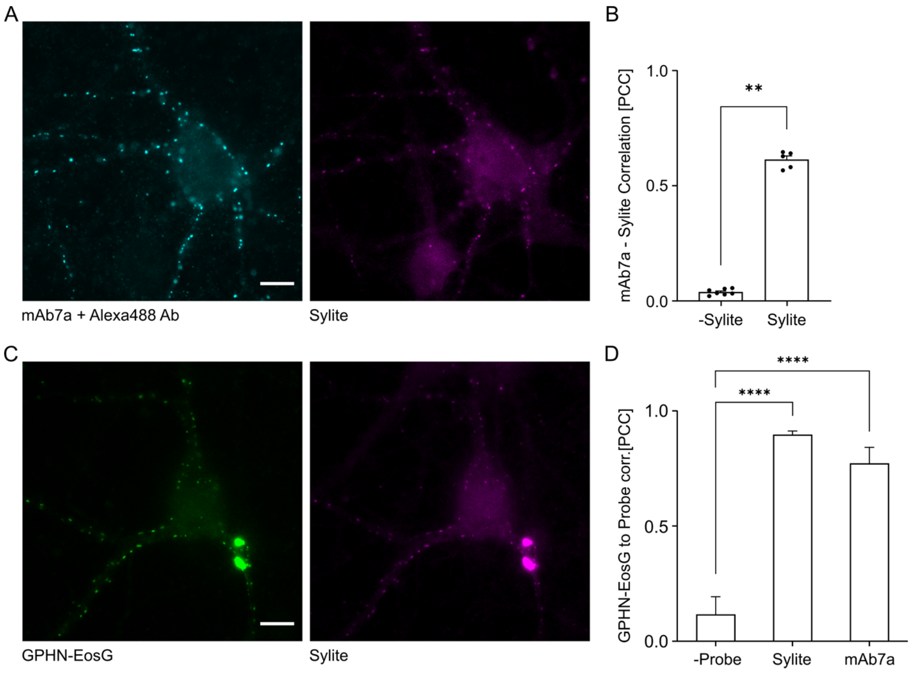

Figure S7. Sylite and mAb7a staining of wild-type and recombinant neurons. A. DIV21 cortical neurons were fixed and co-stained with Sylite (500 nM) and mAb7a. Similar pattern of antibody labeling and Sylite is observable. Scale bar 10 µm. **B.** Sylite staining correlates with the staining of mAb7a, but the degree of correlation is lower than with mAb3B11. These data are in-line with the narrow specificity of mAb7a, that labels only Ser270 phosphorylated neuronal gephyrin, therefore a lower correlation of mAb7a with Sylites is observed. On the "-Probe" group only a secondary mouse A488 antibody was applied. Mean ± SEM. Scale bar 10 µm. Significance determined with Mann-Whitney test. P<0.0025. **C.** Fixed cortical neurons expressing mEos2-gephyrin (green), with Sylite (500 nM) staining of the fixed sample (magenta). **D.** Pearson's correlation coefficients (PCC) of mEos2-gephyrin expressing neurons with the counterstain of SyliteM, Sylite or mAb7a. All probes show high correlation to the recombinant neurons. Mean±SEM. n≥5. Significance determined using one-way ANOVA with a follow up Tukey's test for multiple comparisons. P<0.0001.

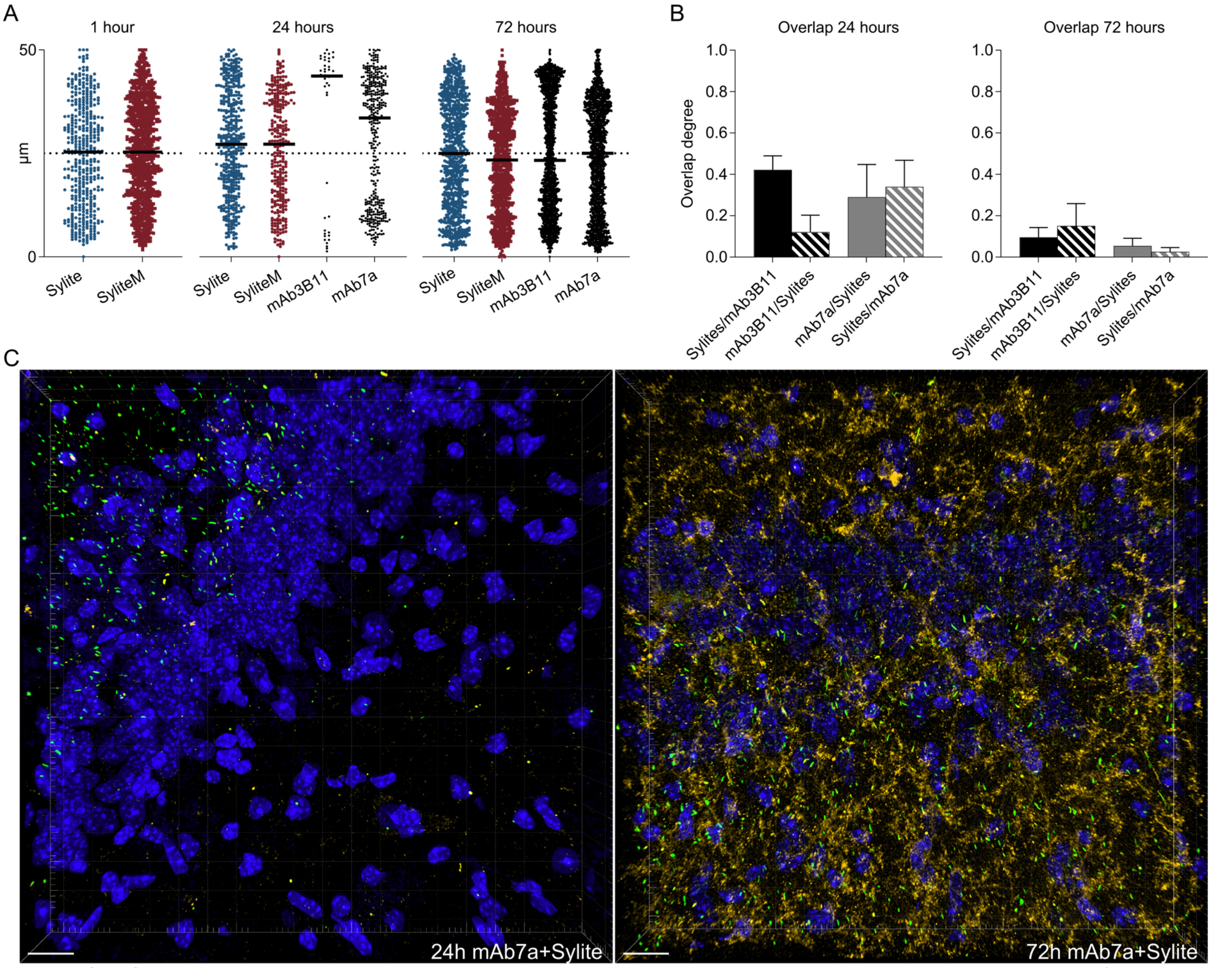

Figure S8. Sylites enable ultra-rapid synapse staining and visualization. A. In-tissue distribution of gephyrin probes. A complete and homogenous penetration of Sylites is observed already after 1 hour of staining. Antibody penetration remains inhomogeneous and incomplete after 24 and 72 hours. **B.** Sylite-antibody degree of overlap. 1 – full overlap, 0 – no overlap. After 24-hour staining of brain tissue about 40% of clusters detected by the antibodies are also Sylite-positive. The number of clusters detected by Sylites is higher than the number of clusters detected with mAb3B11, hence only ~10% of Sylite-positive clusters are co-labeled with mAb3B11. After 72-hour staining the maximum degree of overlap drops to ~15%, in line with the apparent increased unspecific staining of the antibodies. Mean ± SD. **C**. Sylites enable ultra-rapid synapse staining and visualization. Hippocampal section co-staining with Sylite and mAb7a. Green – Sylite, gold – mAb7a, blue – DAPI nuclear staining. Left – 24-hour staining. mAb7a and Sylite clusters partially overlap. mAb7a appears to have some unspecific connective tissue staining, Sylite synapse visualizations appear more consistent, a directional pattern of inhibitory synapse distribution can be observed. Right – 72-hour staining. Higher background staining with mAb7a is observed, the quality of Sylite labeling does not change. Scale bar 15 µm.

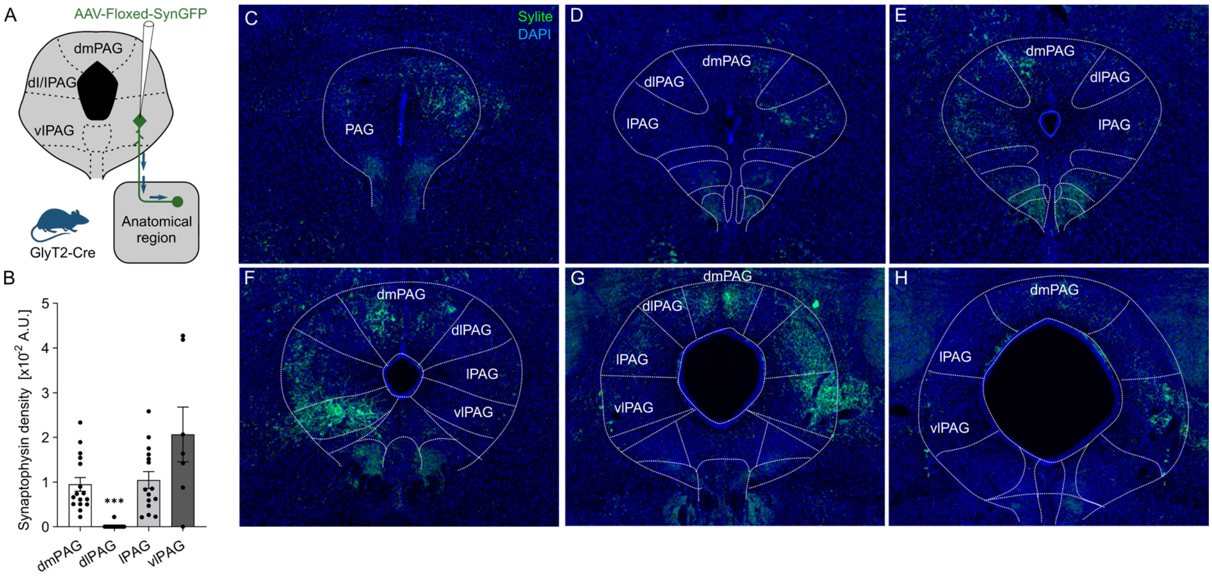

Figure S9. Glycinergic neurons from vlPAG project locally to lPAG and dmPAG. A. Anatomical tracing scheme of the injections and expression localizations in the periaqueductal gray. GlyT2-Cre recombinant mice were injected in posterior vlPAG with adeno-associated virus (AAV) carrying a plasmid with Cre recombinase-dependent Synaptophysin-eGFP chimera coding region. The synaptophysin-eGFP projections of the glycinergic neurons were traced and localized in specific anatomical regions of the PAG. **B.** Glycinergic projections from vlPAG are observable in lPAG and dmPAG, but not in dlPAG. Synaptophysin density: total synaptophysin in a specific anatomical region (μm^2) was divided by the total area of the region (μm^2) in each tissue section of dmPAG and plotted, mean±SEM, brain sections from three different animals were used. Significance determined with a Kruskal-Wallis test with a subsequent Dunn's multiple comparison test. P≤0.0001. **C-E.** Projection pattern of glycinergic vlPAG inputs to the different subregions of the PAG. Terminals of GlyT2+ vlPAG projection neurons were labelled by AAV-mediated expression of GFP fused to presynaptic marker synaptophysin from the more anterior (A) to the more posterior (F) part of the PAG.

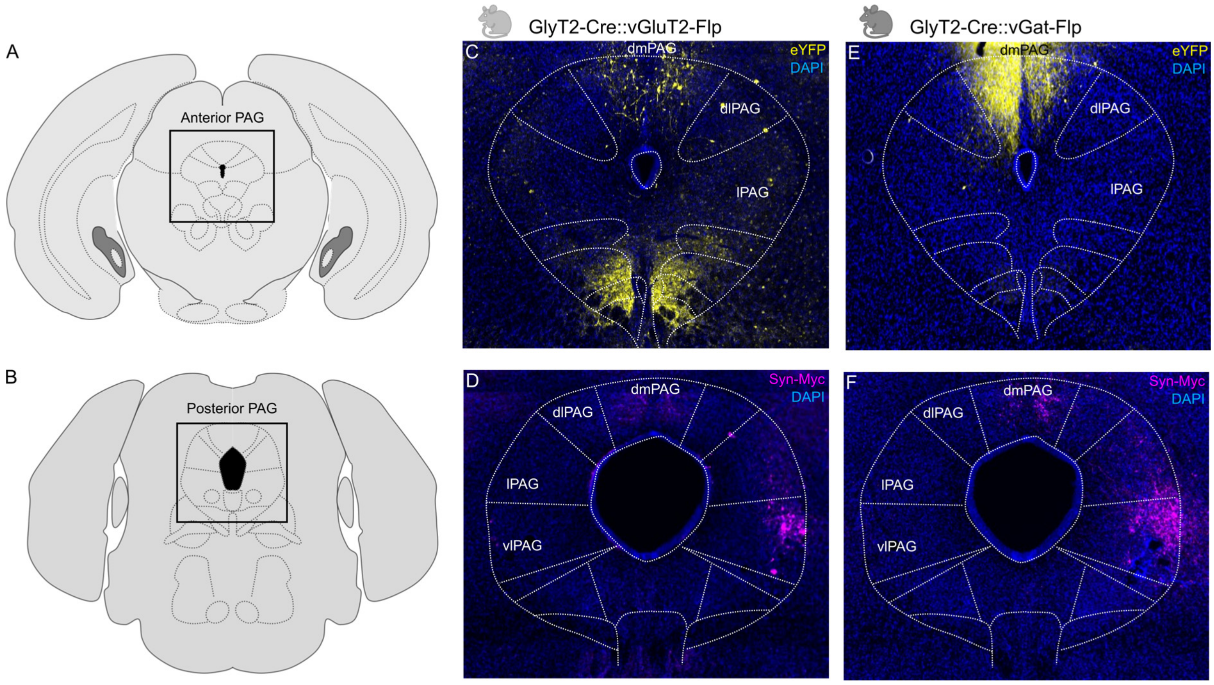

Figure S10. Injection sites of the viruses in the periaqueductal gray. A. Anatomical scheme of the injection site of the AAV2/1/hsyn-Cre^{off}/Flp^{on}-EYFP virus. **B.** Anatomical scheme of the injection site of the AAV2/5/CAG-Floxed-Synaptophysin-10xMyc-.rev-WPRE virus. **C.** Virally infected dmPAG glutamatergic neurons pattern. **D.** Injection site of the virus AAV2/5/CAG-Floxed-Synaptophysin-10xMyc-.rev-WPRE in a GlyT2-Cre::vGluT2-Flp. **E.** Distribution of virally infected GABAergic dmPAG neurons. **F.** Injection site of the virus AAV2/5/CAG-Floxed-Synaptophysin-10xMyc-.rev-WPRE in a GlyT2-Cre::vGat-Flp.

Supplementary Tables

Supplementary Table 1. Peptide microarray 1 sequences: Gephyrin binders

FSIG	FSIVG04K*	FSIVGS09K*	FSIVGSLP02G
FSIK*	FSIVG05K*	FSIVGS10K*	FSIVGSLP03G
FSI01K*	FSIVG06K*	FSIVGS11K*	FSIVGSLP04G
FSI02K*	FSIVG07K*	FSIVGSLG	FSIVGSLP05G
FSI03K*	FSIVG08K*	FSIVGSLK*	FSIVGSLP06G
FSI04K*	FSIVG09K*	FSIVGSL01K*	FSIVGSLP07G
FSI05K*	FSIVG10K*	FSIVGSL02K*	FSIVGSLP08G
FSI06K*	FSIVG11K*	FSIVGSL03K*	FSIVGSLP09G
FSI07K*	FSIVGG	FSIVGSL04K*	FSIVGSLP10G
FSI08K*	FSIVG01G	FSIVGSL05K*	FSIVGSLP11G
FSI09K*	FSIVG02G	FSIVGSL06K*	YSIVGRYPG
FSI10K*	FSIVG03G	FSIVGSL07K*	YSIVGRYPK*
FSI11K*	FSIVG04G	FSIVGSL08K*	YSIVGRYP01K*
FSIVG	FSIVG05G	FSIVGSL09K*	YSIVGRYP02K*
FSIVK*	FSIVG06G	FSIVGSL10K*	YSIVGRYP03K*
FSIV01K*	FSIVG07G	FSIVGSL11K*	YSIVGRYP04K*
FSIV02K*	FSIVG08G	FSIVGSLPK*	YSIVGRYP05K*
FSIV03K*	FSIVG09G	FSIVGSLP01K*	YSIVGRYP06K*
FSIV04K*	FSIVG10G	FSIVGSLP02K*	YSIVGRYP07K*
FSIV05K*	FSIVG11G	FSIVGSLP03K*	YSIVGRYP08K*
FSIV06K*	FSIVGSG	FSIVGSLP04K*	YSIVGRYP09K*
FSIV07K*	FSIVGSK*	FSIVGSLP05K*	YSIVGRYP10K*
FSIV08K*	FSIVGS01K*	FSIVGSLP06K*	YSIVGRYP11K*
FSIV09K*	FSIVGS02K*	FSIVGSLP07K*	
FSIV10K*	FSIVGS03K*	FSIVGSLP08K*	
FSIV11K*	FSIVGS04K*	FSIVGSLP09K*	
FSIVGK*	FSIVGS05K*	FSIVGSLP10K*	
FSIVG01K*	FSIVGS06K*	FSIVGSLP11K*	
FSIVG02K*	FSIVGS07K*	FSIVGSLPG	
FSIVG03K*	FSIVGS08K*	FSIVGSLP01G	

Supplementary Table 2. Linker building blocks

Abbreviation	Name
01	Glycine
02	β-Alanine
03	GABA
04	Oxapentanoic acid
05	Amino-PEG1 acid
06	Dioxaoctanoic acid
07	Amino-PEG2-acid
08	Amino-PEG3-Formicacid
09	Amino-PEG4-Formicacid
10	Amino-PEG5-acid
11	Amino-PEG7-acid

Supplementary Table 3. Fluorescent dyes

Abbreviation	Name	Structure
Cy5	Sulfo-Cyanine 5	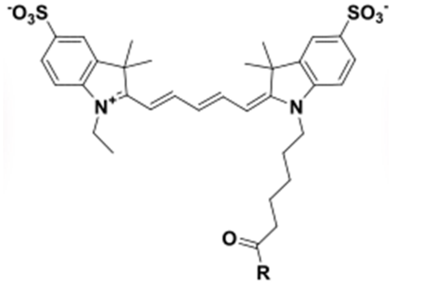
A647	Alexa647	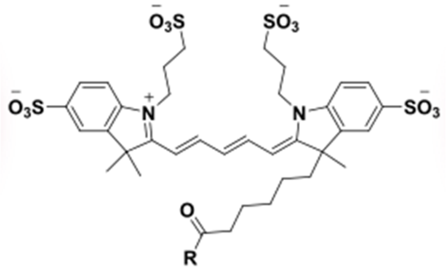
SiR	Silicone Rhodamine	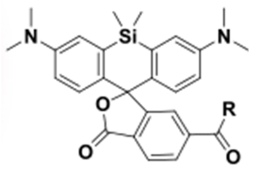
TMR	TAMRA (Carboxytetramethylrhodamine)	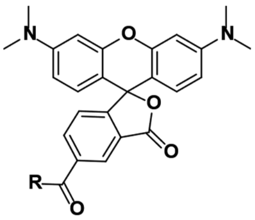

Supplementary Table 4. Sylite IP-MS analysis

	Sylite		
**Protein names**	**Unique Peptides**	**Log10 LFQ intensity**	**Log2 enrichment over control**
Tubulin alpha-4A chain	5	9.743956372	0.584659494
Serine/threonine-protein phosphatase 6 cat	5	8.743862388	1.165271614
Protein arginine N-methyltransferase 5	5	9.05422991	3.045182858
Histone deacetylase 6	7	9.017158598	1.011423821
Ras-related protein Rab-18	4	8.709643023	1.686198086
AP-3 complex subunit beta-2	10	8.881652798	1.667806449
Gephyrin;Molybdopterin adenylyltransferas	20	9.935602981	1.915168585
Ubiquitin-like modifier-activating enzyme 6	7	9.002900069	2.379527982
Tumor protein D52	6	9.466689716	0.829065762
CD166 antigen	5	8.747419577	1.502311769
Desmoplakin	11	8.933548024	1.880167352
26S proteasome non-ATPase regulatory sub	9	9.225696872	0.773465349
Dynamin-2	3	9.307175012	3.334602811
Cell adhesion molecule 3	6	9.283595183	2.995593321
Hypoxanthine-guanine phosphoribosyltrans	11	10.00449334	0.679598224
Sodium- and chloride-dependent GABA trar	4	8.679872866	1.578396554
Enoyl-CoA delta isomerase 1, mitochondrial	6	9.219741661	3.047408762
Protein S100-B	5	10.96715937	0.62275033
Peroxisomal multifunctional enzyme type 2;	4	8.868879446	2.108930696
Guanine nucleotide-binding protein G(I)/G(S	3	9.686975708	0.812033782
E3 ubiquitin-protein ligase RBX1;E3 ubiquitir	3	8.877670756	2.270320786
Eukaryotic translation initiation factor 4E	3	9.027920136	2.272716186
Ras-related protein Ral-A	3	8.782164352	1.543762875
ATP synthase subunit e, mitochondrial	3	8.903008549	2.354138905
26S proteasome non-ATPase regulatory sub	5	8.682560212	1.811575104
Haloacid dehalogenase-like hydrolase doma	9	9.679709461	0.619611003
Synaptic vesicle glycoprotein 2B	4	8.804091705	1.315536085
UPF0600 protein C5orf51 homolog	3	9.006508828	2.073758606
Endonuclease domain-containing 1 protein	5	9.038461196	2.33728129
Hydroxymethylglutaryl-CoA synthase, cytop	5	8.908988723	2.125199697
LYR motif-containing protein 4	4	8.795261141	2.438112278
tr|Q8R5L1|Q8R5L1_MOUSE Complement cc	4	9.557435014	0.676720944
UPF0696 protein C11orf68 homolog	4	8.792405699	1.445165084
Mitochondrial intermembrane space import	3	8.993502362	2.465625161
Protein S100;Protein S100-A1	3	9.652681385	0.700450053
GTP-binding protein Di-Ras1	3	8.766650752	1.71735903
Propionyl-CoA carboxylase alpha chain, mit	5	8.737931746	1.794738904
Trifunctional enzyme subunit beta, mitocho	4	8.739548612	1.361098945
Ubiquitin-like-conjugating enzyme ATG3	7	9.649325121	0.627128638
Cytochrome b-c1 complex subunit 7	4	8.984648809	2.063895649
Gamma-soluble NSF attachment protein	6	8.993039232	2.610455389
Arginine--tRNA ligase, cytoplasmic	3	8.649957615	1.476441609
ADP-ribosylation factor-like protein 2	3	8.729091861	1.08527878
Mitochondrial-processing peptidase subunit	4	8.777600795	1.571389005
Hyaluronan and proteoglycan link protein 1	5	9.022469613	2.755531644
Galactokinase	5	9.057399817	2.428822808
Small acidic protein	4	9.070148736	2.812445209
EH domain-containing protein 1	5	8.749767225	1.886600732
Dynactin subunit 3	7	8.689734951	1.895263895

SyliteM			
**Protein names**	**Unique Peptides**	**Log10 LFQ intensity**	**Log2 enrichment over control**
Abl interactor 2	4	8.840237869	1.627386384
Nuclear ubiquitous casein and cyclin-depen	7	9.443122112	3.046228545
Receptor-type tyrosine-protein phosphatas	4	9.249687428	3.176006515
NFU1 iron-sulfur cluster scaffold homolog, r	5	9.283888938	3.08854551
Cysteine protease ATG4B	5	8.972128752	2.517726771
Endophilin-A3	7	9.147583582	1.952476758
Amyloid beta A4 protein;N-APP;Soluble APP	6	9.425322238	3.329284422
Ran-binding protein 3	9	8.992252986	2.022561274
Gephyrin;Molybdopterin adenylyltransferas	20	9.869988142	2.456814084
UPF0587 protein C1orf123 homolog	5	9.18110008	2.427189177
Protein DDI1 homolog 2	5	9.091666958	3.060621454
Protein Shroom2	7	8.85836874	2.138436695
Cytoplasmic dynein 1 intermediate chain 2	5	9.092299477	2.681451784
tr|A2RTH5|A2RTH5_MOUSE Leucine carbox	12	9.379831068	3.267400004
Protein-glutamate O-methyltransferase	3	9.119222887	2.076670806
Protein archease	6	9.204255678	2.945472274
Glycogen synthase kinase-3 alpha	7	8.904060635	1.646585718
Proline-rich transmembrane protein 2	4	9.196507792	2.605882308
Ephexin-1	9	9.067070856	2.762141717
Serine/threonine-protein kinase B-raf	7	9.026042721	2.478157015
Polyadenylate-binding protein-interacting p	8	8.936201984	2.129552827
Hsc70-interacting protein	13	10.23225896	1.253853898
Ribose-phosphate pyrophosphokinase 1;Rib	4	9.097187873	3.201202868
E3 ubiquitin-protein ligase ARIH1	6	9.046104787	2.546357422
Cell adhesion molecule 3	2	9.174554035	2.999962336
Ubiquitin-protein ligase E3A	9	8.912136997	2.470247476
Transcriptional activator protein Pur-beta	6	8.971164477	2.177525217
Importin subunit alpha-4	10	9.385445394	1.711882851
Acidic leucine-rich nuclear phosphoprotein	12	10.33459447	1.21896867
Guanidinoacetate N-methyltransferase	3	8.944255595	1.573382505
Guanylate cyclase soluble subunit beta-1	7	8.70808081	1.609104882
Phospholipid hydroperoxide glutathione pe	5	8.897868909	1.604865811
Target of Myb protein 1	7	9.14160653	2.715353062
Neurofilament light polypeptide	3	8.752547791	1.776759407
Neuroendocrine protein 7B2;N-terminal pep	4	9.148602655	2.886125195
Secretogranin-1;CCB peptide;PE-11	10	9.185060283	2.865366559
Methylmalonyl-CoA mutase, mitochondrial	5	9.010130277	2.531885435
Ubiquitin-like protein 4A	5	8.814786798	1.827951872
Vitamin D-binding protein	7	9.013258665	2.508293265
AP-1 complex subunit gamma-1	2	8.580331833	1.782811289
GDP-L-fucose synthase	3	8.906399696	1.806946983
Talin-1	10	8.825302758	2.588304338
Metallothionein-3	5	9.51771041	3.126959315
Ras-related protein Rab-5C	4	8.971864716	2.172112131
Ran GTPase-activating protein 1	5	8.917941161	1.790169097
Secretogranin-3	5	8.905239843	2.11038256
Stathmin	11	10.20696073	2.193589206
UV excision repair protein RAD23 homolog	4	8.908849459	1.721768167
Tyrosine-protein phosphatase non-receptor	6	9.007705114	2.631910804

Endoplasmic reticulum resident protein 29	4	8.940262408	1.728015066
Neurogranin;NEUG(55-78)	2	10.11126251	2.745650847
Eukaryotic translation initiation factor 4E	6	9.055493007	2.168945363
Na(+)/H(+) exchange regulatory cofactor NH	9	9.272352241	2.034139864
Neuroplastin	3	9.306746608	3.944703967
Hematological and neurological expressed 1	3	9.121165748	3.503906028
Cytochrome b-c1 complex subunit 6, mitoch	3	9.104998949	2.516299516
Heterogeneous nuclear ribonucleoprotein U	4	8.747761278	2.136095077
Nucleobindin-1	15	9.442495447	1.984893254
Sorting nexin-12	6	8.799478399	2.430535836
S-adenosylmethionine synthase isoform typ	12	9.789841493	1.861210732
Tubulin--tyrosine ligase-like protein 12	9	8.756095756	2.197543506
Ubiquitin-conjugating enzyme E2 Z	8	9.336419705	3.563773643
Eukaryotic translation initiation factor 3 sub	3	8.788430001	1.791708573
Protein prune homolog 2	4	9.021396057	2.352930017
Synergin gamma	6	8.767541734	1.906236676
Src substrate cortactin	10	9.211254068	1.926669615
GTPase HRas;GTPase HRas, N-terminally pro	4	9.093736785	2.472404486
Nucleophosmin	4	8.739287468	1.92875029
Transcription intermediary factor 1-beta	8	9.076713245	2.186517729
Endophilin-A2	4	8.998812755	2.482440144
Sorting nexin-6;Sorting nexin-6, N-terminally	8	8.970249219	2.119110525
tr|Q91V89|Q91V89_MOUSE Serine/threoni	5	8.948183042	1.651657358
Glutaredoxin-related protein 5, mitochondr	3	9.122215878	2.681899123
Heterogeneous nuclear ribonucleoprotein A	3	8.844042042	2.354830652
N-terminal EF-hand calcium-binding protein	7	9.422606253	1.617052957
Eukaryotic translation initiation factor 4B	8	8.687947893	1.537475183
Protein FAM49A	6	9.30992811	1.956827769
PITH domain-containing protein 1	6	8.951536646	2.525865936
DCN1-like protein 2;DCN1-like protein	6	8.726466391	1.576064566
WD repeat-containing protein 37	9	9.342521373	2.146190144
tr|Q8CFX3|Q8CFX3_MOUSE Protocadherin	4	9.027634966	2.135578403
Poly(ADP-ribose) glycohydrolase ARH3	5	9.054766218	2.263852602
5-oxoprolinase	4	9.087461966	2.80591609
Protein farnesyltransferase subunit beta	4	8.815537903	2.163154128
Sulfite oxidase, mitochondrial	6	9.001214325	2.203861284
COP9 signalosome complex subunit 8	3	9.012837225	2.125057521
Peroxisomal biogenesis factor 19	4	9.011824096	2.474097818
UPF0696 protein C11orf68 homolog	7	8.859660578	2.264658074
Cell cycle and apoptosis regulator protein 2	7	8.829400272	1.612870534
Phosphate carrier protein, mitochondrial	3	9.237342629	3.140988826
Immunity-related GTPase family Q protein	9	9.060848873	1.647359195
Inorganic pyrophosphatase 2, mitochondria	12	9.684674841	2.053000945
ATP-dependent RNA helicase DDX1	11	9.288092655	3.740157619
Protein BRICK1	4	9.119486836	2.452183731
Glycine cleavage system H protein, mitochor	3	9.596035999	1.77335315
Adenylate kinase isoenzyme 5	2	8.418152081	1.59905039
Homer protein homolog 3	5	9.403292145	3.557556818
Adrenodoxin-like protein, mitochondrial	3	8.850254029	2.015084
RWD domain-containing protein 1	6	8.957410924	2.157244497
Heat shock factor-binding protein 1	4	9.139028547	2.351928408

26S proteasome non-ATPase regulatory sub	5	8.716854395	1.967814782
Vacuolar protein sorting-associated protein	6	8.813854421	1.936384367
Gamma-soluble NSF attachment protein	7	9.301203679	3.419673434
COP9 signalosome complex subunit 7a	3	9.238196699	3.023611179
Parathymosin	7	10.45055701	3.159189989
Nucleolar protein 3	4	9.269582986	2.86813258
Synapse-associated protein 1	4	9.239524703	3.080585246
Charged multivesicular body protein 4b	9	9.161487791	3.328427049
Sorting nexin-5	11	9.384675923	3.372258826
N-acetyl-D-glucosamine kinase	6	9.258014363	3.05459226
Ethylmalonyl-CoA decarboxylase	5	8.797059695	1.879258466
ATP synthase subunit d, mitochondrial	7	9.513430698	4.508290006
Calsyntenin-1;Soluble Alc-alpha;CTF1-alpha	6	8.940700722	2.848112276
Protein SET	7	9.881994865	1.245237688
Nucleosome assembly protein 1-like 5	4	8.974760316	2.027583761
Cell cycle exit and neuronal differentiation p	7	9.290457564	2.606475804
Protein NDRG3	4	8.86119975	1.753399906
Prostaglandin E synthase 3	9	10.67739705	1.548659392
RAC-gamma serine/threonine-protein kinas	8	9.093071306	2.935456966
STIP1 homology and U box-containing prote	4	8.981088887	2.109410011
Adenylate kinase 4, mitochondrial	3	8.712338177	2.285924931
Protein kinase C and casein kinase substrate	8	9.124960451	3.220362229
EH domain-containing protein 1	5	8.810528222	2.20788491
SUMO-activating enzyme subunit 2	12	9.698387659	1.180774345
ADP-ribosylation factor GTPase-activating pr	6	9.017742664	2.219402408

Supplementary Table 5. Gephyrin isoform constructs

pLVX-hSyn-Flag-V5-mScarlett-IHRES-ZsGreen1 (version Eral)

pLVX-hSyn-Flag-V5-mScarlett-GPHN-1-IHRES-ZsGreen1 (version Eral)

pLVX-hSyn-Flag-V5-mScarlett-GPHN-5-IRES-ZsGreen1 (version Eral)

pLVX-hSyn-Flag-V5-mScarlett-GPHN-6-IRES-ZsGreen1 (version Eral)

pLVX-hSyn-Flag-V5-mScarlett-GPHN-7-IRES-ZsGreen1 (version Eral)

pLVX-hSyn-Flag-V5-mScarlett-GPHN-8-IRES-ZsGreen1 (version Eral)

pLVX-hSyn-Flag-V5-mScarlett-GPHN-10-IRES-ZsGreen1 (version Eral)

pLVX-hSyn-Flag-V5-mScarlett-GPHN-14-IRES-ZsGreen1 (version Eral)

pLVX-hSyn-Flag-V5-mScarlett-GPHN-28-IRES-ZsGreen1 (version Eral)

pLVX-hSyn-Flag-V5-mScarlett-GPHN-32-IRES-ZsGreen1 (version Eral)

pLVX-hSyn-Flag-V5-mScarlett-GPHN-42-IRES-ZsGreen1 (version Eral)

pLVX-hSyn-Flag-V5-mScarlett-GPHN-49-IRES-ZsGreen1 (version Eral)

Supplementary Table 6. Peptide microarray 2 sequences: Gephyrin overlapping fragments

MATEGMILTNHDHQI	SKENILRASHSAVDI	IGHDIKRGECVLAKG	TTPXEXPRAQATSRL	TTPXEXPRAQATSRL
EGMILTNHDHQIRVG	NILRASHSAVDITKV	DIKRGECVLAKGTHM	XEXPRAQATSRLSTA	XEXPRAQATSRLSTA
ILTNHDHQIRVGVLT	RASHSAVDITKVARR	RGECVLAKGTHMGPS	-	-
NHDHQIRVGVLTVSD	HSAVDITKVARRHRM	CVLAKGTHMGPSEIG	-	-
HQIRVGVLTVSDSCF	VDITKVARRHRMSPF	AKGTHMGPSEIGLLA	-	-
RVGVLTVSDSCFRNL	TKVARRHRMSPFPLT	THMGPSEIGLLATVG	LPRDTASLSTTPSEX	LPRDTASLSTTPSEX
VLTVSDSCFRNLAED	ARRHRMSPFPLTSMD	GPSEIGLLATVGVTE	DTASLSTTPSEXPRA	DTASLSTTPSEXPRA
VSDSCFRNLAEDRSG	HRMSPFPLTSMDKAF	EIGLLATVGVTEVEV	SLSTTPSEXPRAQAT	SLSTTPSEXPRAQAT
SCFRNLAEDRSGINL	SPFPLTSMDKAFITV	LLATVGVTEVEVNKF	TTPSEXPRAQATSRL	TTPSEXPRAQATSRL
RNLAEDRSGINLKDL	PLTSMDKAFITVLEM	TVGVTEVEVNKFPVV	SEXPRAQATSRLSTA	SEXPRAQATSRLSTA
AEDRSGINLKDLVQD	SMDKAFITVLEMTPV	VTEVEVNKFPVVAVM	-	-
RSGINLKDLVQDPSL	KAFITVLEMTPVLGT	VEVNKFPVVAVMSTG	-	-
INLKDLVQDPSLLGG	ITVLEMTPVLGTEII	NKFPVVAVMSTGNEL	-	-
KDLVQDPSLLGGTIS	LEMTPVLGTEIINYR	PVVAVMSTGNELLNP	LPRDTASLSTTPXES	LPRDTASLSTTPXES
VQDPSLLGGTISAYK	TPVLGTEIINYRDGM	AVMSTGNELLNPEDD	DTASLSTTPXESPRA	DTASLSTTPXESPRA
PSLLGGTISAYKIVP	LGTEIINYRDGMGRV	STGNELLNPEDDLLP	SLSTTPXESPRAQAT	SLSTTPXESPRAQAT
LGGTISAYKIVPDEI	EIINYRDGMGRVLAQ	NELLNPEDDLLPGKI	TTPXESPRAQATSRL	TTPXESPRAQATSRL
TISAYKIVPDEIEEI	NYRDGMGRVLAQDVY	LNPEDDLLPGKIRDS	XESPRAQATSRLSTA	XESPRAQATSRLSTA
AYKIVPDEIEEIKET	DGMGRVLAQDVYAKD	EDDLLPGKIRDSNRS	-	-
IVPDEIEEIKETLID	GRVLAQDVYAKDNLP	LLPGKIRDSNRSTLL	-	-
DEIEEIKETLIDWCD	LAQDVYAKDNLPPFP	GKIRDSNRSTLLATI	-	-
EEIKETLIDWCDEKE	DVYAKDNLPPFPASV	RDSNRSTLLATIQEH	LPRDTASLSTTPXEX	LPRDTASLSTTPXEX
KETLIDWCDEKELNL	AKDNLPPFPASVKDG	NRSTLLATIQEHGYP	DTASLSTTPXEXPRA	DTASLSTTPXEXPRA
LIDWCDEKELNLILT	NLPPFPASVKDGYAV	TLLATIQEHGYPTIN	SLSTTPXEXPRAQAT	SLSTTPXEXPRAQAT
WCDEKELNLILTTGG	PFPASVKDGYAVRAA	ATIQEHGYPTINLGI	TTPXEXPRAQATSRL	TTPXEXPRAQATSRL
EKELNLILTTGGTGF	ASVKDGYAVRAADGP	QEHGYPTINLGIVGD	XEXPRAQATSRLSTA	XEXPRAQATSRLSTA
LNLILTTGGTGFAPR	KDGYAVRAADGPGDR	GYPTINLGIVGDNPD	-	-
ILTTGGTGFAPRDVT	YAVRAADGPGDRFII	TINLGIVGDNPDDLL	-	-
TGGTGFAPRDVTPEA	RAADGPGDRFIIGES	LGIVGDNPDDLLNAL	-	-
TGFAPRDVTPEATKE	DGPGDRFIIGESQAG	VGDNPDDLLNALNEG	LPRDTASLSTTPSEX	LPRDTASLSTTPSEX
APRDVTPEATKEVIE	GDRFIIGESQAGEQP	NPDDLLNALNEGISR	DTASLSTTPSEXPRA	DTASLSTTPSEXPRA
DVTPEATKEVIEREA	FIIGESQAGEQPTQT	DLLNALNEGISRANV	SLSTTPSEXPRAQAT	SLSTTPSEXPRAQAT
PEATKEVIEREAPGM	GESQAGEQPTQTVMP	NALNEGISRANVIIT	TTPSEXPRAQATSRL	TTPSEXPRAQATSRL
TKEVIEREAPGMALA	QAGEQPTQTVMPGQV	NEGISRANVIITSGG	SEXPRAQATSRLSTA	SEXPRAQATSRLSTA
VIEREAPGMALAMLM	EQPTQTVMPGQVMRV	ISRANVIITSGGVSM	-	-
REAPGMALAMLMGSL	TQTVMPGQVMRVTTG	ANVIITSGGVSMGEK	-	-
PGMALAMLMGSLNVT	VMPGQVMRVTTGAPI	IITSGGVSMGEKDYL	-	-
ALAMLMGSLNVTPLG	GQVMRVTTGAPIPCG	SGGVSMGEKDYLKQV	LPRDTASLSTTPXES	LPRDTASLSTTPXES
MLMGSLNVTPLGMLS	MRVTTGAPIPCGADA	VSMGEKDYLKQVLDI	DTASLSTTPXESPRA	DTASLSTTPXESPRA
GSLNVTPLGMLSRPV	TTGAPIPCGADAVVQ	GEKDYLKQVLDIDLH	SLSTTPXESPRAQAT	SLSTTPXESPRAQAT
NVTPLGMLSRPVCGI	APIPCGADAVVQVED	DYLKQVLDIDLHAQI	TTPXESPRAQATSRL	TTPXESPRAQATSRL
PLGMLSRPVCGIRGK	PCGADAVVQVEDTEL	KQVLDIDLHAQIHFG	XESPRAQATSRLSTA	XESPRAQATSRLSTA
MLSRPVCGIRGKTLI	ADAVVQVEDTELIRE	LDIDLHAQIHFGRVF	-	-
RPVCGIRGKTLIINL	VVQVEDTELIRESDD	DLHAQIHFGRVFMKP	-	-
CGIRGKTLIINLPGS	VEDTELIRESDDGTE	AQIHFGRVFMKPGLP	-	-
RGKTLIINLPGSKKG	TELIRESDDGTEELE	HFGRVFMKPGLPTTF	LPRDTASLSTTPXEX	LPRDTASLSTTPXEX
TLIINLPGSKKGSQE	IRESDDGTEELEVRI	RVFMKPGLPTTFATL	DTASLSTTPXEXPRA	DTASLSTTPXEXPRA
INLPGSKKGSQECFQ	SDDGTEELEVRILVQ	MKPGLPTTFATLDID	SLSTTPXEXPRAQAT	SLSTTPXEXPRAQAT
PGSKKGSQECFQFIL	GTEELEVRILVQARP	GLPTTFATLDIDGVR	TTPXEXPRAQATSRL	TTPXEXPRAQATSRL
KKGSQECFQFILPAL	ELEVRILVQARPGQD	TTFATLDIDGVRKII	XEXPRAQATSRLSTA	XEXPRAQATSRLSTA
SQECFQFILPALPHA	VRILVQARPGQDIRP	ATLDIDGVRKIIFAL		
CFQFILPALPHAIDL	LVQARPGQDIRPIGH	DIDGVRKIIFALPGN		
FILPALPHAIDLLRD	ARPGQDIRPIGHDIK	GVRKIIFALPGNPVS		
PALPHAIDLLRDAIV	GQDIRPIGHDIKRGE	KIIFALPGNPVSAVV		
PHAIDLLRDAIVKVK	IRPIGHDIKRGECVL	FALPGNPVSAVVTCN		
IDLLRDAIVKVKEVH	IGHDIKRGECVLAKG	PGNPVSAVVTCNLFV		
LRDAIVKVKEVHDEL	DIKRGECVLAKGTHM	PVSAVVTCNLFVVPA		
AIVKVKEVHDELEDL	RGECVLAKGTHMGPS	AVVTCNLFVVPALRK		
KVKEVHDELEDLPSP	CVLAKGTHMGPSEIG	TCNLFVVPALRKMQG		
EVHDELEDLPSPPPP	AKGTHMGPSEIGLLA	LFVVPALRKMQGILD		
DELEDLPSPPPPLSP	THMGPSEIGLLATVG	VPALRKMQGILDPRP		
EDLPSPPPPLSPPPT	GPSEIGLLATVGVTE	LRKMQGILDPRPTII		
PSPPPPLSPPPTTSP	EIGLLATVGVTEVEV	MQGILDPRPTIIKAR		
PPPLSPPPTTSPHKQ	LLATVGVTEVEVNKF	ILDPRPTIIKARLSC		
LSPPPTTSPHKQTED	TVGVTEVEVNKFPVV	PRPTIIKARLSCDVK		
PPTTSPHKQTEDKGV	VTEVEVNKFPVVAVM	TIIKARLSCDVKLDP		
TSPHKQTEDKGVQCE	VEVNKFPVVAVMSTG	KARLSCDVKLDPRPE		
HKQTEDKGVQCEEEE	NKFPVVAVMSTGNEL	LSCDVKLDPRPEYHR		
TEDKGVQCEEEEEEK	PVVAVMSTGNELLNP	DVKLDPRPEYHRCIL		
KGVQCEEEEEEKKDS	AVMSTGNELLNPEDD	LDPRPEYHRCILTWH		
QCEEEEEEKKDSGVA	STGNELLNPEDDLLP	RPEYHRCILTWHHQE		
EEEEEKKDSGVASTE	NELLNPEDDLLPGKI	YHRCILTWHHQEPLP		
EEKKDSGVASTEDSS	LNPEDDLLPGKIRDS	CILTWHHQEPLPWAQ		

KDSGVASTEDSSSSH	EDDLLPGKIRDSNRS	TWHHQEPLPWAQSTG
GVASTEDSSSSHITA	LLPGKIRDSNRSTLL	HQEPLPWAQSTGNQM
STEDSSSSHITAAAL	GKIRDSNRSTLLATI	PLPWAQSTGNQMSSR
DSSSSHITAAALAAK	RDSNRSTLLATIQEH	WAQSTGNQMSSRLMS
SSHITAAALAAKIPD	NRSTLLATIQEHGYP	STGNQMSSRLMSMRS
ITAAALAAKIPDSII	TLLATIQEHGYPTIN	NQMSSRLMSMRSANG
AALAAKIPDSIISRG	ATIQEHGYPTINLGI	SSRLMSMRSANGLLM
AAKIPDSIISRGVQV	QEHGYPTINLGIVGD	LMSMRSANGLLMLPP
IPDSIISRGVQVLPR	GYPTINLGIVGDNPD	MRSANGLLMLPPKTE
SIISRGVQVLPRDTA	TINLGIVGDNPDDLL	ANGLLMLPPKTEQYV
SRGVQVLPRDTASLS	LGIVGDNPDDLLNAL	LLMLPPKTEQYVELH
VQVLPRDTASLSTTP	VGDNPDDLLNALNEG	LPPKTEQYVELHKGE
LPRDTASLSTTPSES	NPDDLLNALNEGISR	KTEQYVELHKGEVVD
DTASLSTTPSESPRA	DLLNALNEGISRANV	QYVELHKGEVVDVMV
SLSTTPSESPRAQAT	NALNEGISRANVIIT	ELHKGEVVDVMVIGRL
TTPSESPRAQATSRL	NEGISRANVIITSGG	-
SESPRAQATSRLSTA	ISRANVIITSGGVSM	-
PRAQATSRLSTASCP	ANVIITSGGVSMGEK	-
QATSRLSTASCPTPK	IITSGGVSMGEKDYL	LPRDTASLSTTPSEX
SRLSTASCPTPKVQS	SGGVSMGEKDYLKQV	DTASLSTTPSEXPRA
STASCPTPKVQSRCS	VSMGEKDYLKQVLDI	SLSTTPSEXPRAQAT
SCPTPKVQSRCSSKE	GEKDYLKQVLDIDLH	TTPSEXPRAQATSRL
TPKVQSRCSSKENIL	DYLKQVLDIDLHAQI	SEXPRAQATSRLSTA
VQSRCSSKENILRAS	KQVLDIDLHAQIHFG	-
RCSSKENILRASHSA	LDIDLHAQIHFGRVF	-
	DLHAQIHFGRVFMKP	-
	AQIHFGRVFMKPGLP	LPRDTASLSTTPXES
	HFGRVFMKPGLPTTF	DTASLSTTPXESPRA
	RVFMKPGLPTTFATL	SLSTTPXESPRAQAT
	MKPGLPTTFATLDID	TTPXESPRAQATSRL
	GLPTTFATLDIDGVR	XESPRAQATSRLSTA
	TTFATLDIDGVRKII	-
	ATLDIDGVRKIIFAL	-
	DIDGVRKIIFALPGN	-
	GVRKIIFALPGNPVS	LPRDTASLSTTPXEX
	KIIFALPGNPVSAVV	DTASLSTTPXEXPRA
	FALPGNPVSAVVTCN	SLSTTPXEXPRAQAT

Supplementary References

[1] C. Schulte, V. Khayenko, N. F. Nordblom, F. Tippel, V. Peck, A. J. Gupta, H. M. Maric, *iScience* **2020**, 101898.

[2] B. Raveh, N. London, O. Schueler-Furman, *Proteins Struct. Funct. Bioinforma.* **2010**, *78*, 2029–2040.

[3] C. A. Brautigam, H. Zhao, C. Vargas, S. Keller, P. Schuck, *Nat. Protoc.* **2016**, *11*, 882–894.

[4] H. M. Maric, T. J. Hausrat, F. Neubert, N. O. Dalby, S. Doose, M. Sauer, M. Kneussel, K. Strømgaard, *Nat. Chem. Biol.* **2017**, *13*, 153–160.

[5] S. K. Tyagarajan, H. Ghosh, G. E. Yévenes, S. Y. Imanishi, H. U. Zeilhofer, B. Gerrits, J. M. Fritschy, *J. Biol. Chem.* **2013**, *288*, 9634–9647.

[6] C. Hanus, M. V. Ehrensperger, A. Triller, *J. Neurosci.* **2006**, *26*, 4586–4595.

[7] C. G. Specht, N. Grünewald, O. Pascual, N. Rostgaard, G. Schwarz, A. Triller, *EMBO J.* **2011**, *30*, 3842–3853.

[8] A. Patrizio, M. Renner, R. Pizzarelli, A. Triller, C. G. Specht, *Sci. Rep.* **2017**, *7*, 1–11.

[9] J. Schindelin, I. Arganda-Carreras, E. Frise, V. Kaynig, M. Longair, T. Pietzsch, S. Preibisch, C. Rueden, S. Saalfeld, B. Schmid, J.-Y. Tinevez, D. J. White, V. Hartenstein, K. Eliceiri, P. Tomancak, A. Cardona, *Nat. Methods* **2012**, *9*, 676–682.

[10] S. Bolte, F. P. Cordelières, *J. Microsc.* **2006**, *224*, 213–232.

[11] F. De Chaumont, S. Dallongeville, N. Chenouard, N. Hervé, S. Pop, T. Provoost, V. Meas-Yedid, P. Pankajakshan, T. Lecomte, Y. Le Montagner, T. Lagache, A. Dufour, J. C. Olivo-Marin, *Nat. Methods* **2012**, *9*, 690–696.

[12] M. Heilemann, S. Van De Linde, M. Schüttpelz, R. Kasper, B. Seefeldt, A. Mukherjee, P. Tinnefeld, M. Sauer, *Angew. Chemie Int. Ed.* **2008**, *47*, 6172–6176.

[13] A. Lampe, V. Haucke, S. J. Sigrist, M. Heilemann, J. Schmoranzer, *Biol. Cell* **2012**, *104*, 229–237.

[14] M. Ester, H.-P. Kriegel, J. Sander, X. Xu, others, in *Kdd*, **1996**, pp. 226–231.

[15] H. M. Maric, V. B. Kasaragod, H. Schindelin, *ACS Chem. Biol.* **2014**, *9*, 2554–2562.

[16] H. M. Maric, V. B. Kasaragod, L. Haugaard-Kedström, T. J. Hausrat, M. Kneussel, H. Schindelin, K. Strømgaard, *Angew. Chemie Int. Ed.* **2014**, *54*, n/a-n/a.

[17] R. Frank, *Tetrahedron* **1992**, *48*, 9217–9232.

[18] A. Dikmans, U. Beutling, E. Schmeisser, S. Thiele, R. Frank, *QSAR Comb. Sci.* **2006**, *25*, 1069–1080.

[19] D. A. Helmerich, G. Beliu, S. S. Matikonda, M. J. Schnermann, M. Sauer, *Nat. Methods* **2021**, *18*, 253–257.

[20] G. Lukinavičius, L. Reymond, E. D'Este, A. Masharina, F. Göttfert, H. Ta, A. Güther, M. Fournier, S. Rizzo, H. Waldmann, C. Blaukopf, C. Sommer, D. W. Gerlich, H. D. Arndt, S. W. Hell, K. Johnsson, *Nat. Methods* **2014**, *11*, 731–733.

[21] P. Prior, B. Schmitt, G. Grenningloh, I. Pribilla, G. Multhaup, K. Beyreuther, Y. Maulet, P. Werner, D. Langosch, J. Kirsch, H. Betz, *Neuron* **1992**, *8*, 1161–1170.

Supplementary Notes

Supplemetary Method 1. Imaging-based screening

Wide field imaging of labelled cells was done on an inverted Nikon Eclipse Ti microscope with a 100x oil-immersion objective (NA 1.49) using an Andor iXon EMCCD camera (16-bit, image pixel size: 160 nm). The following excitation and emission filters were chosen: excitation 485/20, emission 525/30 for A 488 and unconverted (green) mEos2; ex. 560/25, em. 607/36 for Cy3; exc. 650/13, em. 684/24 for A 647 or Cy5 (Sylite). Generally, 10 images were acquired at a frame rate (exposure time) of 100 ms and at variable illumination intensity using a mercury lamp (Intensilight, Nikon) and neutral density filters to maximize the signal while avoiding saturation. All images in one channel were taken with constant settings to ensure comparability.

Supplemetary Method 2. Peptide microarray synthesis

µSPOT [1] peptide microarrays were synthesized using a Celluspot-based approach[18] using a MultiPep RSi robot (CEM GmbH) on in-house produced, acid labile, amino functionalized, cellulose membrane discs containing 9-fluorenylmethyloxycarbonyl-β-alanine (Fmoc-β-Ala) linkers (average loading: 130 nmol/disc – 4 mm diameter). Synthesis was initiated by Fmoc deprotection using 20% piperidine (pip) in dimethylformamide (DMF) followed by washing with DMF and ethanol (EtOH). Peptide chain elongation was achieved using a coupling solution consisting of preactivated AAs (0.5 M) with ethyl 2-cyano-2 (hydroxyimino) acetate (Oxyma, 1 M) and N,N′-diisopropylcarbodiimide (DIC, 1 M) in DMF (1:1:1, AA:Oxyma:DIC). Couplings were carried out ×3×30 min, followed by capping (4% acetic anhydride in DMF) and washes with DMF and EtOH. Synthesis was finalized by deprotection with 20% pip in DMF (2×4 µL/disc for 10 min each), followed by washing with DMF and EtOH. Dried discs were transferred to 96 deep-well blocks and treated, while shaking, with sidechain deprotection solution, consisting of 90% trifluoracetic acid (TFA), 2% dichloromethane (DCM), 5% H2O and 3% triisopropylsilane (TIPS) (150 µL/well) for 1.5 h at room temperature (RT). Afterwards, the deprotection solution was removed, and the discs were solubilized overnight at RT, while shaking, using a solvation mixture containing 88.5% TFA, 4% trifluoromethanesulfonic acid (TFMSA), 5% H2O and 2.5% TIPS (250 µL/well). The resulting peptide-cellulose conjugates (PCCs) were precipitated with ice-cold ether (0.7 mL/well) and spun down at 2000×g for 10 min at 4°C, followed by two additional washes of the formed pellet with ice-cold ether. The resulting pellets were dissolved in DMSO (250 µL/well) to give final stocks. PCC solutions were mixed 2:1 with saline-sodium citrate (SSC) buffer (150 mM NaCl, 15 mM trisodium citrate, pH 7.0) and transferred to a 384-well plate. For transfer of the PCC solutions to white coated CelluSpot blank slides (76×26 mm, Intavis

AG), a SlideSpotter (CEM GmbH) was used. After completion of the printing procedure, slides were left to dry overnight.

Supplemetary Method 3. Microarray-based probe development

The microarray contained 241×15AA long peptides representing a full positional scan of the gephyrin protein (GPHN-1 isoform) with a 12AA overlap between the peptides and additional 45 Ser 268/270 phosphorylated peptide versions (Supplementary Table 5). The microarray slides were blocked for 1 h. in 2% (w/v) skimmed milk powder (Carl Roth) phosphate-buffered saline (PBS) (137 mM NaCl, 2.7 mM KCl, 10 mM Na_2HPO_4, 1.8 mM KH_2PO_4, pH 7.4). After blocking, the slides were incubated for 30 min. with 1:2500 dilution of mAb7a. mAb7a was detected with a secondary 1:5000 diluted HRP-coupled Anti-mouse antibody (G-21040, Invitrogen). The antibodies were applied in blocking buffer for 30 min., with x3 PBS washes between the antibodies and after the application of the secondary antibody. Chemiluminescent readout was obtained ("High sensitivity" mode (highest resolution; 1536 x 1024), 1s exposure time) after application of 200 µl of SuperSignal™ West Femto Atto Ultimate Sensitivity Substrate (Thermo Fischer Scientific Inc., Waltham, U.S.; Lot: A38554) per slide using a ImageQuant™ LAS 4000 imaging system (GE Healthcare Inc., Chicago, U.S.). Binding intensities were acquired with FIJI using the "Microarray Profile" plugin (OptiNav). The error range and the relative standard deviation were defined by comparing the intensities of each peptide duplicate on the respective array.

Appendix 1. Vector maps

Gephyrin isoform vector map

Plasmid map

pLVX-hSyn-Flag-V5-mScarlett-GPHN-1-IRES-ZsGreen1 (version Eral)

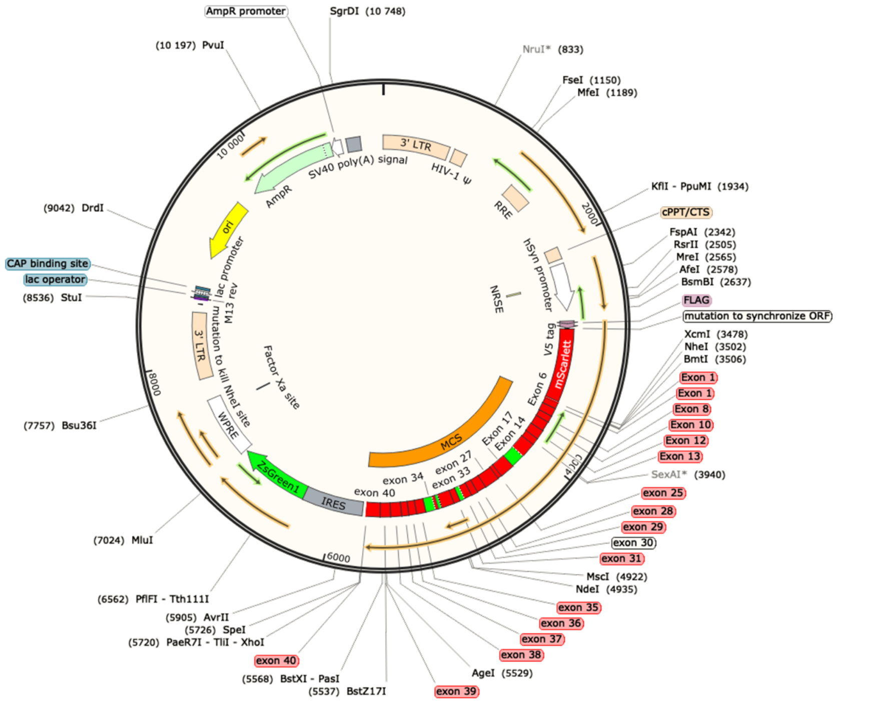

Floxed synaptophysin vector map

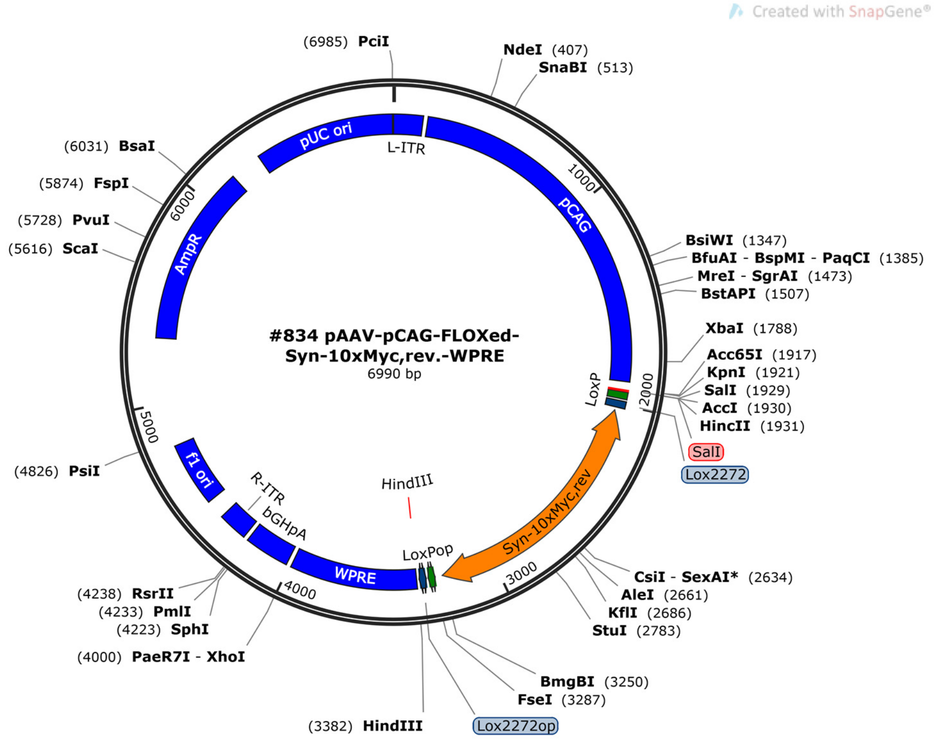

Coff/Fon eYFP vector map

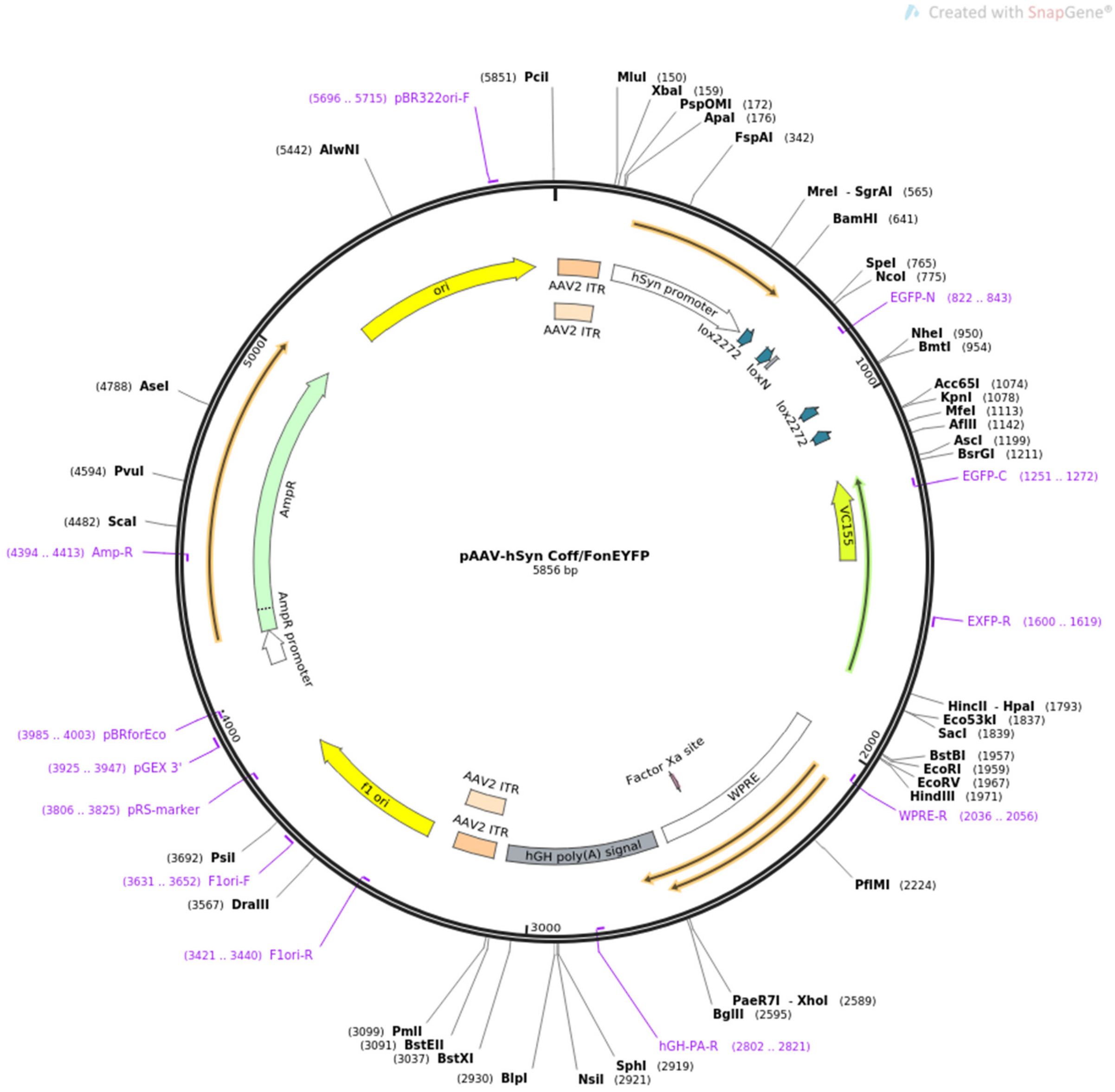

Appendix 2. Macro and script for Image analysis

Macro	Mode of operation	Utilization
I	• renaming the data format	• conversion of the proprietary image format to .tiff<br>• data import
II	• averages the image set to one image (noise reduction)<br>• sets greyscale representation of intensities<br>• sets the min and max values for the intensity display	• averaging of image series
III	• creates a binary mask and inverses the image<br>• measures the image background and subtracts the background	• subtraction of the image background
IV	• opens corresponding channel images iteratively<br>• automatically analyses the images in JACoP plugin and measures the colocalization	• colocalization analysis
V	• creates a binary mask corresponding to fluorescent protein location in the cell/object of interest<br>• tracks the binary mask in the target channel and measures the average intensity in ROI	• measurement of intensity in fluorescent protein rich regions in all color channels

Macro I:

```
path = File.openDialog("Select a File");
oldname = File.getName(path);
run("Bio-Formats Macro Extensions");
Ext.setId(path);
Ext.getCurrentFile(file);
Ext.getSeriesCount(seriesCount);
for (s=1; s<=seriesCount; s++) {
        run("Bio-Formats Importer", "open=&path autoscale color_mode=Default view=Hyperstack
stack_order=XYCZT series_"+s);
        oldtitle = getTitle();
        newtitle = replace(oldtitle, oldname + " - ", "");
        out_path = getDirectory("image") + newtitle;
        saveAs("tiff", out_path);
        run("Close All");
  }
```

Macro II:

```
dir1 = getDirectory("Select directory to average");
list = getFileList(dir1);
SaveDir = getDirectory("Select output directory");
for (g=0; g<list.length; g++) {
        open(dir1 + list[g]);
        originalImageName = getTitle();
        selectWindow(originalImageName);
        run("Z Project...", "projection=[Average Intensity]");
        selectWindow("AVG_" + originalImageName);
        run("Grays");
        //run("Brightness/Contrast...");
        setMinAndMax(150, 2500);
        save(SaveDir + originalImageName);
```

```
	close();
	close();
}
```

Macro III:

```
dir1 = getDirectory("Select image directory");
list = getFileList(dir1);
SaveDir = getDirectory("Select results directory");
	run("Set Measurements...", "area mean standard min integrated median
redirect=Nondecimal=3");
	for (g=0; g<list.length; g++) {
	ch1name = list[g];
	//Identifier = "-Probe";
	print(ch1name);
	open(dir1 + ch1name);
	//rename (g + Identifier);
	//creating a binary mask
	selectWindow(ch1name);
	run("Duplicate...", "title=Dup.tif");
	selectWindow("Dup.tif");
	run("Median...", "radius=5");
	run("Maximum...", "radius=5");
	run("Minimum...", "radius=5");
	setAutoThreshold("Default dark");
	setThreshold(250, 65535);

	setOption("BlackBackground", true);
	run("Convert to Mask");
	run("Make Inverse");
	roiManager("Add");
	RunningNumber = g + 1;
	roiManager("Save", SaveDir + ch1name + ".roi");
	selectWindow("Dup.tif");
	close();
//Mask done
selectWindow(ch1name);
roiManager("Select", 0);
	run("Measure");
	BG = getResult("Mean");
	print(BG);
	selectWindow(ch1name);
	run("Select None");
	run("Subtract...", "value=BG");
	setMinAndMax(0, 10000);
	save(SaveDir + ch1name);
	close();
	roiManager("reset");
}
selectWindow("Results");
saveAs("Measurements", SaveDir + "Results.tsv");
selectWindow("ROI Manager");
	run("Close");
}
```

Macro IV:

```
//Coloc measurement 2 folders, JACoP
function parseJACoP() { //Log to table function
	//Get the log window
	logdump = split(getInfo("log"), "\n");
	thrVals = false;
	imgA = -1;
```

```
	imgB= -1;
	Pc = -1;
	Oc = -1;
	OcThr = -1;
	k1Thr = -1;
	k1 = -1;
	k2Thr = -1;
	k2 = -1;
	thrA = -1;
	thrB = -1;
	M1 = -1;
	M2 = -1;
	M1Thr = -1;
	M2Thr = -1;
	a = -1;
	b = -1;
	R = -1;
	icq = -1;
	for (i=0; i<logdump.length; i++) {
		if (startsWith(logdump[i], "Image A"))
			imgA = substring(logdump[i], 9, lengthOf(logdump[i]));
		if (startsWith(logdump[i], "Image B"))
			imgB = substring(logdump[i], 9, lengthOf(logdump[i]));
		if (startsWith(logdump[i], "Pearson's Coefficient"))
			Pc = parseFloat(substring(logdump[i+1], 2, lengthOf(logdump[i+1])));

		if (startsWith(logdump[i], "Overlap Coefficient"))
			if (thrVals) {
				OcThr = parseFloat(substring(logdump[i+1], 2,
lengthOf(logdump[i+1])));
			} else {
				Oc = parseFloat(substring(logdump[i+1], 2, lengthOf(logdump[i+1])));
			}
		if (startsWith(logdump[i], "k1=")) {
			if (thrVals) {
				k1Thr = parseFloat(substring(logdump[i], 3, lengthOf(logdump[i])));
			} else {
				k1 = parseFloat(substring(logdump[i], 3, lengthOf(logdump[i])));
			}
		}
		if (startsWith(logdump[i], "k2=")) {
			if (thrVals) {
				k2Thr = parseFloat(substring(logdump[i], 3, lengthOf(logdump[i])));
			} else {
				k2 = parseFloat(substring(logdump[i], 3, lengthOf(logdump[i])));
			}
		}
		if (startsWith(logdump[i], "Using thresholds")) {
			thrA = parseFloat(substring(logdump[i], indexOf(logdump[i], "=")+1,
indexOf(logdump[i], "and")-1));
			thrB = parseFloat(substring(logdump[i], lastIndexOf(logdump[i], "=")+1,
lastIndexOf(logdump[i], ")")));
			thrVals = true;
		}
		if (startsWith(logdump[i], "Manders' Coefficients (original):")) {
			M1 = parseFloat(substring(logdump[i+1], 3, 8));
			M2 = parseFloat(substring(logdump[i+2], 3, 8));
		}
		if (startsWith(logdump[i], "Manders' Coefficients (using threshold")) {
			M1Thr = parseFloat(substring(logdump[i+1], 3, 8));
```

```
			M2Thr = parseFloat(substring(logdump[i+2], 3, 8));
		}
		if (startsWith(logdump[i], "Cytofluorogram's parameters:")) {
			a = parseFloat(substring(logdump[i+1], 3, 8));
			b = parseFloat(substring(logdump[i+2], 3, 8));
			R = parseFloat(substring(logdump[i+3], 25, 30));
		}
		if (startsWith(logdump[i], "ICQ"))
			icq = parseFloat(substring(logdump[i], 5, lengthOf(logdump[i])));
	}
	n=nResults;
	setResult("Pearson's", n, Pc);
	setResult("Overlap Coefficient (no threshold)", n, Oc);
	setResult("k1 (no threshold)", n, k1);
	setResult("k2 (no threshold)", n, k2);
	setResult("M1 (no threshold)", n, M1);
	setResult("M2 (no threshold)", n, M2);
	setResult("ThrA", n, thrA);
	setResult("ThrB", n, thrB);
	setResult("Correlation Coefficient", n, R);
	setResult("Overlap Coefficient", n, OcThr);
	setResult("k1", n, k1Thr);
	setResult("k2", n, k2Thr);
	setResult("M1", n, M1Thr);
	setResult("M2", n, M2Thr);
	setResult("Li's ICQ", n, icq);
	setResult("Cytofluorogram Slope", n, a);
	setResult("Cytofluorogram Intercept", n, b);
}
//Function end
dir1 = getDirectory("Select Green image directory");
list = getFileList(dir1);
dir2 = getDirectory("Select Far-Red image directory");
SaveDir = getDirectory("Select results directory");
for (g=0; g<list.length; g++) {
	ch1name = list[g];
	RefCh = "R";
	TargetCh = "FR";
	ch2name = replace(ch1name, RefCh, TargetCh)
	open(dir1 + ch1name);
	open(dir2 + ch2name);
	run("JACoP ", "imga=["+ch1name+"] imgb=["+ch2name+"] thra=400 thrb=160 pearson mm");
	parseJACoP();
	// Clear log window
	print("\\Clear");
	close(ch1name);
	close(ch2name);
}
saveAs("Measurements", SaveDir + "ColocData.tsv");
```

Macro V:

```
dir1 = getDirectory("Select reference image directory");
list = getFileList(dir1);
dir2 = getDirectory("Select target image directory");
SaveDir = getDirectory("Select output directory");
for (g=0; g<list.length; g++) {
	RunningNumber = g + 1;
	ch1name = list[g];
	RefCh = "R";
	TargetCh = "FR";
```

```
        ch2name = replace(ch1name, RefCh, TargetCh);
        print(ch1name);
        open(dir1 + ch1name);
//creating a Mask from a reference channel ROI [R]
        selectWindow(ch1name);
        run("Duplicate...", "title=Dup.tif");
        selectWindow("Dup.tif");
        run("Median...", "radius=5");
        run("Maximum...", "radius=5");
        run("Minimum...", "radius=5");
        setAutoThreshold("Default dark");
        setThreshold(600, 65535);
        setOption("BlackBackground", true);
        run("Convert to Mask");
        run("Create Selection");
        roiManager("Add");
        roiManager("Save", SaveDir + RunningNumber + ".roi");
        selectWindow("Dup.tif");
        close();
//Mask done
//creating 2nd Mask
        selectWindow(ch1name);
        run("Duplicate...", "title=Dup.tif");
        selectWindow("Dup.tif");
        setAutoThreshold("Default dark");
        setThreshold(4000, 65535);
        setOption("BlackBackground", true);
        run("Convert to Mask");
        run("Create Selection");
        roiManager("Add");
        roiManager("Select", 1);
        roiManager("Save", SaveDir + RunningNumber + "-punctae" + ".roi");
        selectWindow("Dup.tif");
        close();
//Mask done
//3rd mask for mScarlet-gephyrin controls
        selectWindow(ch1name);
        run("Duplicate...", "title=Dup.tif");
        selectWindow("Dup.tif");
        run("Median...", "radius=5");
        run("Maximum...", "radius=5");
        run("Minimum...", "radius=5");
        setAutoThreshold("Default dark");
        setThreshold(1000, 65535);
        setOption("BlackBackground", true);
        run("Convert to Mask");
        run("Create Selection");
        roiManager("Add");
        roiManager("Select", 2);
        roiManager("Save", SaveDir + RunningNumber + "-foreGFP" + ".roi");
        selectWindow("Dup.tif");
        close();
        //Mask done
        selectWindow(ch1name);
        roiManager("Select", 0);
        run("Measure");
        roiManager("Select", 1);
        run("Measure");
        roiManager("Select", newArray(0,1));
        roiManager("XOR");
```

```
		run("Measure");
		roiManager("Select", 2);
		run("Measure");
		close();
		open(dir2 + ch2name);
		roiManager("Select", 0);
		run("Measure");
		roiManager("Select", 1);
		run("Measure");
		roiManager("Select", newArray(0,1));
		roiManager("XOR");
		run("Measure");
		roiManager("Select", 2);
		run("Measure");
		close();
		roiManager("reset");
}
selectWindow("Results");
saveAs("Measurements", SaveDir + "Results.tsv");
run("Close");
selectWindow("ROI Manager");
run("Close");
```

Appendix 3. Icy 2.0.3.0 protocol for single synapse segmentation and intensity recording

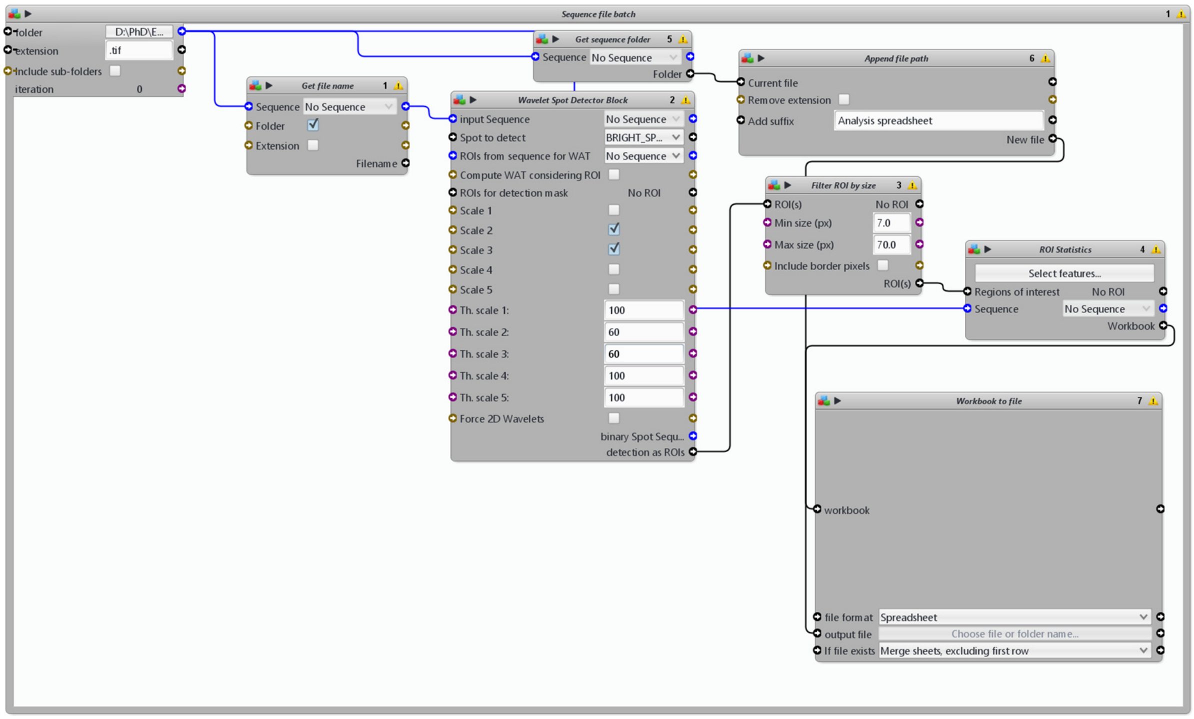

Appendix 4. Spectral profiles of Sylites

Absorption

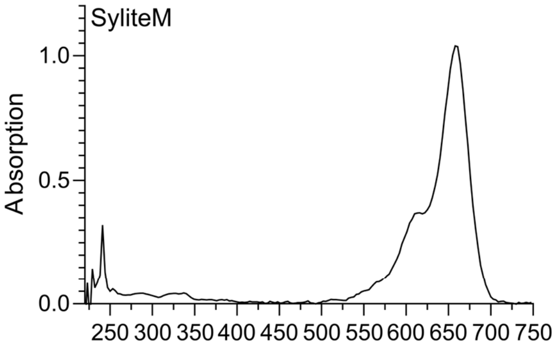

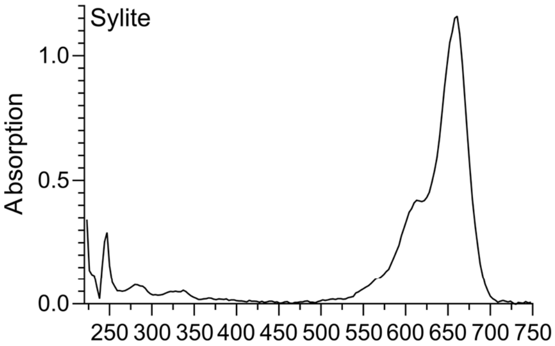

Excitation

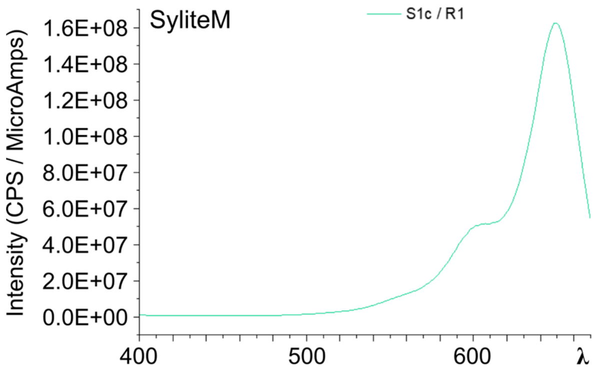

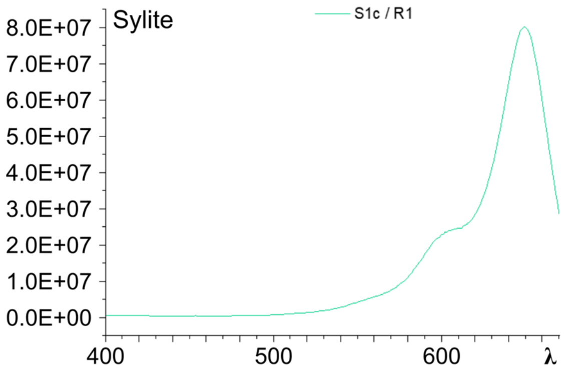

Emission

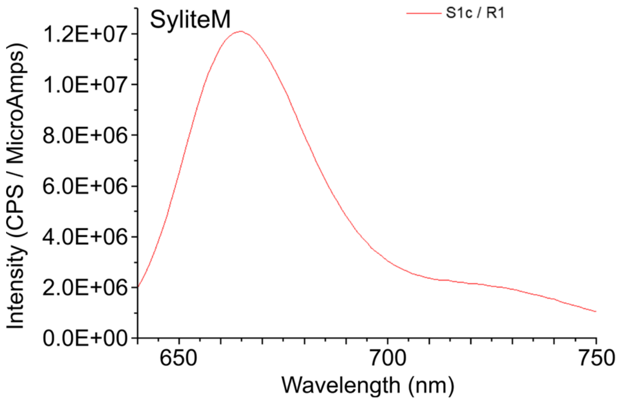

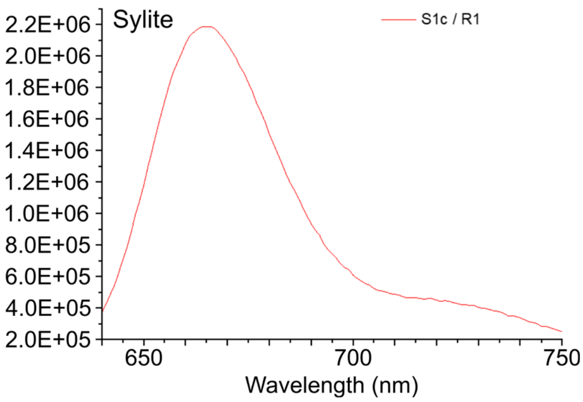

Appendix 5. Chromatographic and mass spectrometric probe validation

SyliteM

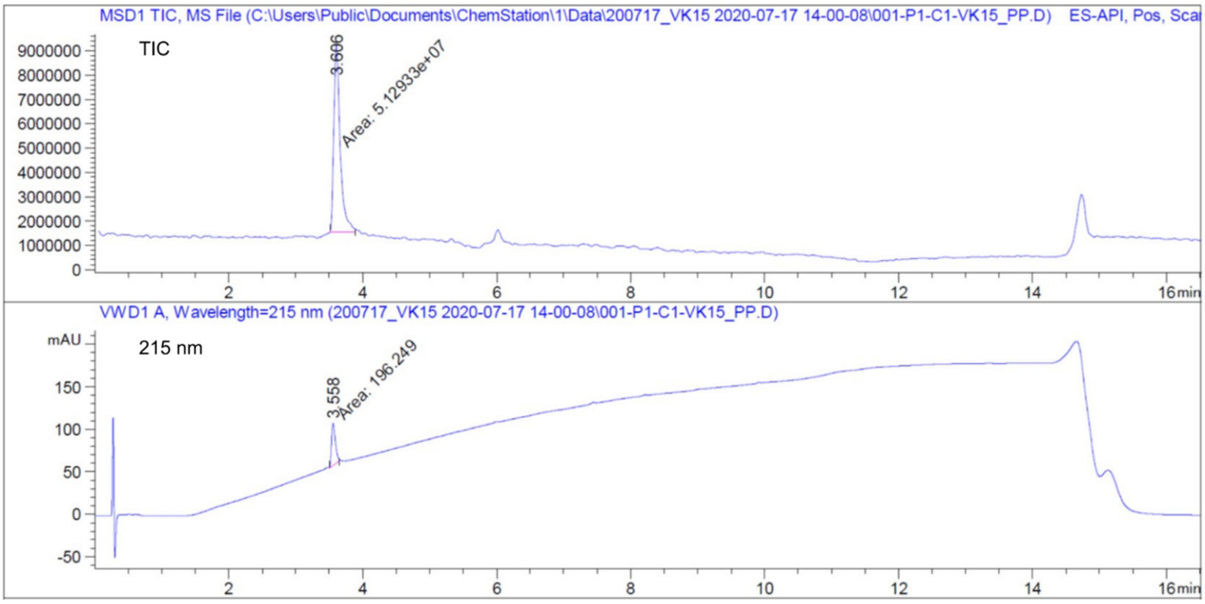

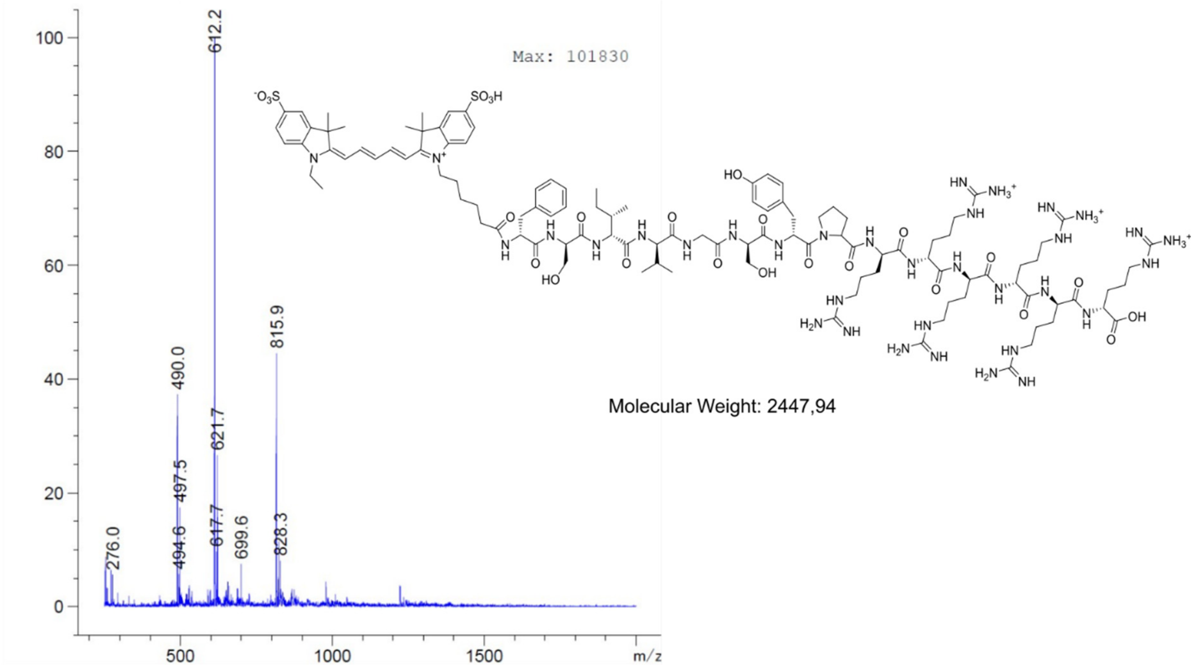

Sylite

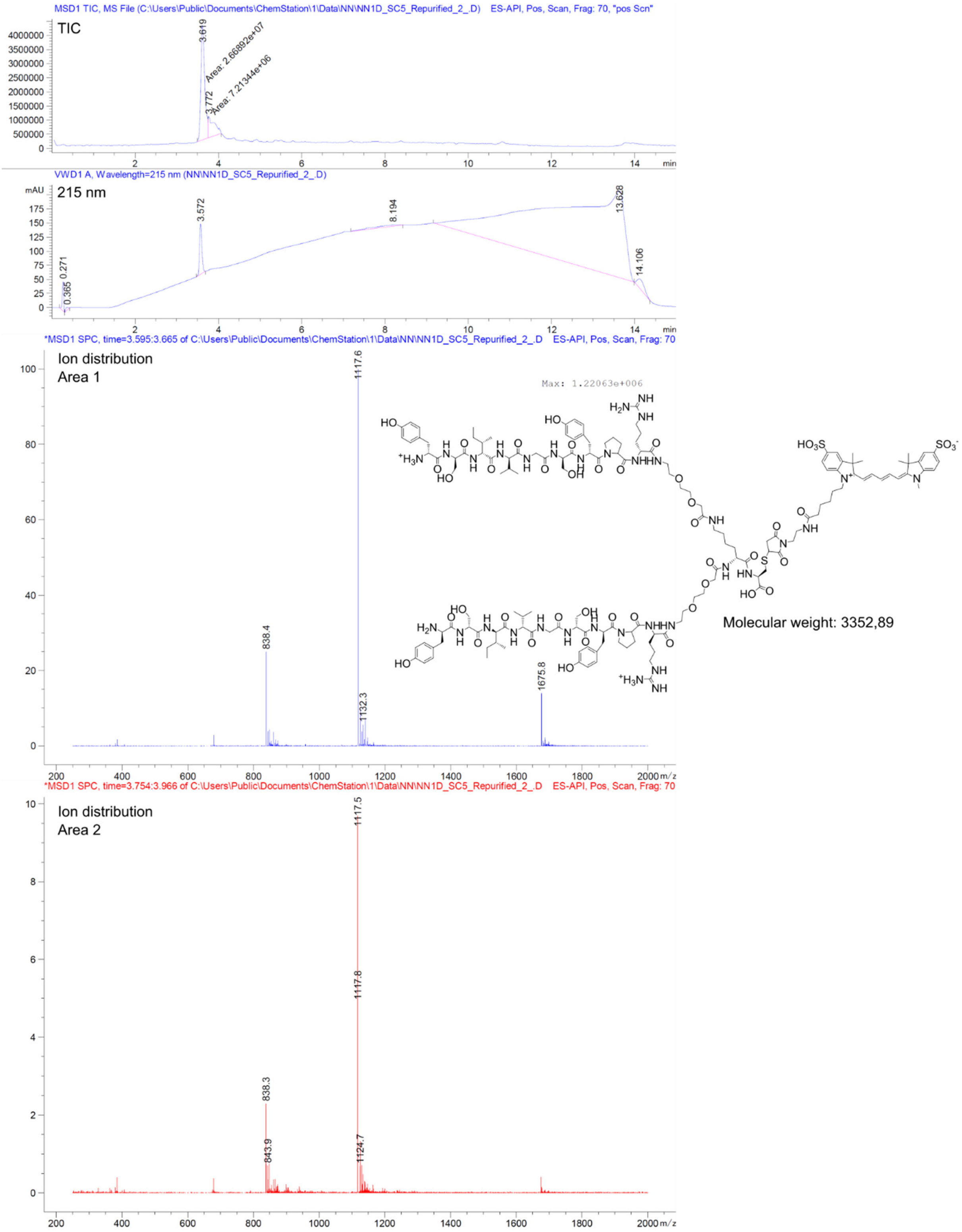
MSD1 TIC, MS File (C:\Users\Public\Documents\ChemStation\1\Data\NN\NN1D_SC5_Repurified_2_.D) ES-API, Pos, Scan, Frag: 70, "pos Scn"
TIC
3.619
Area: 2.66892e+07
3.772
Area: 7.21344e+06
VWD1 A, Wavelength=215 nm (NN\NN1D_SC5_Repurified_2_.D)
215 nm
mAU
0.271
0.365
3.572
8.194
13.628
14.106
min
*MSD1 SPC, time=3.595:3.665 of C:\Users\Public\Documents\ChemStation\1\Data\NN\NN1D_SC5_Repurified_2_.D ES-API, Pos, Scan, Frag: 70
Ion distribution
Area 1
1117.6
838.4
1132.3
1675.8
Max: 1.22063e+006
Molecular weight: 3352,89
m/z
*MSD1 SPC, time=3.754:3.966 of C:\Users\Public\Documents\ChemStation\1\Data\NN\NN1D_SC5_Repurified_2_.D ES-API, Pos, Scan, Frag: 70
Ion distribution
Area 2
1117.5
1117.8
838.3
843.9
1124.7
m/z

Sylite check-up after 12-month storage in DMSO stock solution at -20°C

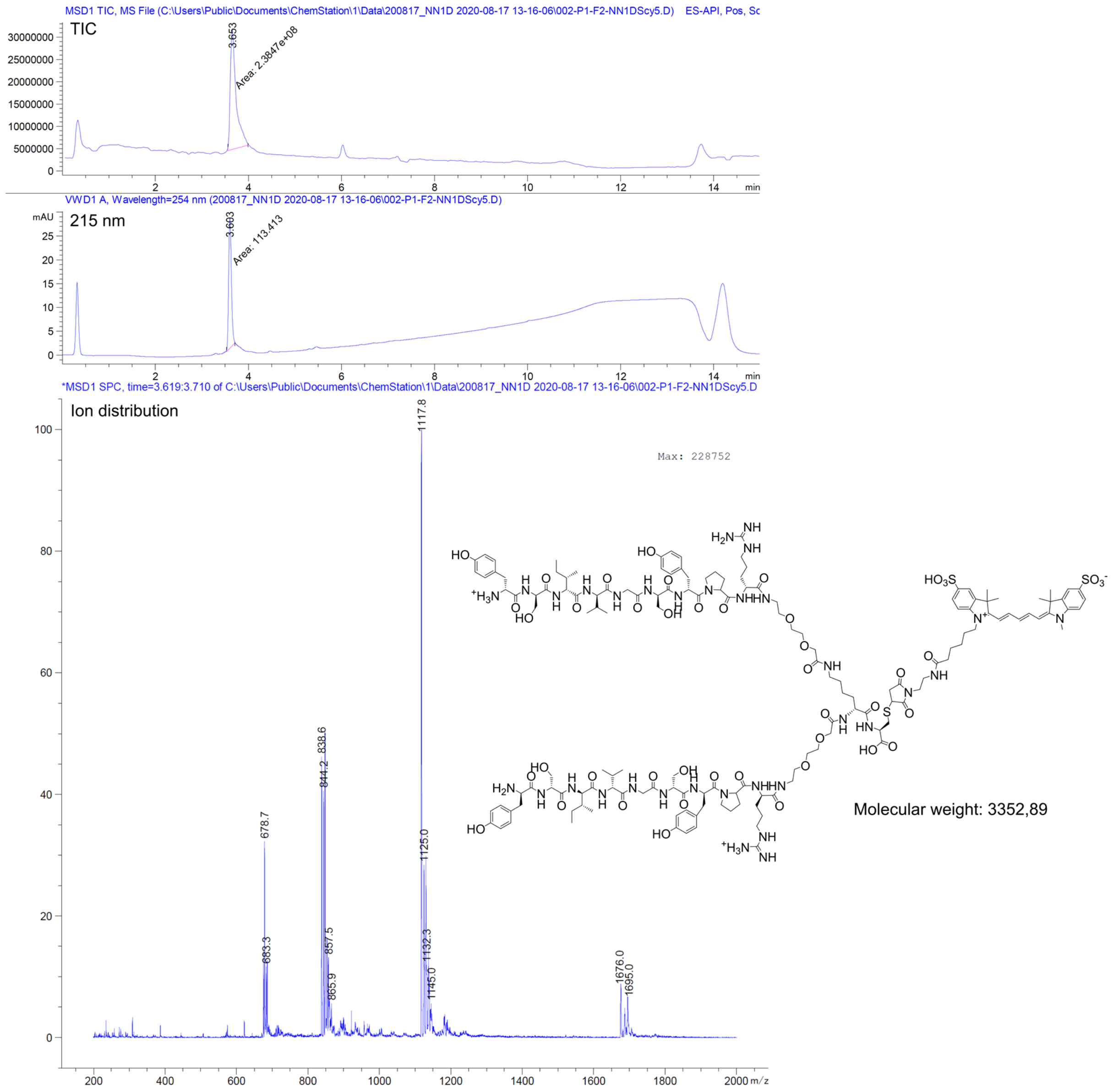

SyliteCy3

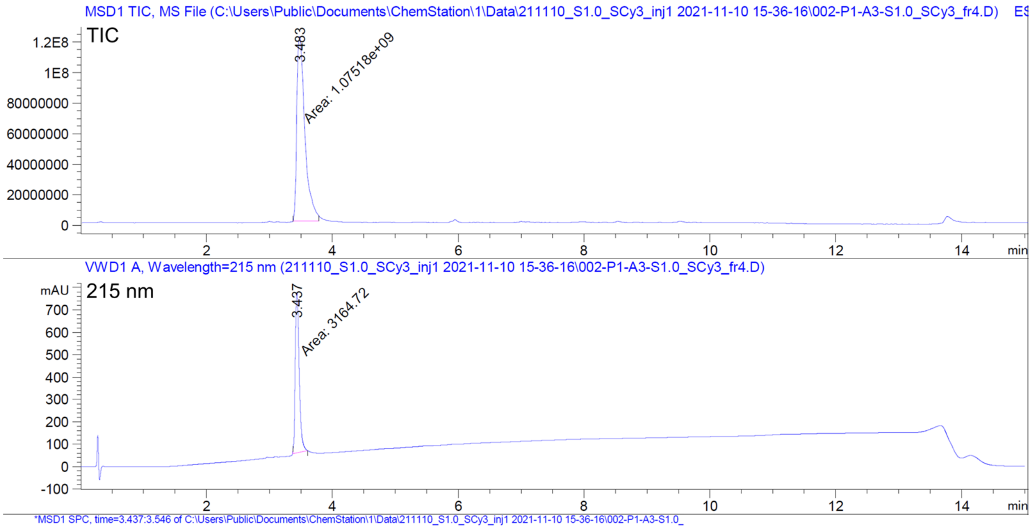

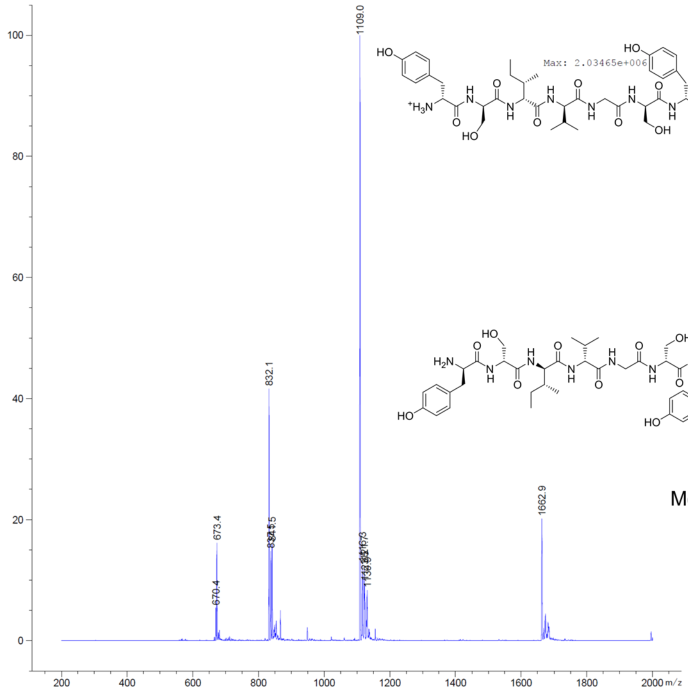

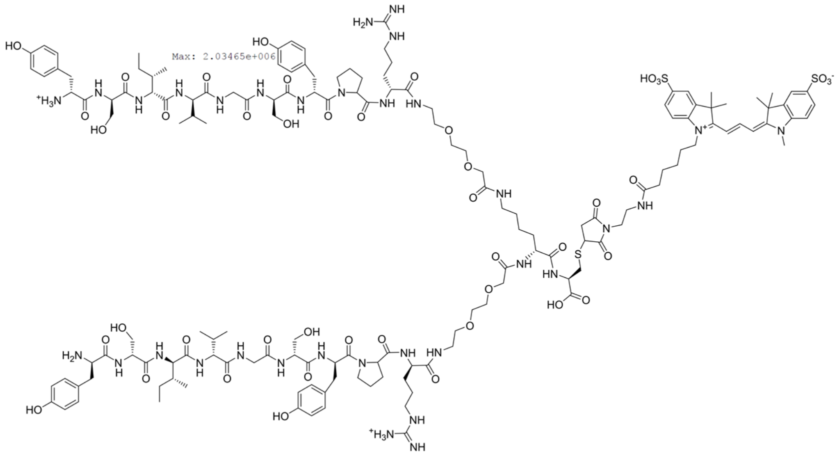

Molecular Weight: 3326.85

VK16

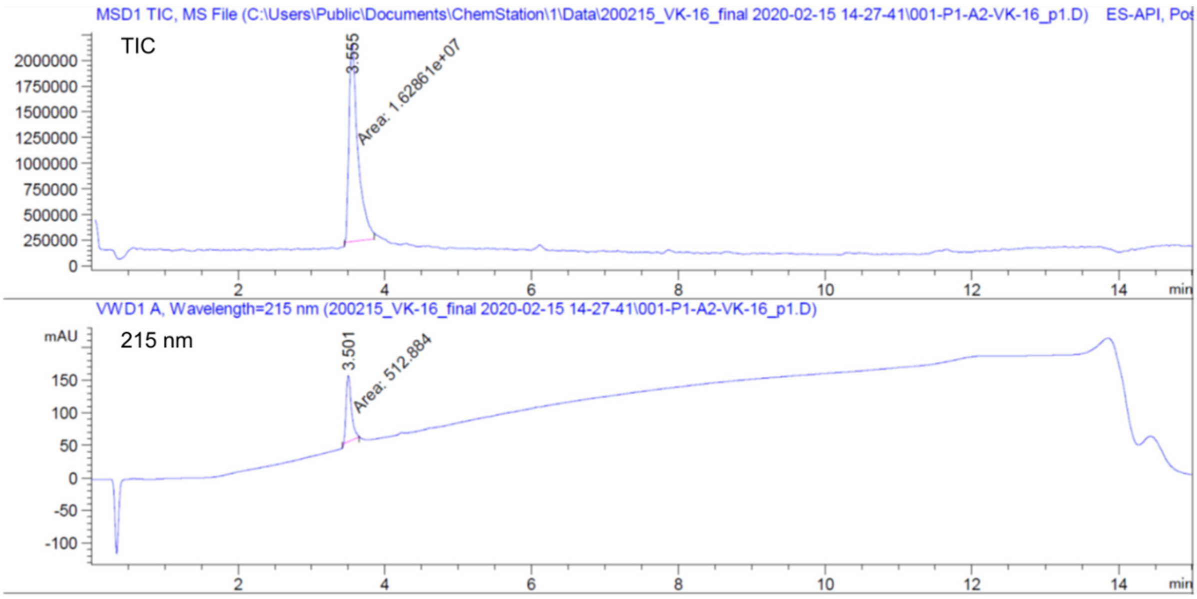

MSD1 TIC, MS File (C:\Users\Public\Documents\ChemStation\1\Data\200215_VK-16_final 2020-02-15 14-27-41\001-P1-A2-VK-16_p1.D) ES-API, Pos
TIC
3.555
Area: 1.62861e+07
VWD1 A, Wavelength=215 nm (200215_VK-16_final 2020-02-15 14-27-41\001-P1-A2-VK-16_p1.D)
215 nm
mAU
3.501
Area: 512.884
min

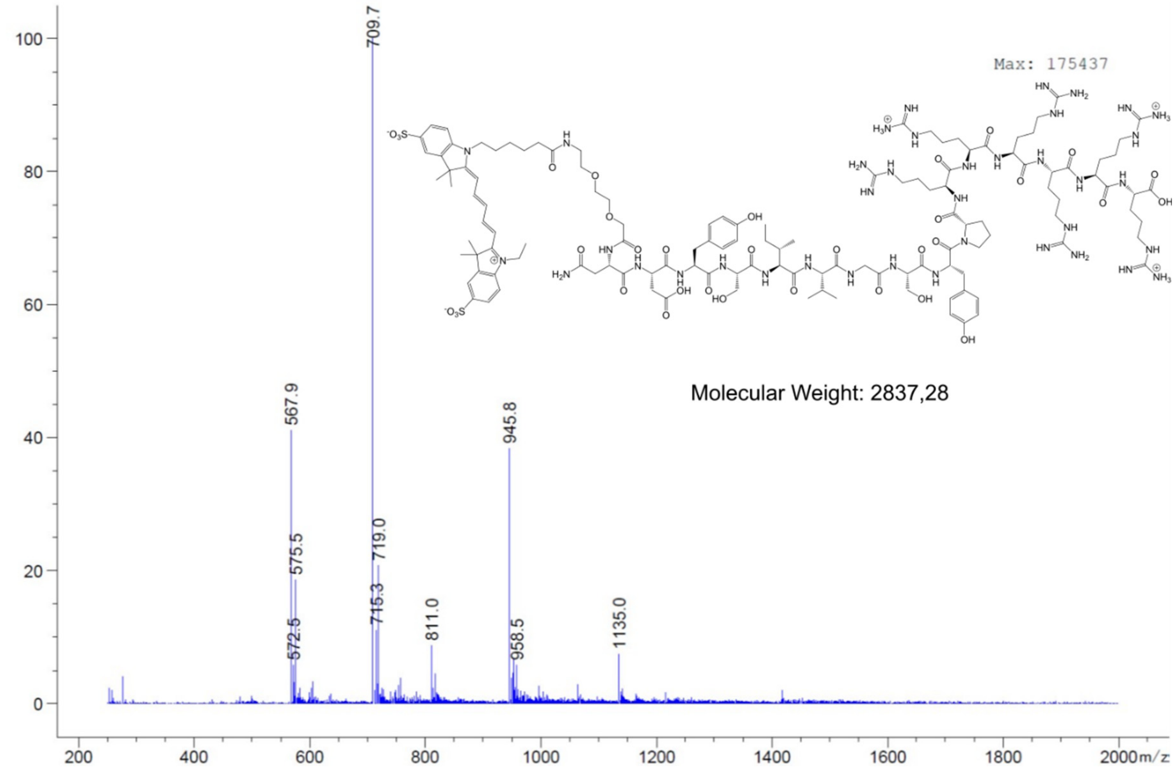

709.7
Max: 175437
567.9
945.8
575.5
719.0
715.3
572.5
811.0
958.5
1135.0
m/z
Molecular Weight: 2837,28

VK17

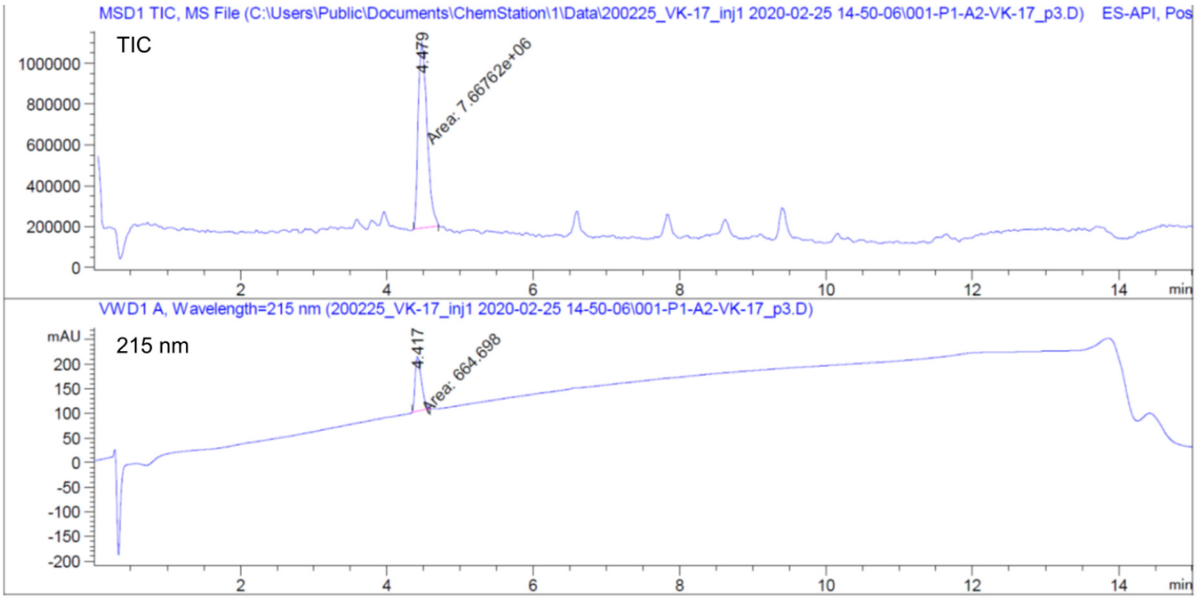
MSD1 TIC, MS File (C:\Users\Public\Documents\ChemStation\1\Data\200225_VK-17_inj1 2020-02-25 14-50-06\001-P1-A2-VK-17_p3.D) ES-API, Pos
TIC
4.479
Area: 7.66762e+06
VWD1 A, Wavelength=215 nm (200225_VK-17_inj1 2020-02-25 14-50-06\001-P1-A2-VK-17_p3.D)
215 nm
mAU
4.417
Area: 664.698
min

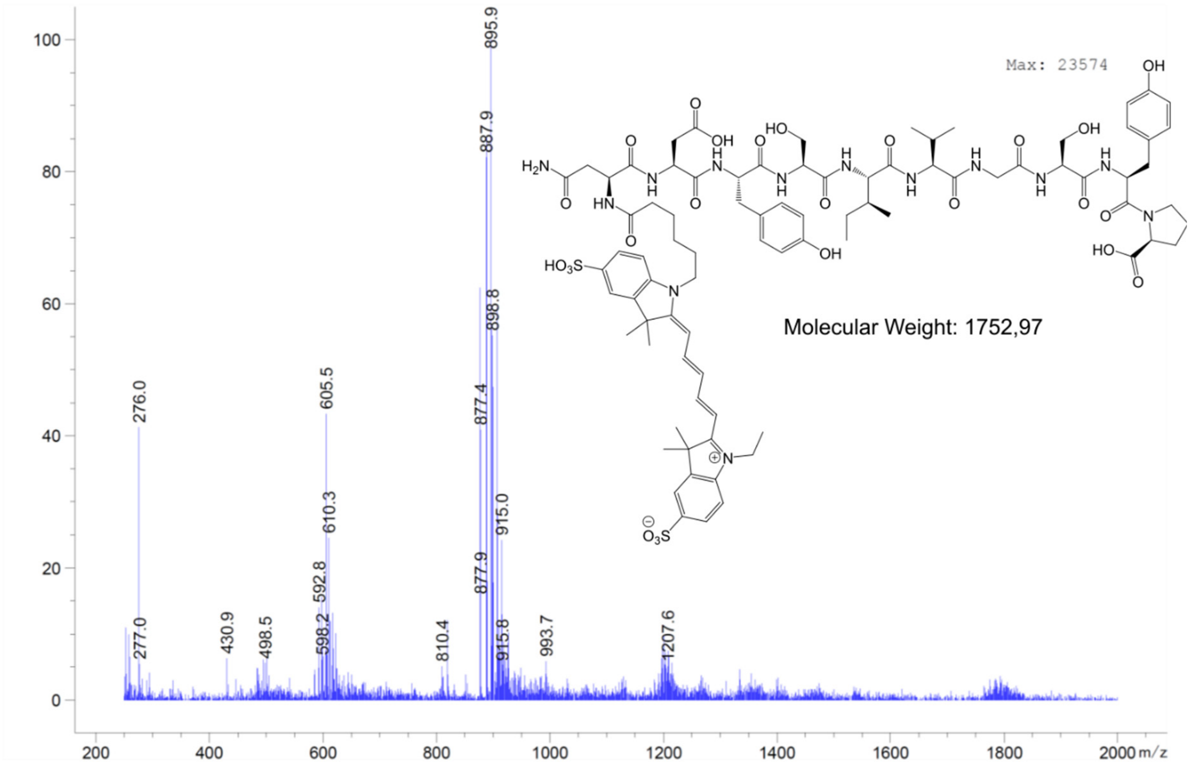
Max: 23574
895.9
887.9
898.8
877.4
915.0
877.9
915.8
993.7
1207.6
810.4
605.5
610.3
592.8
598.2
276.0
277.0
430.9
498.5
m/z
Molecular Weight: 1752,97

VK18

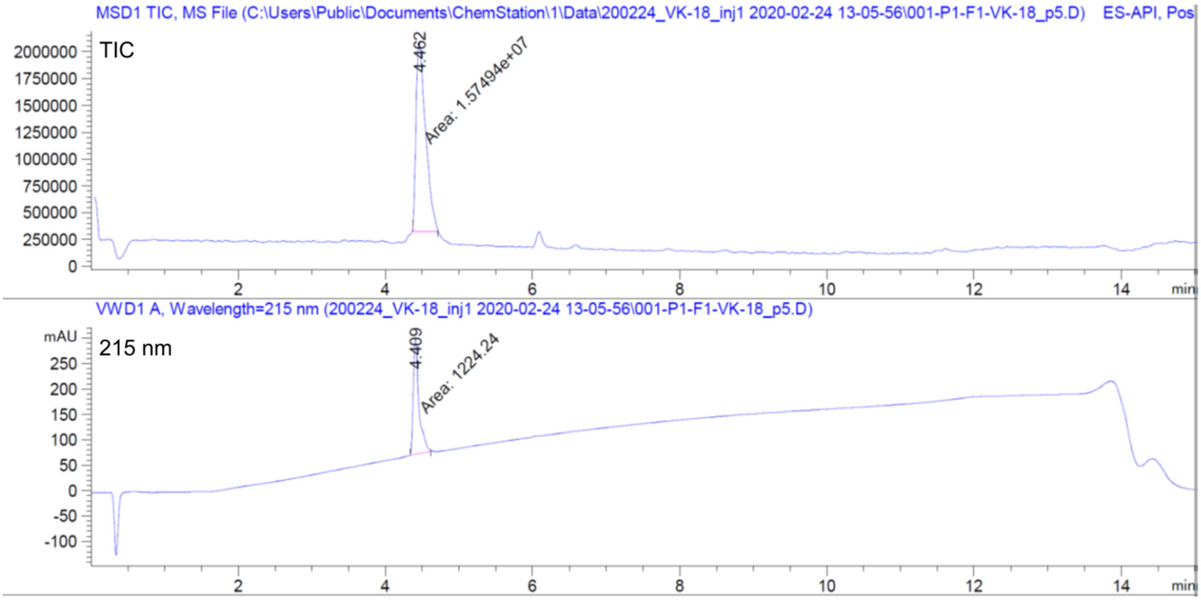

MSD1 TIC, MS File (C:\Users\Public\Documents\ChemStation\1\Data\200224_VK-18_inj1 2020-02-24 13-05-56\001-P1-F1-VK-18_p5.D) ES-API, Pos
TIC
4.462
Area: 1.57494e+07
VWD1 A, Wavelength=215 nm (200224_VK-18_inj1 2020-02-24 13-05-56\001-P1-F1-VK-18_p5.D)
215 nm
mAU
4.409
Area: 1224.24
min

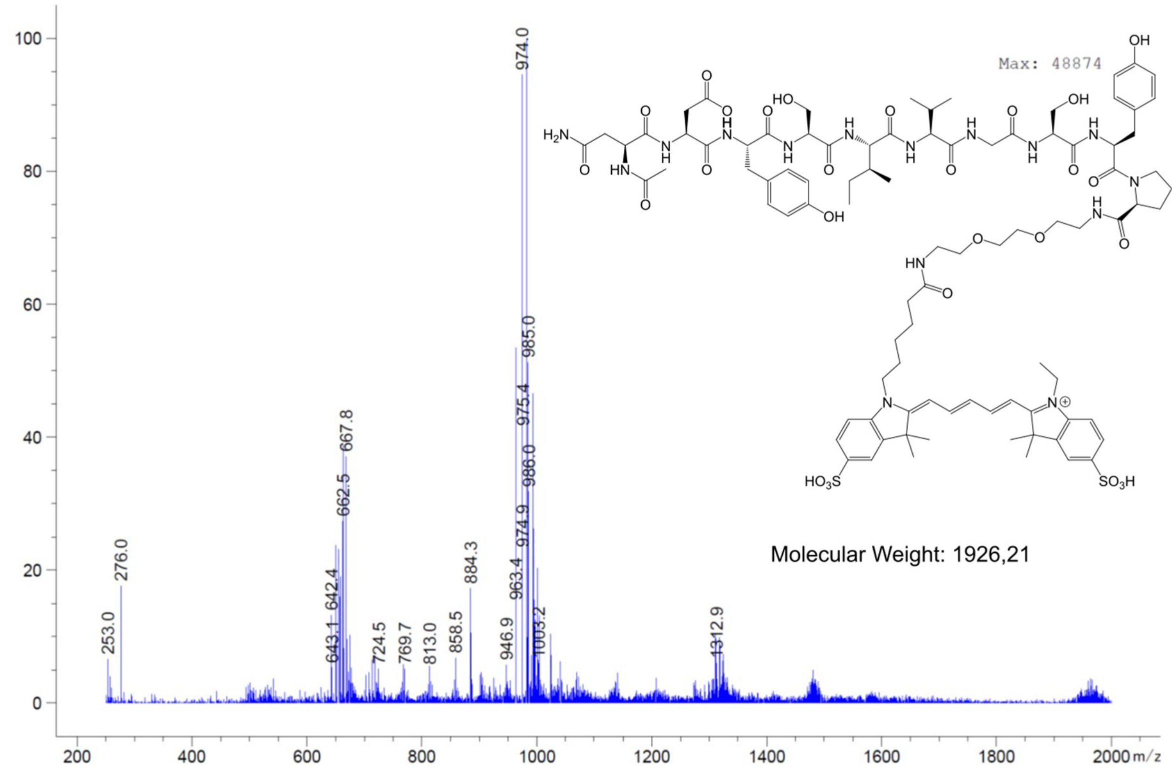

974.0
985.0
975.4
986.0
974.9
963.4
1003.2
667.8
662.5
642.4
643.1
724.5
769.7
813.0
858.5
884.3
946.9
276.0
253.0
1312.9
Max: 48874
m/z
Molecular Weight: 1926,21

VK20

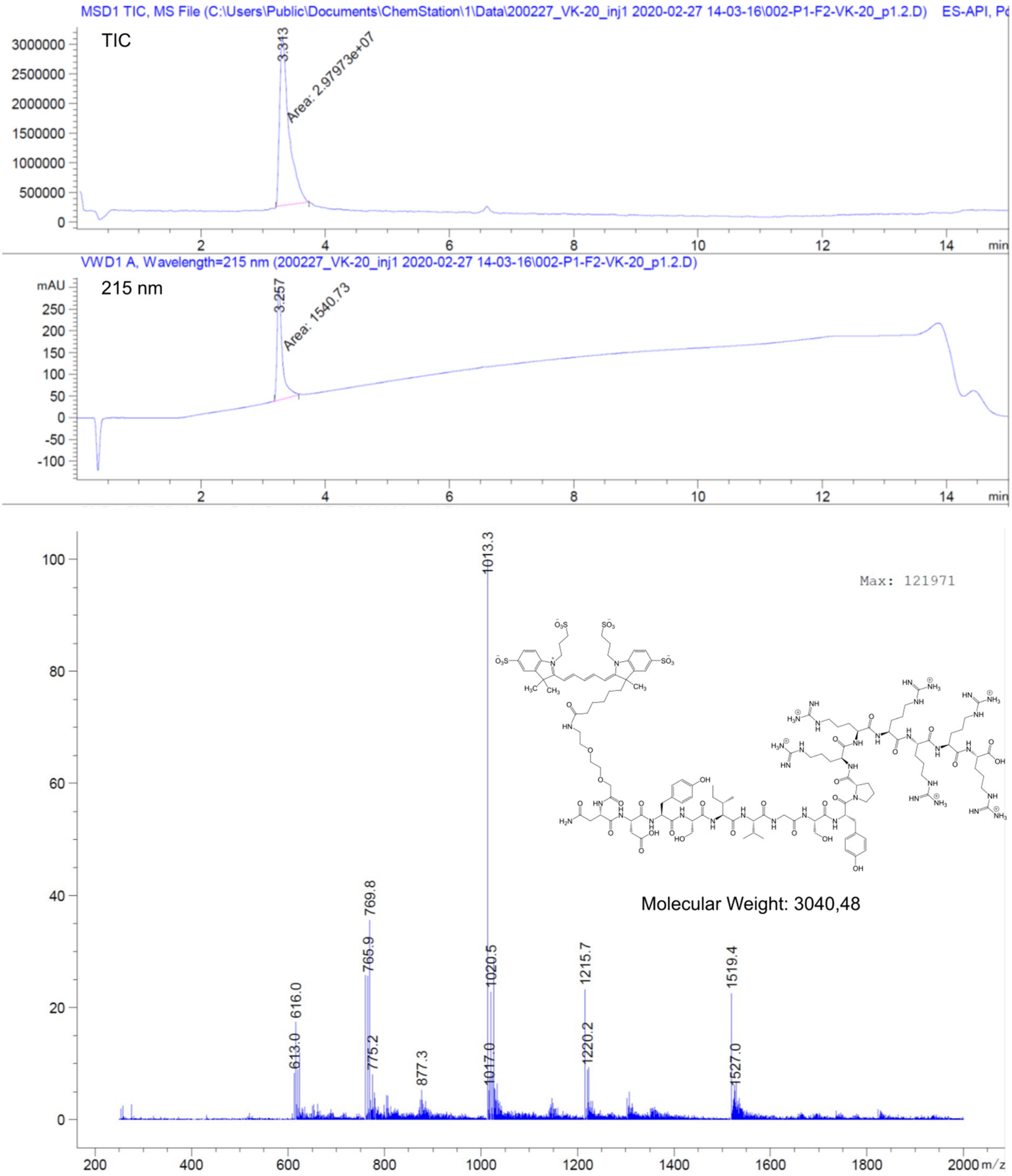
MSD1 TIC, MS File (C:\Users\Public\Documents\ChemStation\1\Data\200227_VK-20_inj1 2020-02-27 14-03-16\002-P1-F2-VK-20_p1.2.D) ES-API, Po
TIC
3.313
Area: 2.97973e+07
VWD1 A, Wavelength=215 nm (200227_VK-20_inj1 2020-02-27 14-03-16\002-P1-F2-VK-20_p1.2.D)
215 nm
3.257
Area: 1540.73
Max: 121971
1013.3
769.8
765.9
1020.5
1215.7
1519.4
616.0
613.0
775.2
877.3
1017.0
1220.2
1527.0
Molecular Weight: 3040,48

VK21

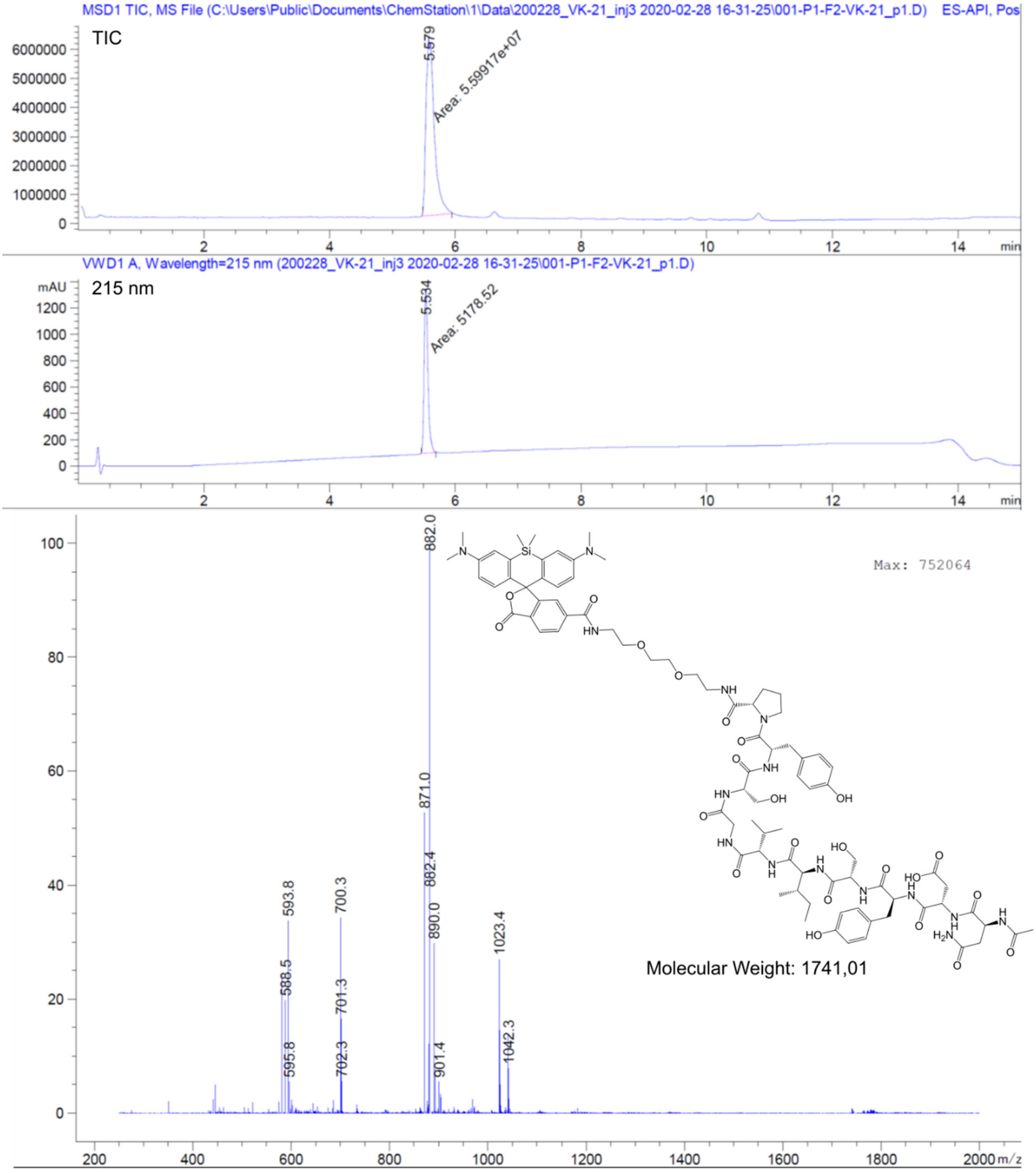
MSD1 TIC, MS File (C:\Users\Public\Documents\ChemStation\1\Data\200228_VK-21_inj3 2020-02-28 16-31-25\001-P1-F2-VK-21_p1.D) ES-API, Pos
TIC
5.579
Area: 5.59917e+07
VWD1 A, Wavelength=215 nm (200228_VK-21_inj3 2020-02-28 16-31-25\001-P1-F2-VK-21_p1.D)
215 nm
mAU
5.534
Area: 5178.52
min
Max: 752064
882.0
871.0
882.4
890.0
901.4
593.8
588.5
595.8
700.3
701.3
702.3
1023.4
1042.3
m/z
Molecular Weight: 1741,01

VK22

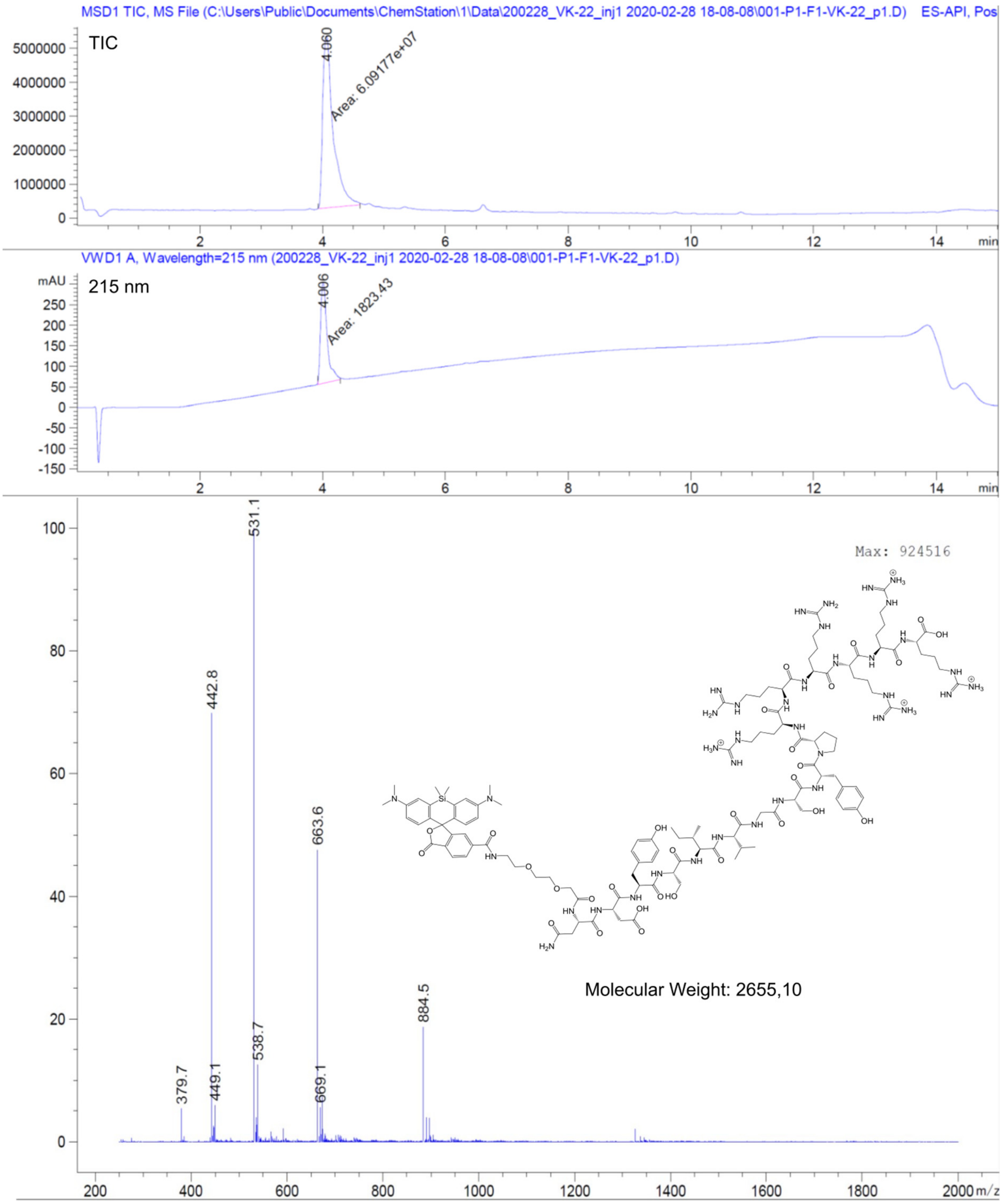
MSD1 TIC, MS File (C:\Users\Public\Documents\ChemStation\1\Data\200228_VK-22_inj1 2020-02-28 18-08-08\001-P1-F1-VK-22_p1.D) ES-API, Pos
TIC
4.060
Area: 6.09177e+07
min
VWD1 A, Wavelength=215 nm (200228_VK-22_inj1 2020-02-28 18-08-08\001-P1-F1-VK-22_p1.D)
mAU
215 nm
4.006
Area: 1823.43
min
Max: 924516
531.1
442.8
663.6
884.5
538.7
379.7
449.1
669.1
m/z
Molecular Weight: 2655,10

DVK1

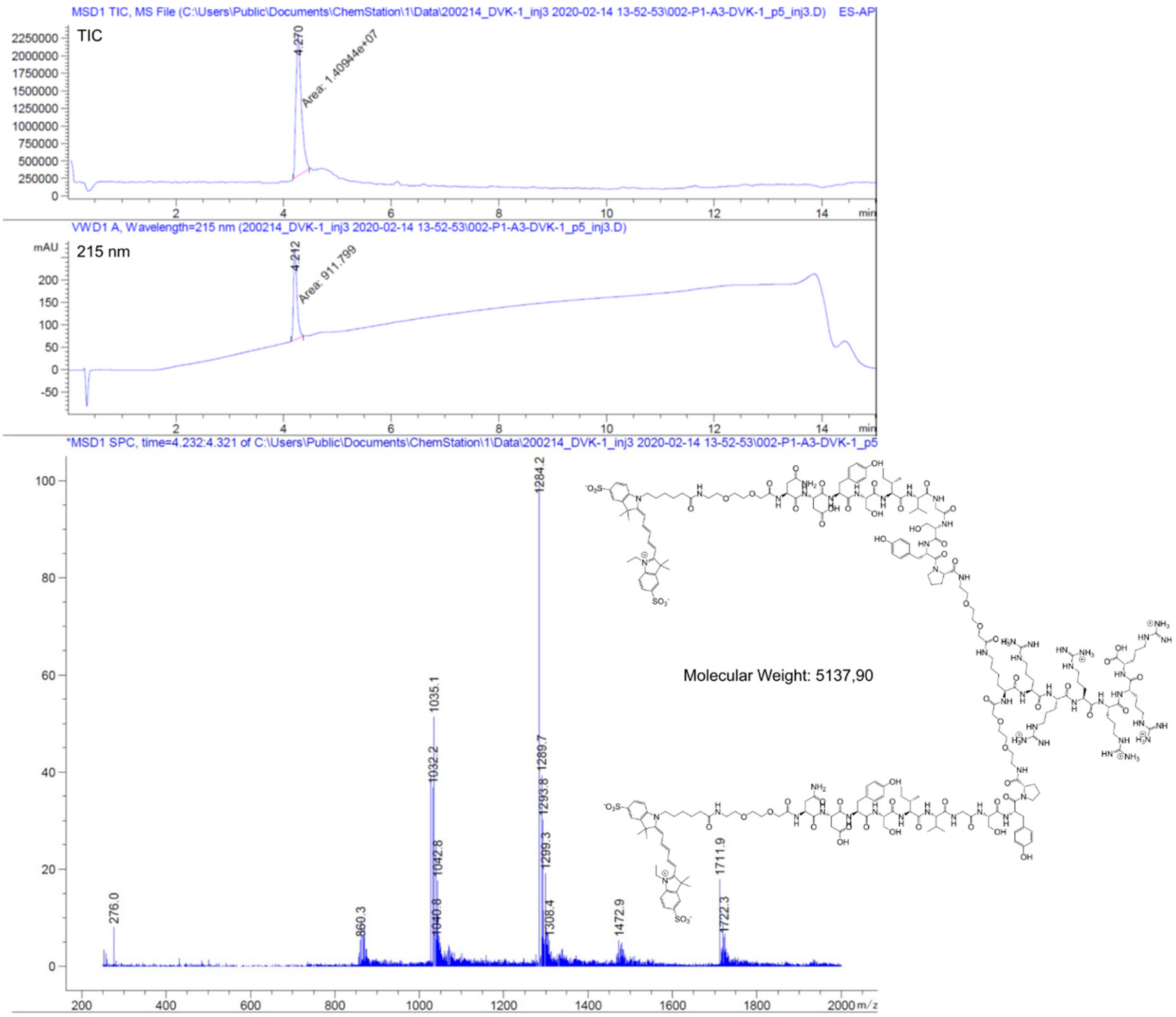
MSD1 TIC, MS File (C:\Users\Public\Documents\ChemStation\1\Data\200214_DVK-1_inj3 2020-02-14 13-52-53\002-P1-A3-DVK-1_p5_inj3.D) ES-API
TIC
4.270
Area: 1.40944e+07
VWD1 A, Wavelength=215 nm (200214_DVK-1_inj3 2020-02-14 13-52-53\002-P1-A3-DVK-1_p5_inj3.D)
215 nm
4.212
Area: 911.799
*MSD1 SPC, time=4.232:4.321 of C:\Users\Public\Documents\ChemStation\1\Data\200214_DVK-1_inj3 2020-02-14 13-52-53\002-P1-A3-DVK-1_p5
1284.2
1035.1
1032.2
1289.7
1293.8
1042.8
1299.3
1711.9
276.0
860.3
1040.8
1308.4
1472.9
1722.3
Molecular Weight: 5137,90

DVK2

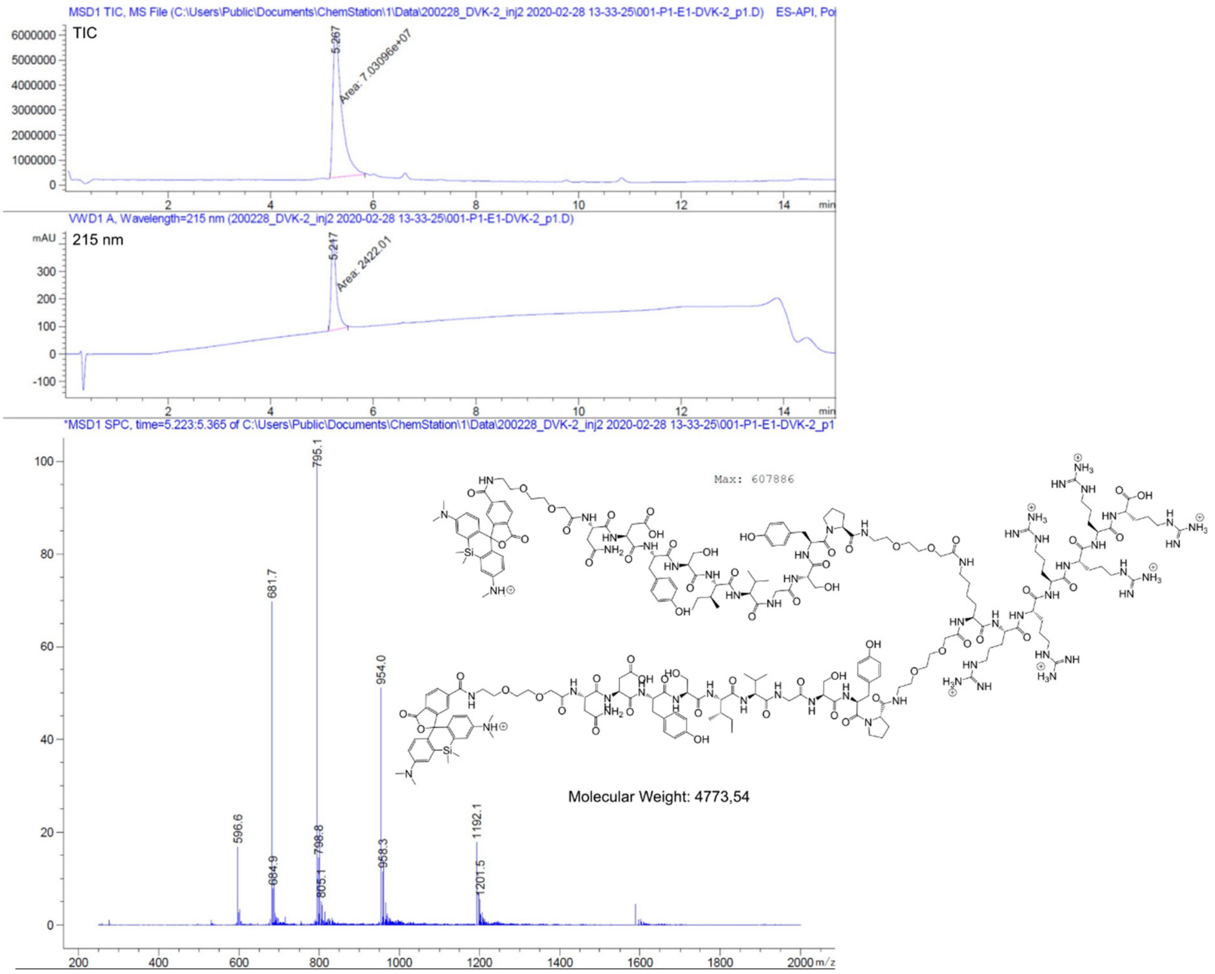
MSD1 TIC, MS File (C:\Users\Public\Documents\ChemStation\1\Data\200228_DVK-2_inj2 2020-02-28 13-33-25\001-P1-E1-DVK-2_p1.D) ES-API, Po
TIC
5.267
Area: 7.03096e+07
VWD1 A, Wavelength=215 nm (200228_DVK-2_inj2 2020-02-28 13-33-25\001-P1-E1-DVK-2_p1.D)
215 nm
5.217
Area: 2422.01
*MSD1 SPC, time=5.223:5.365 of C:\Users\Public\Documents\ChemStation\1\Data\200228_DVK-2_inj2 2020-02-28 13-33-25\001-P1-E1-DVK-2_p1
Max: 607886
795.1
681.7
954.0
596.6
684.9
798.8
805.1
958.3
1192.1
1201.5
Molecular Weight: 4773,54
